# Reaction of methylene blue with OH radicals in the aqueous environment: mechanism, kinetics, products and risk assessment†

**DOI:** 10.1039/d4ra05437g

**Published:** 2024-08-27

**Authors:** Quan V. Vo, Luu Thi Thu Thao, Tran Duc Manh, Mai Van Bay, Bich-Tram Truong-Le, Nguyen Thi Hoa, Adam Mechler

**Affiliations:** a The University of Danang – University of Technology and Education Danang 550000 Vietnam vvquan@ute.udn.vn; b The University of Danang – University of Sciences and Education Danang 550000 Vietnam; c Department of Science and International Cooperation, The University of Danang Danang 550000 Vietnam; d Department of Biochemistry and Chemistry, La Trobe University Victoria 3086 Australia

## Abstract

Methylene Blue (MB) is an industrial chemical used in a broad range of applications, and hence its discharge is a concern. Yet, the environmental effects of its degradation by HO˙ radicals have not been fully studied yet. This study employs quantum chemical calculations to investigate the two-step degradation of MB by HO˙ radicals in aqueous environments. It was found that MB undergoes a rapid reaction with the HO˙ radical, with an overall rate constant of 5.51 × 10^9^ to 2.38 × 10^10^ M^−1^ s^−1^ and has a rather broad lifetime range of 11.66 hours to 5.76 years in water at 273–383 K. The calculated rate constants are in good agreement with the experimental values (*k*_calculation_/*k*_experimental_ = 2.62, pH > 2, 298 K) attesting to the accuracy of the calculation method. The HO˙ + MB reaction in water followed the formal hydrogen transfer and radical adduct formation mechanisms, yielding various intermediates and products. Based on standard tests these intermediates and some of the products can pose a threat to aquatic organisms, including fish, daphnia, and green algae, they have poor biodegradability and have the potential to induce developmental toxicity. Hence MB in the environment is of moderate concern depending on the ratio of safe to harmful breakdown products.

## Abstract 2

Methylene blue in the environment is of moderate concern, depending on the ratio of safe to harmful breakdown products.

## Graphical abstract

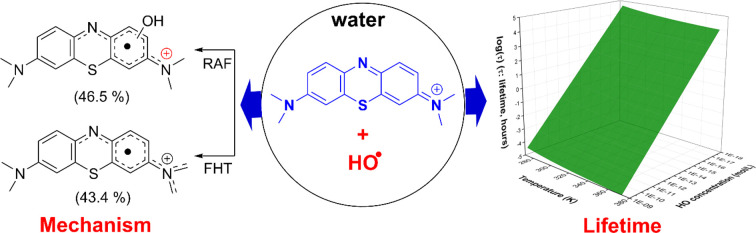

## 1. Introduction

**Fig. 1:**
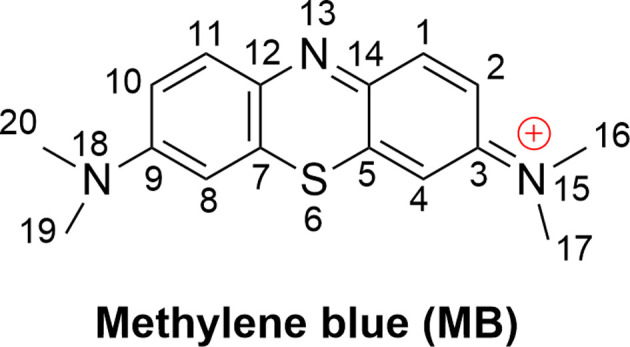
Structure of MB.

Methylene blue (MB, Fig. 1) is an aromatic heterocyclic dye that is extensively used in industry, especially for coloring silk, cotton, wool, paper, and similar materials.^1–3^MB is a cationic dye that is difficult to degrade in natural processes;^4,5^ therefore, it has the potential to disrupt ecological systems and in an uncontrolled release to cause a variety of health hazards to humans,^2,3^ albeit it also has well-established clinical uses.^6–8^ Consistently, the elimination of MB from drinking water is a critical concern.^9–14^

Several techniques such as UV-based advanced oxidation processes (AOPs), photocatalytic methods, and ultra-sonic treatments are in use for the decomposition of toxic chemicals including MB from waste water.^1,11,15–20^ The pulsed power technique can entirely remove MB in alkaline solutions within 6–8 minutes.^11^MB degradation can be greatly enhanced by using the vacuum-ultraviolet/ultraviolet/persulfate process in comparison to the conventional ultraviolet/persulfate process.^15^ The degradation of MB in water treatment can also be enhanced by using photocatalysts such as TiO_2_ and ZnO; ultrasonic irradiation is also an efficient method.^1,17,20–22^ Arguably the simplest approach is supplementing naturally occurring reactive oxygen species (ROS, including HO˙, SO_4_˙^−^, Cl˙, ClO˙, HOO˙ and O_2_˙^−^) for the AOPs technique. Of these, HO˙ radicals are known to be the primary active substances for MB oxidation.^16,19^

HO˙ radicals are key natural oxidizing species in natural aquifers due to their strong reactivity towards organic substrates,^23,24^ playing a substantial role in defining the environmental fate of industrial chemicals despite their low steady-state concentrations ranging from 10^−18^ to 10^−15^ M.^25–27^ Therefore, principal photo-oxidation products are expected to form with the involvement of HO˙ in the self-cleaning process of water in nature. Multiple empirical studies have been carried out to determine the rate of the MB + ˙OH reaction and to identify the products of MB oxidation that occur in an aqueous solution through the introduction of ˙OH.^9,10,15,16,28–30^ The rate constant for the interaction of MB with ˙OH at ambient temperature was determined to be 3.8 × 10^9^ M^−1^ s^−1^.^15^ This indicates that MB reacts quickly with HO˙ in water. In spite of the promising results, no further studies have been performed on the kinetics of the process.

The HO˙ + MB reaction proceeds principally through the radical adduct formation (RAF) mechanism of HO˙ radicals into the aromatic ring. Therefore the predominant intermediates of the HO˙ + MB reaction are the adduct cations detected by mass spectrometry at *m*/*z* = 300,^28^ whereas a two-step reaction of MB with HO˙ radicals yields products at *m*/*z* = 316.^10,11,28^ To explain the mass spectrometry results the addition of HO˙ radicals into the C1 and C11 positions was proposed.^2,10,11^ However, there is no evidence supporting this mechanism. Furthermore, it was noted that the degradation of MB occurs slowly in the natural environment and thus the formation of intermediates that may cause toxicity for organic molecules cannot be neglected. Yet the safety of these intermediates has not attracted any attention thus far.

Quantum chemical calculations are well established as a reliable method for determining thermodynamic and kinetic properties of chemical reactions, including radical reactions.^31–35^ Here we present a computational study of the thermodynamic and kinetic properties of the hydroxyl radical reactions that involve MB and related compounds, as well as the intermediate product reactions that take place within the specified environmental conditions. The toxicity, developmental toxicity, mutagenicity, bioconcentration, and biodegradability of MB and its degradation products were also evaluated.

## 2. Computational methods

Kinetic calculations were performed using the Quantum Mechanics-based Overall Free Radical Scavenging Activity (QM-ORSA) protocol^31^ which is directly applicable in this case.^36,37^ Eqn (1) was the basis of calculating the rate constant (*k*) by the application of transition state theory (TST) under standard conditions of 1 M at varying ambient temperatures (253–323 K for the gas phase and 283–323 K for water).^38–44^1
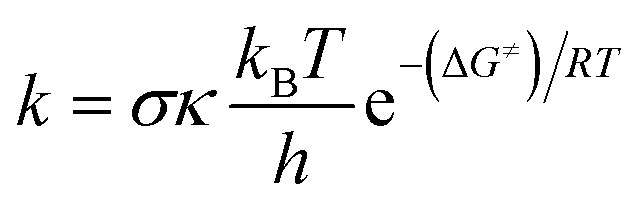


The reaction symmetry number is denoted by *σ*.^45,46^ Tunneling corrections are represented by *κ* and were calculated using the Eckart barrier,^47^*k*_B_ represents the Boltzmann constant, *h* is the Planck constant, and Δ*G*^≠^ is the Gibbs free energy of activation. The Marcus theory was used to determine the reaction barriers of single electron transfer (SET) reactions in the solvent.^48,49^ Eqn (2) and (3) were used to calculate the Δ*G*^≠^ for the SET reaction.2
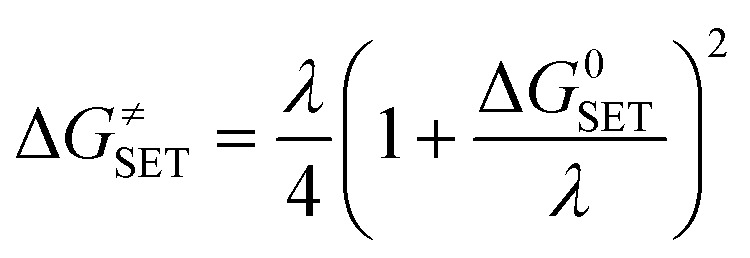

3 *λ* ≈ Δ*E*_SET_ − Δ*G*^0^_SET_

For SET reaction, the nonadiabatic energy difference between the reactants and the products is represented by Δ*E*_SET_, while the conventional Gibbs free energy change of the reaction is denoted by Δ*G*^0^_SET_.^50,51^ An adjustment was made for rate constants around the diffusion limit.^47^ The steady-state Smoluchowski rate constant (*k*_D_) was estimated from the literature, and the apparent rate constants (*k*_app_) for an irreversible bimolecular diffusion-controlled process in solvents^52^ were computed using Collins–Kimball theory.^50,53^4
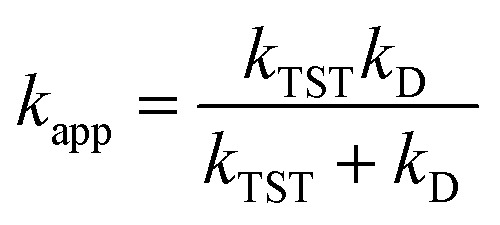

5 *k*_D_ = 4π*R*_AB_*D*_AB_*N*_A_

*D*_AB_ = *D*_A_ + *D*_B_ (denotes the mutual diffusion coefficient of A and B),^52,54^ where *D*_A_ or *D*_B_ is obtained using the Stokes–Einstein formulation (6).^55,56^6
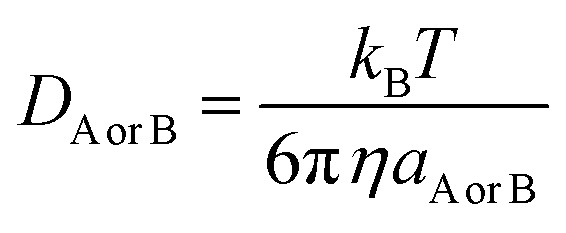
*η* is the viscosity of the solvent (*i.e. η*(H_2_O) = 8.91 × 10^−4^ Pa s) and *a* is the radius of the solute.

Energy minimization was applied to all conformers of species with multiple conformers; the conformer with the lowest electronic energy was used in the study.^57^ Each transition stage was characterized by the exclusive existence of a single imaginary frequency. Calculations of intrinsic coordinates were conducted in order to verify the accurate connection between each transition state and the pre- and post-complexes. In addition, the pre- and post-complexes were incorporated into the kinetic calculations.^32,58^

The calculations for this work were conducted using the Gaussian 16 software package^59^ at the M06-2X/6-311++G(d,p) level of theory, known for providing precise thermodynamics and kinetics results given the current computational resources.^37,60,61^ The SMD methodology was employed to simulate the solvent effects of water,^60^ a typical method for assessing the radical scavenging properties of antioxidants. The calculated values showed moderate differences compared to the experimental results, with a *k*_calc_/*k*_exp_ ratio ranging from 0.3 to 2.9.^32,37,43,62–66^

The ecotoxicity assessment was carried out using the Ecological Structure-Activity Relationship Model (ECOSAR V2.0), which has a proven efficacy in assessing the ecotoxicity of organic contaminants.^33,67–70^ The developmental toxicity and mutagenicity of MB and its transformed products were evaluated through toxicological analysis utilizing the T.E.S.T. (Toxicity Estimation Software Tool) toxicity assessment software.^71^ Utilizing the BCFBAF module of EPISUITE, the biological concentration factors (BCFs) produced by the conversion products of MB degradation were calculated. Utilizing the BIOWIN 3 & 4 model integrated into the EPISUITE software,^72^ an assessment was made of the biodegradability of MB and its degradation products.

## 3. Results and discussion

### 3.1. Reaction of hydroxyl radical with MB in water

#### 3.1.1. The initial reaction of MB with HO˙ in water

Deprotonation plays a crucial role in the interaction between organic molecules and free radical species in aqueous media.^33,73^ Hence, when evaluating the efficacy of water in eliminating radicals, it is essential to consider the deprotonation of MB. The p*K*_a_ value of MB has been previously documented as zero (Fig. S1, ESI†).^74^ Thus, in the natural aqueous environment (pH > 2), diphenylamine exists in a cation state (MB, Fig. S1, ESI†). Therefore, this state should be used to evaluate the kinetics of the MB + HO˙ reaction in the aqueous environment.

The reaction between MB and HO˙ can occur *via* the radical adduct formation (RAF), formal hydrogen transfer (FHT), or SET, as per eqn (7)–(9):^33,36,37,75^

7 RAF: MB–H + HO˙ → [HO–MB–H]˙

8 FHT: MB–H + HO˙ → MB˙ + H_2_O

9 SET: MB–H + HO˙ → [MB–H]˙^+^ + HO^−^

**Fig. 2:**
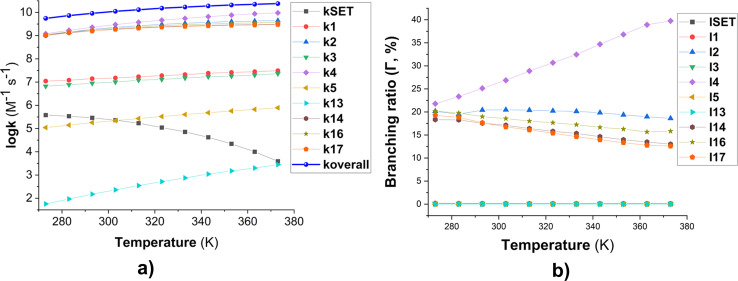
Temperature dependence of apparent rate constants (log *k*) in water in the range of 273–373 K ((a) the MB + HO˙ reactions; (b) branching ratio (*Γ*, %) of the MB + HO˙ reactions; *k*_i_ = 1–17: the rate constants of the positions in Table 1).

**Fig. 3:**
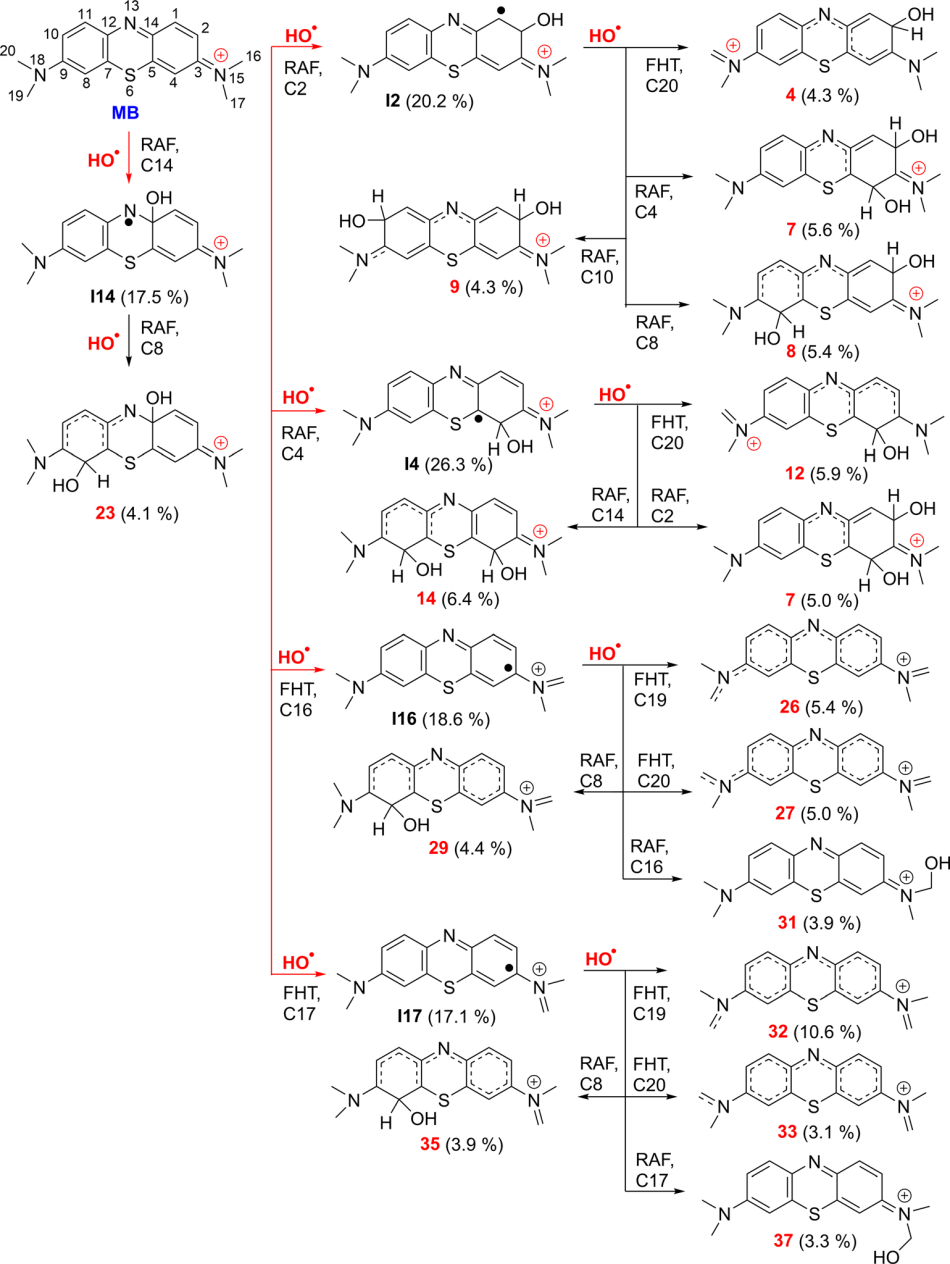
The selected mechanisms and % products (*Γ* ≥ 3%) of the two steps HO˙ + MB reactions in water at 298.15 K.

The kinetics of these reactions were calculated and are presented in Table 1, whereas the effect of temperature on the degradation of MB is shown in Fig. 2. We found that the reaction between MB and HO˙ radicals had an overall rate constant (*k*_overall_) of 1.02 × 10^10^ M^−1^ s^−1^. This is consistent with the observed experimental rate constant (*k*_exp_ = 3.8 × 10^9^ M^−1^ s^−1^).^15^ The FHT reactions at C16/17–H accounted for approximately 35.7% of the total rate constant, while the SET reaction had no contribution (*Γ* = 0%) to the MB + HO˙ reaction. The MB + HO˙ reaction was predominantly taking place *via* the RAF reactions, which represented 64.2% of the reaction. The RAF (C2, C4, and C14) took place rapidly with a rate constant similar to the rate of diffusion (*k* ≈ 10^9^ M^−1^ s^−1^) and formed the cations [MB–OH]^+^ (*m*/*z* = 300) that were also observed in the experimental studies.^11,28^ On the other hand, the RAF reaction at the C1, C3, and C5 sites had moderate reaction rates (*k* = 10^5^ to 10^7^ M^−1^ s^−1^) and did not have any impact on the overall reaction. Therefore, the formation of cations [MB–OH]^+^ (*m*/*z* = 300) can occur by introducing HO˙ radicals to the C2, C4, or C14 sites (Fig. 3), but not at the C1 position, this is also consistent with prior research.^11,28^ The likely cause for the preferential adduct formation could be attributed to the presence of the electron-donating group N(CH_3_)_2_ in either the *ortho*- or *para*-position. This group has the potential to stabilize both the transition states and the resultant radicals.

**Table 1:** Computed Δ*G*^≠^ (kcal mol^−1^), *κ*, *k*_app_, *k*_overall_ (M^−1^ s^−1^), and *Γ* (%) at 298.15 K, in the HO˙ + MB in water^a^

Mechanisms	Positions	Δ*G*^≠^	*κ*	*k*_app_	*Γ*	Intermediates
SET		10.1	0.4^a^	2.50 × 10^5^	0.0	ISET
FHT	C16–H	5.3	1.0	1.90 × 10^9^	18.6	I16
	C17–H	5.4	1.0	1.75 × 10^9^	17.1	I17
RAF	C1	8.2	1.2	1.50 × 10^7^	0.1	I1
	C2	4.1	1.0	2.07 × 10^9^	20.2	I2
	C3	8.4	1.2	9.50 × 10^6^	0.1	I3
	C4	1.8	1.0	2.69 × 10^9^	26.3	I4
	C5	10.8	1.2	2.00 × 10^5^	0.0	I5
	C14	4.6	1.0	1.79 × 10^9^	17.5	I14
	N13	14.7	1.8	1.90 × 10^2^	0.0	I13
***k***_**overall**_				**1.02 × 10** ^**10**^		

a The nuclear reorganization energy (*λ*, in kcal mol^−1^).

The incorporation of HO˙ radicals into the N13 position is expected to be extremely rare with the highest activation energy (Δ*G*^≠^ = 14.7 kcal mol^−1^) and the lowest rate constant (*k* = 1.90 × 10^2^ M^−1^ s^−1^). Therefore, this reaction does not contribute to the destruction of MB by HO˙ radicals. The primary intermediates of the MB + HO˙ reaction were I2 (20.2%), I4 (26.3%), I14 (17.5%), I16 (18.6%), and I17 (17.1%), as indicated in Table 1 and Fig. 3. Therefore, these intermediates were used as a starting point for assessing the kinetics of the subsequent reaction step.

To examine the effect of temperature on the degradation of MB in water, we determined the rate constants for each reaction in the range of 273 to 383 K (Fig. 2). The rate constants for all reactions increase as the temperature rises with the exception of the SET reaction, where the rate decreased from 3.80 × 10^5^ to 3.90 × 10^3^ M^−1^ s^−1^. The overall rate constants exhibited a 4.33-fold increase, rising from 5.51 × 10^9^ to 2.38 × 10^10^ M^−1^ s^−1^ (Fig. 2a). The FHT and RAF (C2, C4, and C14) reactions defined the overall rate constants of the MB + HO˙ reaction at all temperatures investigated. However, the RAF reactions at C1, C3, C5, and N13 did not contribute to the degradation of MB by HO˙ radicals.

As the temperature increases, the major intermediates for the MB + HO˙ reaction, shown in Fig. 2b, exhibit varying branching ratios. Specifically, the percentages of I2 change from 20.2% to 18.6%, I4 changes from 21.8% to 39.7%, I14 goes from 18.3% to 13.0%, I16 changes from 20.2% to 15.8%, and I17 from 19.2% to 12.6%. Thus when the temperature climbed, the fraction of intermediate I4 grew, while the amount of all other intermediates decreased. Percentage-wise I4 is the main intermediate in all of the studied temperatures.

#### 3.1.2. The second step reaction of diphenylamine with HO˙ in water

To better understand the interaction between intermediates and HO˙ in water, a study was undertaken on the second step of the MB + HO˙ reaction. The results of the calculations are presented in Tables 2, S1 (ESI)† and Fig. 3.

**Table 2:** Computed Δ*G*^≠^ (kcal mol^−1^), *κ*, *k*_app_, *k*_r_, *k*_overall_ (M^−1^ s^−1^), and *Γ* (%) at 298.15 K, in the HO˙ + MB-intermediates in water

States	Mechanism		Δ*G*^≠^	*κ*	*k*_app_	*k*_r_	*Γ*	Products
I2	FHT	C16	8.9	4.4	2.40 × 10^7^	4.86 × 10^6^	0.1	1
		C17	9.0	4.6	2.10 × 10^7^	4.25 × 10^6^	0.1	2
		C19	6.4	5.1	1.10 × 10^9^	2.23 × 10^8^	2.4	3
		C20	5.7	4.9	2.00 × 10^9^	4.05 × 10^8^	4.3	4
	RAF	C1	5.8	1.3	3.60 × 10^8^	7.29 × 10^7^	0.8	5
		C3	5.6	1.2	4.50 × 10^8^	9.11 × 10^7^	1.0	6
		C4	2.5	1.0	2.60 × 10^9^	5.26 × 10^8^	5.6	7
		C8	2.7	1.1	2.50 × 10^9^	5.06 × 10^8^	5.4	8
		C10	3.7	1.1	2.00 × 10^9^	4.05 × 10^8^	4.3	9
		C14	5.0	1.1	9.40 × 10^8^	1.90 × 10^8^	2.0	10
	*k*_overall_ (*r*) (I2 + HO˙)					**2.43 × 10** ^**9**^	25.9	
I4		C19	6.5	1.1	3.30 × 10^8^	8.68 × 10^7^	0.9	11
		C20	6.2	12.3	2.10 × 10^9^	5.52 × 10^8^	5.9	12
	RAF	C1	7.1	1.2	4.50 × 10^7^	1.18 × 10^7^	0.1	13
		C2	4.1	1.1	1.80 × 10^9^	4.73 × 10^8^	5.0	7
		C8	1.8	1.0	2.30 × 10^9^	6.05 × 10^8^	6.4	14
		C14	7.2	1.2	4.10 × 10^7^	1.08 × 10^7^	0.1	15
	*k*_overall_ (*r*) (I4 + HO˙)					**1.74 × 10** ^**9**^	18.6	
I14	FHT	C16	8.6	2.6	2.50 × 10^7^	4.37 × 10^6^	0.1	17
		C19	7.5	5.4	2.70 × 10^8^	4.72 × 10^7^	0.5	18
		C20	7.2	5.1	4.30 × 10^8^	7.51 × 10^7^	0.8	19
	RAF	C1	7.2	1.1	3.90 × 10^7^	6.81 × 10^6^	0.1	20
		C2	6.0	1.2	2.70 × 10^8^	4.72 × 10^7^	0.5	10
		C3	4.5	1.1	1.40 × 10^9^	2.45 × 10^8^	2.6	21
		C4	5.0	1.1	8.80 × 10^8^	1.54 × 10^8^	1.6	16
		C5	6.9	1.2	6.30 × 10^7^	1.10 × 10^7^	0.1	22
		C8	3.3	1.1	2.20 × 10^9^	3.84 × 10^8^	4.1	23
		C10	5.4	1.0	5.70 × 10^8^	9.96 × 10^7^	1.1	24
		C11	6.6	1.2	9.80 × 10^7^	1.71 × 10^7^	0.2	25
	*k*_overall_ (*r*) (I14 + HO˙)					**1.09 × 10** ^**9**^	11.7	
I16	FHT	C19	5.6	16.0	5.80 × 10^9^	9.93 × 10^8^	5.4	26
		C20	5.5	9.5	1.70 × 10^9^	2.91 × 10^8^	5.0	27
	RAF	C2	5.6	1.2	2.60 × 10^7^	4.45 × 10^6^	0.9	1
		C4	6.9	1.2	2.81 × 10^8^	4.81 × 10^7^	0.1	28
		C8	1.5	1.1	2.14 × 10^9^	3.67 × 10^8^	4.4	29
		C10	5.2	1.0	8.70 × 10^8^	1.49 × 10^8^	1.4	30
		C16	3.6	1.0	1.98 × 10^9^	3.68 × 10^8^	3.9	31
	*k*_overall_ (*r*) (I16 + HO˙)					**1.97 × 10** ^**9**^	21.0	
I17	FHT	C19	5.8	19.0	5.80 × 10^9^	9.93 × 10^8^	10.6	32
		C20	6.4	10.5	1.70 × 10^9^	2.91 × 10^8^	3.1	33
	RAF	C2	7.4	1.2	2.60 × 10^7^	4.45 × 10^6^	0.1	2
		C4	5.9	1.0	2.81 × 10^8^	4.81 × 10^7^	0.5	34
		C8	3.1	1.0	2.14 × 10^9^	3.67 × 10^8^	3.9	35
		C10	5.0	1.0	8.70 × 10^8^	1.49 × 10^8^	1.6	36
		C17	4.1	1	6.20 × 10^9^	3.05 × 10^8^	3.3	37
	*k*_overall_ (*r*) (I17 + HO˙)					**2.16 × 10** ^**9**^	23.0	
*k*_overall_ (MB + HO˙, step 2)						**9.39 × 10** ^**9**^		

The results show that the intermediates exhibit high reactivity with the HO˙ radical, with an overall rate constant of the second step *k*_overall_ (MB + HO˙, step 2) = 9.39 × 10^9^ M^−1^ s^−1^. The I2 + ˙OH reaction has the highest rate, with *k*_overall_ (*r*) (I2 + HO˙) = 2.43 × 10^9^ M^−1^ s^−1^ (*Γ* = 25.9%). This reaction is 2.2 times faster than the I14 + ˙OH reaction (*k*_overall_ (*r*) (I14 + HO˙) = 1.09 × 10^9^ M^−1^ s^−1^, *Γ* = 11.7%). The I4/16/17 + ˙OH reactions have moderate activity with *k*_overall_ (*r*) values of 1.74 × 10^9^ (*Γ* = 18.6%), 1.97 × 10^9^ (*Γ* = 21.0%) and 2.16 × 10^9^ (*Γ* = 23.0%), respectively. The rate constant of the second step reaction (*k*_overall_ (MB + HO˙, step 2) = 9.39 × 10^9^ M^−1^ s^−1^) was approximately 1.1 times lower than the rate constant of the first step reaction (*k*_overall_ (MB + HO˙, step 1) = 1.02 × 10^10^ M^−1^ s^−1^, Table 1).

Fig. 3 illustrates that the two-step reaction between MB and ˙OH in water can potentially take place through five different pathways. The reaction can proceed initially *via* RAF(C2) followed by FHT(C20), or *via* RAF(C4/C8 or C10), resulting in the formation of cations 4 (4.3%), 7 (5.6%), 8 (5.4%) and 9 (4.3%). The RAF(C14)–RAF(C8) and RAF(C4)–RAF(C2/C14)/FHT(C20) processes produced the cations 23 (4.1%), 7 (5.0%), 14 (6.4%) and 12 (5.9%), respectively. Conversely, the FHT(C16/17)–FHT(C19/20)/RAF(C8/16/17) pathways could form the cations 26 (5.4%), 27 (5.0%), 29 (4.4%), 31 (3.9%), 32 (10.6%), 33 (3.1%), 35 (3.9%), and 37 (3.3%), respectively. Cation 32 has the largest branching ratio value, reaching 11.6%, while the other products displayed values below 7%. Overall the breakdown of MB by HO˙ radicals through a two-step reaction is intricate and could produce several compounds with low branching ratios (*Γ* < 11%).

The breakdown of MB by HO radicals leads to the formation of hydroxylated products (7, 8, 9, 14, and 23; *m*/*z* = 316) through the addition of two HO˙ groups to the MB molecule. This finding is consistent with prior experimental studies that used ESI-MS to analyze the breakdown of MB by HO˙ radicals.^10,11,28^ Our calculations also highlighted that the addition reaction can only occur in even positions (C2, C4, C8, C10, or C14), but not in odd positions such as the C1 and C11 positions (Fig. 1) as shown in previous studies.^10,11,28^ Hence, the computational approach uncovered details of the MB breakdown process that experimental observations failed to reveal.

### 3.2. Environmental risk assessment

#### 3.2.1. Environmental lifetimes

**Fig. 4:**
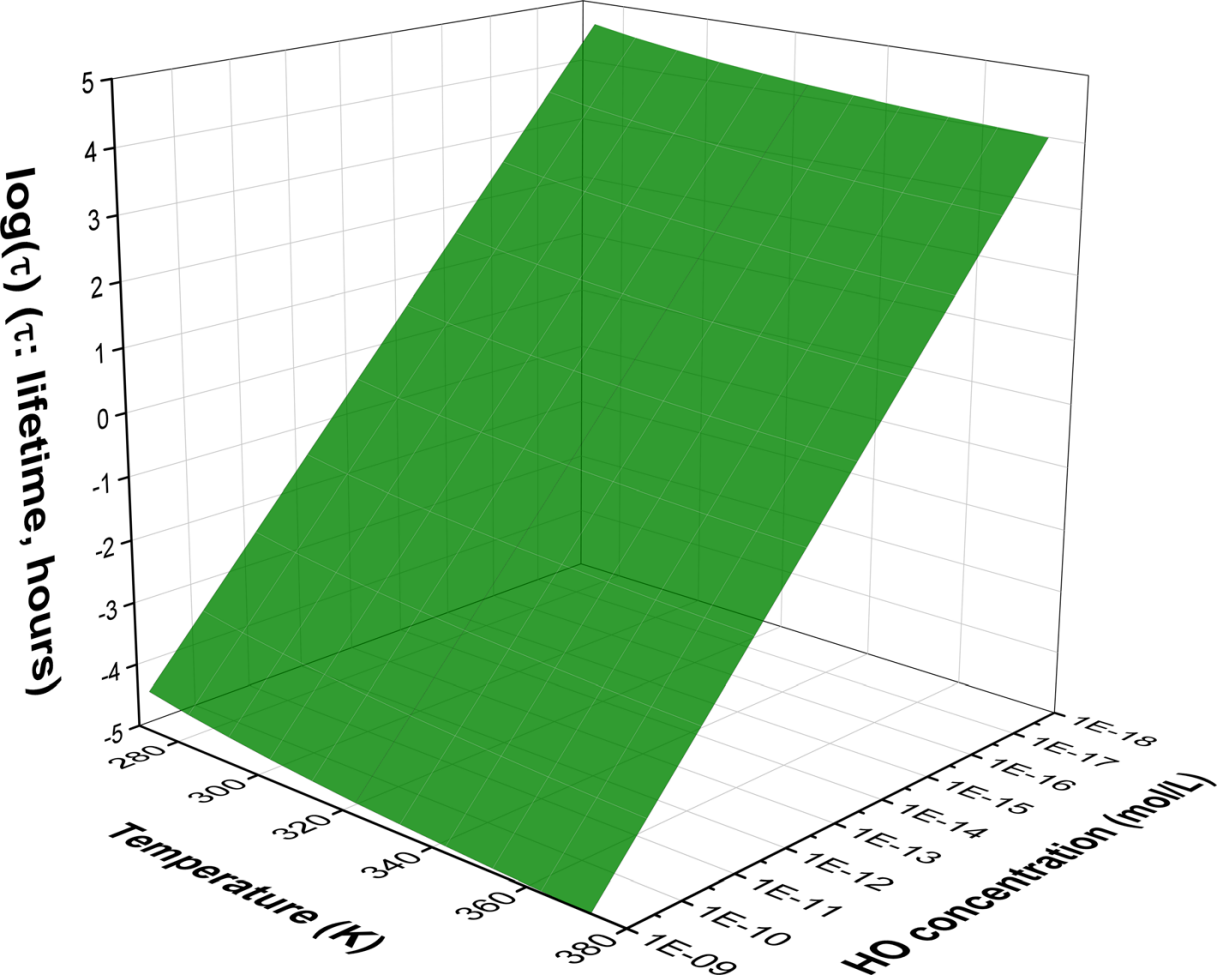
Lifetime (log(*τ*), hours) of MB in water at pH > 2.0 at 273–373 K.

The attribute of environmental persistence of a compound is highly significant for its environmental safety as a lack of breakdown processes means a compound retains its toxicity allowing its transportation to distant regions.^72^ In the presence of HO˙ radicals in water at a temperature range of 273–373 K, with environmentally relevant pH values (pH > 2.0, MB > 99%), and with [HO˙] = 10^−18^ to 10^−15^ M in natural water^25–27^ and 10^−10^ to 10^−9^ M in AOP-treated wastewater,^76^ the lifetime of MB was calculated (Fig. 4 and Table S2, ESI†). In water, MB degrades within 5.17 × 10^−5^ to 5.04 × 10^4^ hours in the range of [HO˙] = 10^−18^ to 10^−9^ M (Table S2, ESI†). Specifically, the degradation of MB in the AOP-treated wastewater can occur within a second (0.04–1.82 s), whereas in natural water, it can occur in 11.66 to 5.04 × 10^4^ hours (log(*τ*) = 1.07–4.70) (*i.e.* 11.66 hours to 5.76 years) in the 273–373 K temperature range. For a given [HO˙] concentration, the value of *τ* decreases as the temperature increases. Thus, the lifetime of ˙OH-degraded MB in natural water environments is estimated to be between 50.4 and 5.04 × 10^4^ hours (log(*τ*) = 1.70–4.70) at a low temperature, which decreases to 11.66–1.17 × 10^4^ hours (log(*τ*) = 1.07–4.07) at 373 K.

#### 3.2.2. Ecological toxicity, developmental toxicity and mutagenicity

**Fig. 5:**
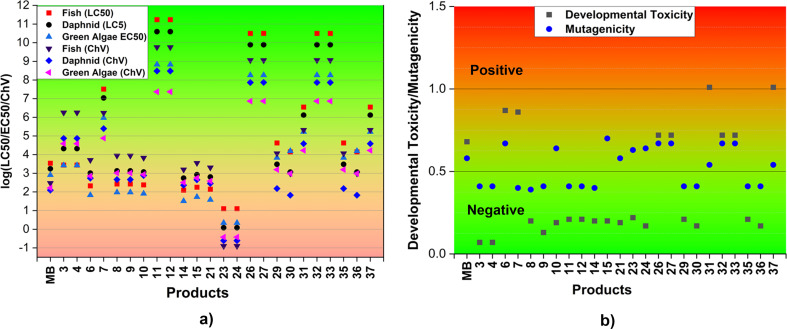
Acute and chronic toxicity (log(LC_50_/EC_50_/ChV), mg L^−1^) (a) and the developmental toxicity and mutagenicity (b) of MB and the main products (F: fish; D: daphnia; GA: green algae).

To evaluate the environmental effects of MB and its breakdown products, we analyze the acute and chronic toxicity towards three aquatic organisms: fish, daphnia, and green algae. The estimation is based on the reaction outcomes and the ECOSAR program. Fig. 5a and Table S3, ESI† depict the results. Previous research has shown that substances with LC_50_/EC_50_/ChV (mg L^−1^) values lower than 100 (log(LC_50_/EC_50_/ChV) ≤ 2) are harmful to green algae, fish, and daphnia.^67,77^ The log values for LC_50_/EC_50_/ChV of all the species examined for products 23 and 24 are less than 2. This result indicates that the chemicals demonstrate toxicity against three distinct groups of aquatic organisms. The products 3, 4, 7, 11, 12, 26, 27, 29, 31, 32, 33, 35, and 37, as well as MB, might not cause damage to fish, daphnia, or green algae. The degradation products 6, 8, 9, 10, 14, 15 and 21 may be harmful with green algae (EC_50_(green algae) < 2), whereas the products 30 and 36 will be hazardous with daphnid (ChV(daphnid) < 2). Therefore, it is crucial to consider the possible ecological consequences linked to the degradation process of MB.

In order to assess the impact of MB and its breakdown products on living creatures, the developmental toxicity and mutagenicity of these compounds were also determined using T.E.S.T. The corresponding outcomes are illustrated in Fig. 5b and Table S3, ESI.† The results imply that products 6, 7, 26, 27, 31, 32, 33, and 37, along with MB may have developmental toxicity, as indicated by toxicity values for development that are higher than 0.5. These substances have the ability to disrupt the processes of nucleic acid translation and expression, potentially affecting the growth and development of humans.^71^ Simultaneously, the calculations indicate that the products 6, 10, 15, 21, 23, 24, 26, 27, 31, 32, 33, and 37, as well as MB, may present mutagenic hazards (mutagenicity values ≥ 0.5). The rest of the products, specifically 3, 4, 8, 9, 11, 12, 14, 29, 30, 35, and 36, do not exhibit any developmental toxicity or mutagenicity. This can be explained by the structural change where the addition reaction has the potential to disrupt the aromatic ring of MB which is mostly responsible for its toxicity.

#### 3.2.3. Bioconcentration, biodegradability

The level of harm caused to organisms is directly related to the amount of exposure they have to reactants and the substances produced when those reactants break down. Estimating bioaccumulation serves as a rational method for measuring the degree of exposure to a chemical.^78^MB builds up in organisms through various processes, leading to negative health impacts and an increase in ecological toxicity. Bioconcentration factors (BCFs) of MB and its transformation products can be estimated using the BCFBAF module of EPISUITE. Typically, the accumulation of substances in fish tissues increases as the bioconcentration factor (BCF) of the organic matter increases. This is seen in Table S3, ESI.† A chemical is deemed to possess substantial bioaccumulation potential if its bioconcentration factor (BCF) exceeds 5000.^79^ The results indicate that the interaction between the degradation products of MB and the HO˙ radical in a two-step reaction does not have an effect on the BCF values. Consequently, the BCF values of the products closely resembled that of the initial material (3.162). Hence, the likelihood of organisms accumulating MB and its breakdown products may not be substantial.

The biodegradability of the reactant MB and its degradation products was assessed using the BIOWIN 3, 4, and 5 models incorporated in the EPISUITE program (Table S3, ESI†).^72^ The results obtained from the BIOWIN models suggest that degradation products undergo initial biodegradation during a period ranging from a few days to months, while both the MB and degradation products may not be biodegradable. Consequently, the breakdown of MB by HO˙ radicals in the two-step process could result in the formation of non-biodegradable substances. However, these substances may not accumulate in living organisms.

## 4. Conclusion

A computational analysis was conducted to study the breakdown pathways of MB in the presence of HO˙. An assessment of the thermodynamic and kinetic parameters of possible reaction paths was conducted in order to ascertain the most probable products. It was found that in natural water bodies MB can undergo degradation with lifetimes in the broad range of 11.66 to 5.04 × 10^4^ hours (*i.e.* 11.66 hours to 5.76 years). The HO˙ + MB reaction in water can take place *via* either FHT or RAF mechanism. The addition reaction is possible at carbon atoms at most even positions C2, C4, C8, C10, or C14, but not at odd positions. The intermediates and some of the products generated in the process can pose a threat to aquatic organisms, including fish, daphnia, and green algae. Additionally, they have the potential to induce developmental toxicity and have poor biodegradability. However, the introduction of the HO radical to the aromatic ring might lead to the creation of compounds that do not exhibit any developmental toxicity or mutagenicity. Hence, MB poses a moderate environmental risk with a mix of safe and toxic breakdown products.

## Data availability

The data supporting this article have been included as part of the ESI.†

## Conflicts of interest

There are no conflicts to declare.

## Supplementary Material

RA-014-D4RA05437G-s001
