# Supplementary material for: Reaction of methylene blue with OH radicals in the aqueous environment: mechanism, kinetics, products and risk assessment
Source: RSC Adv. 2024 Aug 27;14(37):27265–73. doi: 10.1039/d4ra05437g (PMC11348493; doi:10.1039/d4ra05437g)
Supplement: RA-014-D4RA05437G-s001 [file RA-014-D4RA05437G-s001.pdf]

Supplementary Information (SI) for RSC Advances.
This journal is © The Royal Society of Chemistry 2024

Supporting Information (SI)

Reaction of methylene blue with OH radicals in the aqueous environment: mechanism, kinetics, products and risk assessment

Quan V. Vo[1*], Luu Thi Thu Thao,[2] Tran Duc Manh,[2] Mai Van Bay,[2] Truong Le Bich Tram[3], Nguyen Thi Hoa[1] and Adam Mechler[4]

[1]*The University of Danang - University of Technology and Education, Danang 550000, Vietnam.*
[2]*The University of Danang - University of Sciences and Education, Danang 550000, Vietnam.*
[3]*Department of Science and International Cooperation, The University of Danang, Danang 550000, Vietnam*
[4]*Department of Biochemistry and Chemistry, La Trobe University, Victoria 3086, Australia.*
**Corresponding author: vvquan@ute.udn.vn;*

Table of Contents

Figure S1. The acid dissociation equilibrium (a) and molar fractions (b) of MB S2

Table S1. Calculated $\Delta G^{\neq}$ (kcal/mol), tunneling corrections (κ), rate constants (k_{app}, k_r, and $k_{overall}$ (r) $M^{-1}\ s^{-1}$), and branching ratios (Γ, %) at 298.15 K, in all of the reactions of the MB intermediates with $HO^{\bullet}$ in water S3

Table S2. Lifetime (τ) in water at pH > 2.0 at 273–373 K, [$HO^{\bullet}$] = 10^{-18} - 10^{-9} M S6

Table S3. Developmental toxicity, mutagenicity, Bioconcentration (BCF) and biodegradability of MB and the main degradation products. S7

Table S4: The Cartesian coordinates and energies of TSs in water S9

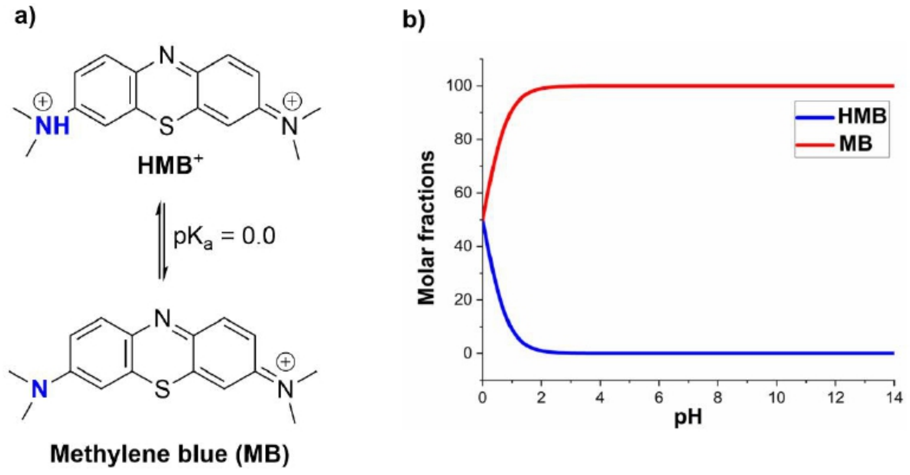

Figure S1. The acid dissociation equilibrium (a) and molar fractions (b) of MB

Table S1. Calculated ΔG≠ (kcal/mol), tunneling corrections (κ), rate constants (k_{app}, k_r, and $k_{overall}$ (r) M^{-1} s^{-1}), and branching ratios (Γ, %) at 298.15 K, in all of the reactions of the MB intermediates with HO• in water

**States**	Mechanism		ΔG≠	κ	k_D	k_{app}	r	k_r	Γ
**I2**	SET		18.0	2.6	8.50E+09	4.00E-01	0.202	7.90E-10	0.0
	FHT	C16	8.9	4.4	2.90E+09	2.40E+07	0.202	4.86E+06	0.1
		C17	9.0	4.6	2.90E+09	2.10E+07	0.202	4.25E+06	0.1
		C19	6.4	5.1	3.00E+09	1.10E+09	0.202	2.23E+08	**2.4**
		C20	5.7	4.9	3.00E+09	2.00E+09	0.202	4.05E+08	**4.3**
	RAF	C1	5.8	1.3	2.50E+09	3.60E+08	0.202	7.29E+07	0.8
		C2					0.202	0.00E+00	0.0
		C3	5.6	1.2	1.80E+09	4.50E+08	0.202	9.11E+07	**1.0**
		C4	2.5	1.0	2.60E+09	2.60E+09	0.202	5.26E+08	**5.6**
		C5	8.5	1.1	2.30E+09	4.50E+06	0.202	9.11E+05	0.0
		C7	12.8	1.3	2.10E+09	3.50E+03	0.202	7.09E+02	0.0
		C8	2.7	1.1	2.50E+09	2.50E+09	0.202	5.06E+08	**5.4**
		C9	19.1	1.3	2.10E+09	8.70E-02	0.202	1.76E-02	0.0
		C10	3.7	1.1	2.50E+09	2.00E+09	0.202	4.05E+08	**4.3**
		C11	9.7	1.2	2.20E+09	5.60E+05	0.202	1.13E+05	0.0
		C12	8.7	1.1	2.30E+09	2.90E+06	0.202	5.87E+05	0.0
		C14	5.0	1.1	2.40E+09	9.40E+08	0.202	1.90E+08	**2.0**
		N13	23.3	53.5	1.90E+09	2.00E-03	0.202	4.05E-04	0.0
								**2.43E+09**	25.9
**I4**	SET		29.2	2.7	8.70E+09	2.50E-09	0.263	6.57E-10	0.0
	FHT	C16	10.0	6.3	2.80E+09	5.30E+06	0.263	1.39E+06	0.0
		C17	10.6	7.2	2.80E+09	2.50E+06	0.263	6.57E+05	0.0
		C19	6.5	1.1	3.10E+09	3.30E+08	0.263	8.68E+07	**0.9**
		C20	6.2	12.3	3.00E+09	2.10E+09	0.263	5.52E+08	**5.9**
	RAF	C1	7.1	1.2	2.30E+09	4.50E+07	0.263	1.18E+07	0.1
		C2	4.1	1.1	2.40E+09	1.80E+09	0.263	4.73E+08	**5.0**
		C3	14.9	1.6	2.10E+09	1.30E+02	0.263	3.42E+01	0.0
		C4					0.263	0.00E+00	0.0
		C5	8.7	1.1	2.50E+09	2.75E+06	0.263	7.22E+05	0.0
		C7	14.2	1.3	2.10E+09	3.10E+02	0.263	8.15E+01	0.0
		C8	1.8	1.0	2.30E+09	2.30E+09	0.263	6.05E+08	**6.4**

		C9	13.9	1.2	2.10E+09	5.10E+02	0.263	1.34E+02	0.0
		C10	21.0	1.2	2.30E+09	3.10E-03	0.263	8.15E-04	0.0
		C11	23.9	1.2	2.20E+09	2.20E-05	0.263	5.78E-06	0.0
		C12	8.9	1.1	2.20E+09	2.10E+06	0.263	5.52E+05	0.0
		C14	7.2	1.2	2.40E+09	4.10E+07	0.263	1.08E+07	0.1
		N13	22.8	66.1	1.90E+09	7.40E-03	0.263	1.95E-03	0.0
	Total							**1.74E+09**	18.6
**I14**	SET		9.5	4.0	8.70E+09	4.10E+02	0.175	7.16E+01	0.0
	FHT	C16	8.6	2.6	2.80E+09	2.50E+07	0.175	4.37E+06	0.1
		C17	9.1	4.4	2.80E+09	1.80E+07	0.175	3.15E+06	0.0
		C19	7.5	5.4	2.80E+09	2.70E+08	0.175	4.72E+07	0.5
		C20	7.2	5.1	2.90E+09	4.30E+08	0.175	7.51E+07	0.8
	RAF	C1	7.2	1.1	2.30E+09	3.90E+07	0.175	6.81E+06	0.1
		C2	6.0	1.2	2.30E+09	2.70E+08	0.175	4.72E+07	0.5
		C3	4.5	1.1	2.40E+09	1.40E+09	0.175	2.45E+08	**2.6**
		C4	5.0	1.1	2.40E+09	8.80E+08	0.175	1.54E+08	**1.6**
		C5	6.9	1.2	2.30E+09	6.30E+07	0.175	1.10E+07	0.1
		C7	11.0	1.3	2.10E+09	7.30E+04	0.175	1.28E+04	0.0
		C8	3.3	1.1	2.40E+09	2.20E+09	0.175	3.84E+08	**4.1**
		C9	24.9	1.4	2.00E+09	4.60E-06	0.175	8.04E-07	0.0
		C10	5.4	1.0	2.30E+09	5.70E+08	0.175	9.96E+07	**1.1**
		C11	6.6	1.2	2.20E+09	9.80E+07	0.175	1.71E+07	0.2
		C12	14.6	1.2	2.00E+09	1.40E+02	0.175	2.45E+01	0.0
		N13	10.9	1.0	2.30E+09	6.70E+04	0.175	1.17E+04	0.0
								**1.09E+09**	11.7
**I16**	SET		59.8	1.6	8.60E+09	9.10E-32	0.186	1.69E-32	0.0
	FHT	C16					0.186	0.00E+00	0.0
		C17	15.5	8.4	2.80E+09	6.50E+02	0.186	1.21E+02	0.0
		C19	5.6	16.0	3.00E+09	2.70E+09	0.186	5.03E+08	**5.4**
		C20	5.5	9.5	3.00E+09	2.50E+09	0.186	4.65E+08	**5.0**
	RAF	C1	10.5	1.2	2.20E+09	1.60E+05	0.186	2.98E+04	0.0
		C2	5.6	1.2	2.30E+09	4.40E+08	0.186	8.19E+07	0.9
		C3	14.1	1.2	2.10E+09	3.50E+02	0.186	6.52E+01	0.0
		C4	6.9	1.2	2.30E+09	6.10E+07	0.186	1.14E+07	0.1
		C5	13.5	1.2	2.20E+09	1.00E+03	0.186	1.86E+02	0.0
		C7	11.8	1.3	2.10E+09	1.80E+04	0.186	3.35E+03	0.0

		C8	1.5	1.1	2.20E+09	2.20E+09	0.186	4.10E+08	**4.4**
		C9	16.5	1.3	2.00E+09	6.90E+00	0.186	1.28E+00	0.0
		C10	5.2	1.0	2.30E+09	6.97E+08	0.186	1.30E+08	**1.4**
		C11	8.5	1.2	2.20E+09	4.40E+06	0.186	8.19E+05	0.0
		C12	11.3	1.2	2.10E+09	3.80E+04	0.186	7.07E+03	0.0
		C14	10.6	1.2	2.20E+09	1.30E+05	0.186	2.42E+04	0.0
		C16	3.6	1	2.30E+09	1.40E+10	0.186	3.68E+08	3.9
		N13	29.0	97.5	1.90E+09	3.10E-07	0.186	5.77E-08	0.0
								**1.97E+09**	21.0
**I17**	SET		107.2	0.9	8.70E+09	1.70E-66	0.171	2.91E-67	0.0
	FHT	C16	16.5	11.7	2.80E+09	1.70E+02	0.171	2.91E+01	0.0
		C17					0.171	0.00E+00	0.0
		C19	5.8	19.0	8.20E+09	5.80E+09	0.171	9.93E+08	**10.6**
		C20	6.4	10.5	3.00E+09	1.70E+09	0.171	2.91E+08	**3.1**
	RAF	C1	10.1	1.2	2.20E+09	3.20E+05	0.171	5.48E+04	0.0
		C2	7.4	1.2	2.30E+09	2.60E+07	0.171	4.45E+06	0.1
		C3	15.5	1.2	2.10E+09	3.00E+01	0.171	5.14E+00	0.0
		C4	5.9	1.0	2.30E+09	2.81E+08	0.171	4.81E+07	0.5
		C5	13.9	1.2	2.20E+09	5.10E+02	0.171	8.73E+01	0.0
		C7	12.5	1.3	2.10E+09	5.50E+03	0.171	9.42E+02	0.0
		C8	3.1	1.0	2.30E+09	2.14E+09	0.171	3.67E+08	**3.9**
		C9	16.4	1.3	2.00E+09	7.50E+00	0.171	1.28E+00	0.0
		C10	5.0	1.0	2.30E+09	8.70E+08	0.171	1.49E+08	**1.6**
		C11	26.2	1.2	2.10E+09	4.90E-07	0.171	8.39E-08	0.0
		C12	11.5	1.2	2.10E+09	2.90E+04	0.171	4.97E+03	0.0
		C14	10.7	1.2	2.20E+09	1.10E+05	0.171	1.88E+04	0.0
		C17	4.1	1	2.50E+09	6.20E+09	0.171	3.05E+08	**3.3**
		N13	28.7	84.2	1.80E+09	4.90E-07	0.171	8.39E-08	0.0
								**2.16E+09**	23.0
								**9.39E+09**	

Table S2. Lifetime (τ) in water at pH > 2.0 at 273–373 K, $[HO^{\bullet}] = 10^{-18}$ - 10^{-9} M

Lifetime (τ, hours)		[OH] (mol/L)									
		1.00E-09	1.00E-10	1.00E-11	1.00E-12	1.00E-13	1.00E-14	1.00E-15	1.00E-16	1.00E-17	1.00E-18
**Tem. (K)**	273	5.04E-05	5.04E-04	5.04E-03	5.04E-02	5.04E-01	5.04E+00	5.04E+01	5.04E+02	5.04E+03	5.04E+04
	283	3.82E-05	3.82E-04	3.82E-03	3.82E-02	3.82E-01	3.82E+00	3.82E+01	3.82E+02	3.82E+03	3.82E+04
	293	3.05E-05	3.05E-04	3.05E-03	3.05E-02	3.05E-01	3.05E+00	3.05E+01	3.05E+02	3.05E+03	3.05E+04
	303	2.50E-05	2.50E-04	2.50E-03	2.50E-02	2.50E-01	2.50E+00	2.50E+01	2.50E+02	2.50E+03	2.50E+04
	313	2.13E-05	2.13E-04	2.13E-03	2.13E-02	2.13E-01	2.13E+00	2.13E+01	2.13E+02	2.13E+03	2.13E+04
	323	1.84E-05	1.84E-04	1.84E-03	1.84E-02	1.84E-01	1.84E+00	1.84E+01	1.84E+02	1.84E+03	1.84E+04
	333	1.64E-05	1.64E-04	1.64E-03	1.64E-02	1.64E-01	1.64E+00	1.64E+01	1.64E+02	1.64E+03	1.64E+04
	343	1.47E-05	1.47E-04	1.47E-03	1.47E-02	1.47E-01	1.47E+00	1.47E+01	1.47E+02	1.47E+03	1.47E+04
	353	1.34E-05	1.34E-04	1.34E-03	1.34E-02	1.34E-01	1.34E+00	1.34E+01	1.34E+02	1.34E+03	1.34E+04
	363	1.24E-05	1.24E-04	1.24E-03	1.24E-02	1.24E-01	1.24E+00	1.24E+01	1.24E+02	1.24E+03	1.24E+04
	373	1.17E-05	1.17E-04	1.17E-03	1.17E-02	1.17E-01	1.17E+00	1.17E+01	1.17E+02	1.17E+03	1.17E+04
$\tau = 1/([HO].k_{overall})$											

Table S3. Developmental toxicity, mutagenicity, Bioconcentration (BCF) and biodegradability of MB and the main degradation products.

Products	T.E.S.T		BCF	Biodegradability			
	Developmental Toxicity	Mutagenicity		BIOWIN3	BIOWIN4	BIOWIN5	Biodegradable
**MB**	0.68	0.58	**3.162**	1.9827 (months)	2.8103 (weeks)	-0.3581	NO
**3**	0.07	0.41	**3.162**	2.6931 (weeks-months)	3.5423 (days-weeks)	0.0872	NO
**4**	0.07	0.41	**3.162**	2.5581 (weeks-months)	3.4339 (days-weeks)	-0.0841	NO
**6**	0.87	0.67	**3.162**	2.1886 (months)	3.0763 (weeks)	-0.0493	NO
**7**	0.86	0.40	**3.162**	2.8132 (weeks)	3.6458 (days-weeks)	0.1459	NO
**8**	0.20	0.39	**3.162**	2.5495 (weeks-months)	3.3520 (days-weeks)	0.0813	NO
**9**	0.13	0.41	**3.162**	2.5607 (weeks-months)	3.3592 (days-weeks)	0.0505	NO
**10**	0.19	0.64	**3.162**	2.4411 (weeks-months)	3.3629 (days-weeks)	0.0461	NO
**11**	0.21	0.41	**3.162**	2.5581 (weeks-months)	3.4339 (days-weeks)	-0.0902	NO
**12**	0.21	0.41	**3.162**	2.5581 (weeks-months)	3.4339 (days-weeks)	-0.0902	NO
**14**	0.20	0.40	**3.162**	2.3221 (weeks-months)	3.1611 (weeks)	-0.2187	NO
**15**	0.20	0.70	**3.162**	2.0492 (months)	2.9651 (weeks)	-0.2006	NO
**21**	0.19	0.58	**3.162**	1.8165 (months)	2.7935 (weeks)	-0.1490	NO

**23**	0.22	0.63	**3.162**	2.1886 (months	3.0763 (weeks)	-0.0555	NO
**24**	0.17	0.64	**3.162**	2.1886 (months)	3.0763 (weeks)	-0.0555	NO
**26**	0.72	0.67	**3.162**	2.5729 (weeks-months)	3.4389 (days-weeks)	0.0213	NO
**27**	0.72	0.67	**3.162**	2.5729 (weeks-months)	3.4389 (days-weeks)	0.0213	NO
**29**	0.21	0.41	**3.162**	2.3369 (weeks-months)	3.1660 (weeks)	-0.1071	NO
**30**	0.17	0.41	**3.162**	2.3369 (weeks-months)	3.1660 (weeks)	-0.1071	NO
**31**	1.01	0.54	**3.162**	2.4405 (weeks-months)	3.2558 (days-weeks)	-0.0144	NO
**32**	0.72	0.67	**3.162**	2.5729 (weeks-months)	3.4389 (days-weeks)	0.0213	NO
**33**	0.72	0.67	**3.162**	2.5729 (weeks-months)	3.4389 (days-weeks)	0.0213	NO
**35**	0.21	0.41	**3.162**	2.3369 (weeks-months)	3.1660 (weeks)	-0.1071	NO
**36**	0.17	0.41	**3.162**	2.3369 (weeks-months)	3.1660 (weeks)	-0.1071	NO
**37**	1.01	0.54	**3.162**	2.4405 (weeks-months)	3.2558 (days-weeks)	-0.0144	NO

Table S4: The Cartesian coordinates and energies of TSs in water

**Name**	**MB-C1-OH-RAF**
Cartesian Coordinates	Frequency and Energy
C 3.48515200 1.16388100 -0.38835400	Zero-point correction= 0.326537 (Hartree/Particle)
C 3.61825400 -0.25406600 -0.17965500	Thermal correction to Energy= 0.347356
C 2.43776900 -1.02070600 0.01149000	Thermal correction to Enthalpy= 0.348300
C 2.24284800 1.76840700 -0.32378900	Thermal correction to Gibbs Free Energy= 0.276250
C 1.19709800 -0.42265700 -0.01898100	Sum of electronic and zero-point Energies= -1258.090897
S -0.19273300 -1.44308300 0.18749500	Sum of electronic and thermal Energies= -1258.070078
N -0.11058200 1.64065800 -0.30557800	Sum of electronic and thermal Enthalpies= -1258.069134
C 1.03550700 0.98901000 -0.22319400	Sum of electronic and thermal Free Energies= -1258.141185
C -1.53227600 -0.34243600 0.00957900	
C -2.80163600 -0.86698800 0.09623000	
C -3.93777300 -0.02540200 -0.03849800	
C -3.72180900 1.37959400 -0.24966200	
C -2.46165700 1.87977200 -0.33185700	
C -1.30671600 1.05259200 -0.21128400	
N -5.17839200 -0.52453900 0.02680100	
C -5.38702900 -1.95777800 0.21744200	
C -6.34300000 0.35111300 -0.10150200	
N 4.82590700 -0.82115100 -0.17847200	
C 6.03731700 -0.02252800 -0.38565700	
C 4.95716200 -2.26410300 0.01714700	
H 4.35714100 1.77651500 -0.56310400	
H 2.13530200 2.81052100 -0.59263900	
H 2.50745600 -2.08895300 0.16523900	
H -2.92687000 -1.92826000 0.26473200	
H -4.56519300 2.04773300 -0.34837100	
H -2.29876300 2.93869400 -0.49478300	
H -6.45369600 -2.16015100 0.20731300	
H -4.91388900 -2.52734200 -0.58563900	
H -4.97579200 -2.28757100 1.17458200	
H -6.34381900 1.11448800 0.67939700	
H -6.35822200 0.84042800 -1.07779400	
H -7.24053900 -0.25107600 0.00126700	
H 6.13152400 0.74235100 0.38706000	
H 6.89710600 -0.68280900 -0.33109100	
H 6.02275300 0.45522900 -1.36679100	
H 4.44235500 -2.80772200 -0.77821200	
H 6.01029500 -2.52525700 -0.00148000	
H 4.53591300 -2.55939200 0.98043600	
O 2.69102500 2.28459100 1.63279200	
H 2.71868000 1.39415300 2.02163400	
**Name**	**MB-C2-OH-RAF**
Cartesian Coordinates	Frequency and Energy
C -3.41209600 -1.23534200 -0.30826100	Zero-point correction= 0.325650 (Hartree/Particle)
C -3.55614000 0.20191600 -0.22572000	Thermal correction to Energy= 0.346794
C -2.37903100 0.98621400 -0.09556200	Thermal correction to Enthalpy= 0.347738
C -2.15919200 -1.79933600 -0.38118700	Thermal correction to Gibbs Free Energy= 0.274859
C -1.14123100 0.39001400 -0.10534800	Sum of electronic and zero-point Energies= -1258.096887

S 0.24651100 1.42350900 0.08346700<br>N 0.18955400 -1.68385200 -0.30670300<br>C -0.98142900 -1.03037200 -0.25880600<br>C 1.58751300 0.32031700 -0.02505900<br>C 2.85133600 0.84153000 0.07059300<br>C 3.99298000 -0.00855800 -0.00449200<br>C 3.78430200 -1.42460600 -0.18680000<br>C 2.53189700 -1.92545300 -0.27998000<br>C 1.36468900 -1.09272500 -0.20607500<br>N 5.22384300 0.48851800 0.09036900<br>C 5.43158200 1.92217800 0.30073400<br>C 6.39687800 -0.38379100 -0.01567500<br>N -4.76347200 0.76075900 -0.25098900<br>C -5.96910200 -0.06419500 -0.35427700<br>C -4.90978100 2.21219000 -0.14058400<br>H -4.27852000 -1.85221100 -0.49280400<br>H -2.05222300 -2.87025600 -0.50735300<br>H -2.45319100 2.06054900 0.00656500<br>H 2.97866000 1.90814500 0.19858900<br>H 4.63178400 -2.09231900 -0.24335800<br>H 2.36904400 -2.98824100 -0.41419300<br>H 6.49656200 2.11063000 0.39154300<br>H 5.03988800 2.49521300 -0.54223000<br>H 4.93570700 2.24889500 1.21663100<br>H 6.40637000 -1.11741500 0.79267900<br>H 6.40280000 -0.90451200 -0.97442600<br>H 7.28968500 0.22913400 0.05556700<br>H -6.01898700 -0.77544100 0.47176700<br>H -6.83615000 0.58744200 -0.31026000<br>H -5.98598000 -0.60900600 -1.30011100<br>H -4.38356300 2.71487800 -0.95429800<br>H -5.96485400 2.46021600 -0.20003200<br>H -4.51523500 2.56783900 0.81369100<br>O -3.74318000 -1.39564500 1.90498200<br>H -3.20905200 -2.20237900 1.98695300	Sum of electronic and thermal Energies= -1258.075743<br>Sum of electronic and thermal Enthalpies= -1258.074799<br>Sum of electronic and thermal Free Energies= -1258.147677
**Name**	**MB-C3-OH-RAF**
Cartesian Coordinates	Frequency and Energy
C 3.38461800 1.47602700 -0.10772600<br>C 3.59329400 0.03012400 -0.12697200<br>C 2.41005400 -0.80435200 -0.08300400<br>C 2.14118600 1.98816800 -0.09124100<br>C 1.14763100 -0.25270900 -0.07161500<br>S -0.20487500 -1.35093700 0.00705900<br>N -0.21437100 1.78664000 -0.07253200<br>C 0.96017600 1.15789500 -0.08309400<br>C -1.57381100 -0.27977600 -0.02810100<br>C -2.82661500 -0.83474900 -0.01718200<br>C -3.98478000 -0.00598400 -0.02811700<br>C -3.80435500 1.42574700 -0.04630200<br>C -2.56171700 1.96190500 -0.05949800<br>C -1.38256200 1.15161500 -0.05402800<br>N -5.20713300 -0.53130500 -0.02247400<br>C -5.39187400 -1.98356600 -0.01024000<br>C -6.39744400 0.32457000 -0.03104800	Zero-point correction= 0.326219 (Hartree/Particle)<br>Thermal correction to Energy= 0.347206<br>Thermal correction to Enthalpy= 0.348150<br>Thermal correction to Gibbs Free Energy= 0.275987<br>Sum of electronic and zero-point Energies= -1258.090549<br>Sum of electronic and thermal Energies= -1258.069562<br>Sum of electronic and thermal Enthalpies= -1258.068618<br>Sum of electronic and thermal Free Energies= -1258.140781

N	4.77214000	-0.46785300	-0.52230600		
C	5.97738500	0.34956900	-0.34573200		
C	4.98081300	-1.91647200	-0.50280200		
H	4.23972600	2.13607200	-0.10506500		
H	1.98252400	3.05965700	-0.08218900		
H	2.52544100	-1.87939600	-0.06875700		
H	-2.93230200	-1.91103600	0.00148100		
H	-4.66688000	2.07670800	-0.05155900		
H	-2.42148600	3.03578200	-0.07615700		
H	-6.45543200	-2.19871600	-0.01614300		
H	-4.93293500	-2.43373500	-0.89240200		
H	-4.94585400	-2.41785100	0.88647800		
H	-6.41651500	0.96537900	0.85181300		
H	-6.41863800	0.94464100	-0.92856200		
H	-7.27621900	-0.31187600	-0.02228900		
H	6.11294800	0.61659000	0.70605900		
H	6.83386000	-0.23004700	-0.67814100		
H	5.92735000	1.25378500	-0.94938300		
H	4.23066200	-2.42156200	-1.10850900		
H	5.95637100	-2.12871700	-0.93171700		
H	4.94617800	-2.30093900	0.52092900		
O	3.57601900	-0.34235900	1.88210600		
H	2.89461300	0.25867600	2.21571800		
**Name**				**MB-C4-OH-RAF**	
Cartesian Coordinates				Frequency and Energy	
C	3.42302500	1.52457000	-0.11728200	Zero-point correction=	0.325802 (Hartree/Particle)
C	3.62280200	0.10393500	-0.23401100	Thermal correction to Energy=	0.346932
C	2.47320700	-0.74107900	-0.21656400	Thermal correction to Enthalpy=	0.347876
C	2.17144500	2.03825800	-0.03283700	Thermal correction to Gibbs Free Energy=	0.274854
C	1.19975900	-0.18970900	-0.17944100	Sum of electronic and zero-point Energies=	-1258.100370
S	-0.14253800	-1.28290600	-0.23899600	Sum of electronic and thermal Energies=	-1258.079241
N	-0.18349600	1.82920900	0.02019700	Sum of electronic and thermal Enthalpies=	-1258.078297
C	0.99979900	1.21637700	-0.06234500	Sum of electronic and thermal Free Energies=	-1258.151318
C	-1.52109300	-0.23144800	-0.11842100		
C	-2.76937500	-0.80488800	-0.14044900		
C	-3.93500700	0.00259400	-0.03747600		
C	-3.77090800	1.42994800	0.08038000		
C	-2.53473900	1.98178500	0.09745800		
C	-1.34432700	1.19140200	-0.00019600		
N	-5.15294500	-0.54180800	-0.04809000		
C	-5.31480300	-1.99064500	-0.16409800		
C	-6.35015700	0.29475900	0.05842100		
N	4.84825500	-0.40981500	-0.33027600		
C	6.02779700	0.45735300	-0.30733500		
C	5.03892900	-1.85862700	-0.37217000		
H	4.27395000	2.18996000	-0.09596200		
H	2.02056600	3.10724000	0.06134800		
H	2.58202600	-1.80879300	-0.34651900		
H	-2.86104300	-1.87854000	-0.23330300		
H	-4.64041400	2.06705600	0.15517500		
H	-2.40838600	3.05428900	0.18572000		
H	-6.37447300	-2.22516200	-0.14821300		
H	-4.88763400	-2.35308300	-1.10154500		
H	-4.82812900	-2.50117300	0.66963500		

H	-6.35171900	0.85212900	0.99689400	
H	-6.40825700	0.99678100	-0.77553900	
H	-7.22329400	-0.34969000	0.03357500	
H	6.10115700	0.98835500	0.64418400	
H	6.91090300	-0.16071700	-0.43483800	
H	5.98887500	1.18296500	-1.12132100	
H	4.57525200	-2.28452000	-1.26440800	
H	6.10345400	-2.07020100	-0.39517400	
H	4.60245200	-2.32562800	0.51376300	
O	2.69735500	-1.06387500	2.14724200	
H	2.44565700	-0.13439100	2.28790400	
**Name**				**MB-C5-OH-RAF**
Cartesian Coordinates				Frequency and Energy
C	3.50997900	1.46464300	-0.16622600	Zero-point correction= 0.327606 (Hartree/Particle)
C	3.69263100	0.03361700	-0.12687600	Thermal correction to Energy= 0.347996
C	2.53428500	-0.79935300	-0.10491200	Thermal correction to Enthalpy= 0.348941
C	2.26695200	1.99907700	-0.16886500	Thermal correction to Gibbs Free Energy= 0.277941
C	1.24516400	-0.23281300	-0.05488600	Sum of electronic and zero-point Energies= -1258.087412
S	-0.08427100	-1.28395400	-0.43322700	Sum of electronic and thermal Energies= -1258.067022
N	-0.08767200	1.82354700	-0.12858700	Sum of electronic and thermal Enthalpies= -1258.066078
C	1.07091800	1.20277000	-0.14289300	Sum of electronic and thermal Free Energies= -1258.137078
C	-1.45333500	-0.22779800	-0.18281200	
C	-2.70617400	-0.80169900	-0.16685500	
C	-3.86490400	0.00727600	-0.03840500	
C	-3.69019900	1.42672300	0.06297700	
C	-2.44448100	1.97466900	0.03401300	
C	-1.26907100	1.18212800	-0.08204700	
N	-5.09094200	-0.53854000	-0.00886400	
C	-5.25516500	-1.98525900	-0.11286000	
C	-6.27576100	0.30448600	0.13526600	
N	4.90597400	-0.50638600	-0.11605300	
C	6.09919700	0.34269600	-0.18473900	
C	5.08578000	-1.95960600	-0.03432700	
H	4.37270000	2.11445900	-0.18597400	
H	2.13459000	3.07446900	-0.19212500	
H	2.62978400	-1.87591700	-0.13707700	
H	-2.79912900	-1.87646800	-0.24690800	
H	-4.55199100	2.07162400	0.15866100	
H	-2.31520300	3.04822700	0.10740700	
H	-6.31486200	-2.21999100	-0.07879500	
H	-4.84473100	-2.35779500	-1.05453500	
H	-4.75663400	-2.49573900	0.71493200	
H	-6.24551200	0.86641000	1.07152100	
H	-6.36010400	1.00741200	-0.69665700	
H	-7.15504700	-0.33281600	0.14138400	
H	6.14820900	0.99775600	0.68685700	
H	6.97617500	-0.29564100	-0.20102300	
H	6.08128600	0.94831500	-1.09173700	
H	4.68546500	-2.44346700	-0.92697900	
H	6.14698100	-2.17254500	0.03943800	
H	4.58152100	-2.35237300	0.84937900	
O	1.44318200	-0.54750300	1.94104300	
H	2.02294200	0.17768600	2.22672900	
**Name**				**MB-C14-OH-RAF**

Cartesian Coordinates	Frequency and Energy
C -3.53674800 -1.33464300 -0.23827400	Zero-point correction= 0.325501 (Hartree/Particle)
C -3.70826000 0.09552400 -0.17498800	Thermal correction to Energy= 0.346736
C -2.54591400 0.91625100 -0.09845500	Thermal correction to Enthalpy= 0.347680
C -2.29843600 -1.87754000 -0.21330700	Thermal correction to Gibbs Free Energy= 0.274780
C -1.29768500 0.35338700 -0.06988000	Sum of electronic and zero-point Energies= -1258.096254
S 0.07123800 1.41724400 0.05679000	Sum of electronic and thermal Energies= -1258.075020
N 0.06432200 -1.70655800 -0.26737000	Sum of electronic and thermal Enthalpies= -1258.074075
C -1.10976900 -1.07703100 -0.10383400	Sum of electronic and thermal Free Energies= -1258.146976
C 1.42801600 0.33109100 -0.05564100	
C 2.68390100 0.87329100 0.01327900	
C 3.83961500 0.04695300 -0.11254200	
C 3.65330000 -1.36749300 -0.32815700	
C 2.40833900 -1.89081100 -0.38801600	
C 1.22809300 -1.08504700 -0.24315000	
N 5.06184300 0.56703100 -0.03886100	
C 5.24699000 1.99744400 0.21339000	
C 6.24933400 -0.27411000 -0.21661000	
N -4.92640400 0.63332700 -0.19257300	
C -6.11963100 -0.21296200 -0.26933500	
C -5.09860200 2.08580500 -0.14709500	
H -4.40100200 -1.97829800 -0.31523100	
H -2.16181800 -2.95094000 -0.27557300	
H -2.64574900 1.99247500 -0.05890800	
H 2.79607000 1.94079000 0.14734200	
H 4.51156900 -2.01593000 -0.42943000	
H 2.26103600 -2.95394400 -0.53636900	
H 6.30911300 2.20095300 0.30411500	
H 4.84036100 2.58846000 -0.60991300	
H 4.75119700 2.28640800 1.14150400	
H 6.30960800 -1.02648000 0.57201700	
H 6.22457700 -0.77073200 -1.18747900	
H 7.13040100 0.35780900 -0.16841900	
H -6.16250200 -0.89706300 0.57982000	
H -6.99619800 0.42670900 -0.24709500	
H -6.12723700 -0.78963000 -1.19607100	
H -4.61963700 2.55786600 -1.00731100	
H -6.16019700 2.31076900 -0.16919000	
H -4.66961800 2.49546500 0.76949500	
O -1.09597200 -1.04648500 2.20022800	
H -1.09560300 -2.01597300 2.26354900	

**Name**	**MB-N13-OH-RAF**
Cartesian Coordinates	Frequency and Energy
C 3.60981000 1.22659100 -0.22403500	Zero-point correction= 0.326833 (Hartree/Particle)
C 3.78167200 -0.19259100 -0.07611500	Thermal correction to Energy= 0.347522
C 2.62153100 -0.99682800 0.05507700	Thermal correction to Enthalpy= 0.348466
C 2.36826900 1.77681000 -0.23680600	Thermal correction to Gibbs Free Energy= 0.276882
C 1.36653900 -0.42650200 0.04506000	Sum of electronic and zero-point Energies= -1258.080865
S 0.00001700 -1.49256100 0.23017800	Sum of electronic and thermal Energies= -1258.060177
N -0.00088300 1.59252400 -0.14227300	Sum of electronic and thermal Enthalpies= -1258.059232
C 1.19775000 0.97976900 -0.10472000	Sum of electronic and thermal Free Energies= -1258.130817
C -1.36656200 -0.42736700 0.04327700	
C -2.62107600 -0.99731400 0.05251300	

C -3.78164200 -0.19255900 -0.07686900<br>C -3.61029000 1.22755900 -0.22179900<br>C -2.36945700 1.77803500 -0.23358400<br>C -1.19805800 0.98049400 -0.10361900<br>N -5.00517100 -0.73534500 -0.06940400<br>C -5.16851300 -2.17950900 0.07837400<br>C -6.19589000 0.10263000 -0.21120600<br>N 5.00601300 -0.73544100 -0.06791300<br>C 6.19582400 0.10237300 -0.21524000<br>C 5.16906900 -2.17888600 0.08422600<br>H 4.47324300 1.86776900 -0.32858900<br>H 2.24193000 2.84643400 -0.34852600<br>H 2.71240400 -2.06903900 0.16409800<br>H -2.71229300 -2.06954800 0.16091300<br>H -4.47411100 1.86835800 -0.32541000<br>H -2.24167000 2.84748200 -0.34309600<br>H -6.22830400 -2.41454000 0.06421900<br>H -4.67925100 -2.71101900 -0.74124000<br>H -4.74518100 -2.52268200 1.02518300<br>H -6.26543300 0.82280300 0.60676200<br>H -6.18231700 0.64177200 -1.16057900<br>H -7.07269400 -0.53726100 -0.18710500<br>H 6.26803700 0.82483700 0.60058800<br>H 7.07303400 -0.53709800 -0.19225900<br>H 6.17966000 0.63936700 -1.16587200<br>H 4.68170200 -2.71327100 -0.73479500<br>H 6.22891900 -2.41404500 0.07321900<br>H 4.74379400 -2.51972500 1.03106700<br>O -0.00923800 2.99765700 0.97890500<br>H 0.07859500 3.70070200 0.31726600	
**Name**	**MB-C16-OH-FHT**
Cartesian Coordinates	Frequency and Energy
C -3.29820800 -1.19586400 -0.48892000<br>C -3.43085200 0.22449600 -0.36315300<br>C -2.25867500 0.99951300 -0.22317100<br>C -2.06717700 -1.77802400 -0.47761700<br>C -1.01676300 0.39407900 -0.21459700<br>S 0.37469300 1.42293300 -0.03897500<br>N 0.28983100 -1.68939600 -0.33596100<br>C -0.87377200 -1.01790100 -0.34155300<br>C 1.70265000 0.30524700 -0.05171500<br>C 2.96987200 0.81175800 0.08601600<br>C 4.09945500 -0.05453700 0.08207000<br>C 3.87798000 -1.47270800 -0.07821900<br>C 2.62515000 -1.96057500 -0.21317600<br>C 1.46675400 -1.11129000 -0.20548100<br>N 5.33282000 0.42486000 0.22307200<br>C 5.55597600 1.86295700 0.37890700<br>C 6.49261600 -0.47112100 0.23493800<br>N -4.65099500 0.81293000 -0.36967700<br>C -5.84552000 0.01481400 -0.35947000<br>C -4.76961500 2.23167900 -0.03622900<br>H -4.17432000 -1.82097000 -0.58534300<br>H -1.96598700 -2.85298600 -0.56839900	Zero-point correction= 0.322046 (Hartree/Particle)<br>Thermal correction to Energy= 0.343050<br>Thermal correction to Enthalpy= 0.343994<br>Thermal correction to Gibbs Free Energy= 0.270878<br>Sum of electronic and zero-point Energies= -1258.094695<br>Sum of electronic and thermal Energies= -1258.073690<br>Sum of electronic and thermal Enthalpies= -1258.072746<br>Sum of electronic and thermal Free Energies= -1258.145862

H -2.32360800 2.07437200 -0.12164800<br>H 3.10623200 1.87844400 0.20222500<br>H 4.71853500 -2.15133100 -0.09032700<br>H 2.45381300 -3.02377500 -0.33220100<br>H 6.62345600 2.04441600 0.45278000<br>H 5.16443300 2.40678200 -0.48292800<br>H 5.06954000 2.23094500 1.28478400<br>H 6.40866200 -1.19908500 1.04352200<br>H 6.58383500 -0.99855200 -0.71619900<br>H 7.38543000 0.12529100 0.39282500<br>H -5.95297800 -0.52774400 0.63565900<br>H -6.71848500 0.65209500 -0.47008300<br>H -5.84249800 -0.74801900 -1.13648100<br>H -4.23864500 2.84240100 -0.76779600<br>H -5.82014800 2.50590700 -0.05536800<br>H -4.36474600 2.43346100 0.95917800<br>O -5.69223900 -1.33218500 2.01715000<br>H -4.75050900 -1.51500400 1.84838700	
**Name**	**MB-C17-OH-FHT**
Cartesian Coordinates	Frequency and Energy
C -3.29274200 -1.72829700 -0.01757000<br>C -3.53236700 -0.34721500 -0.30880000<br>C -2.42106400 0.51295600 -0.44880700<br>C -2.02209200 -2.19282500 0.13038300<br>C -1.13556500 0.02588300 -0.29257600<br>S 0.17168700 1.15702500 -0.47122000<br>N 0.32403000 -1.89494800 0.18872400<br>C -0.88794400 -1.34307500 0.00632100<br>C 1.58111700 0.19080200 -0.16961400<br>C 2.80550300 0.80704500 -0.21729500<br>C 3.99731900 0.06470300 0.01764500<br>C 3.88375100 -1.34687400 0.30261200<br>C 2.67247100 -1.94346100 0.34657500<br>C 1.45272300 -1.21955500 0.11697200<br>N 5.19065100 0.65147800 -0.02283100<br>C 5.30337100 2.07901700 -0.32535700<br>C 6.41484200 -0.11069700 0.24123900<br>N -4.79786700 0.12121900 -0.43034400<br>C -5.93131800 -0.73678900 -0.07995000<br>C -5.02902200 1.53126600 -0.56071500<br>H -4.12134900 -2.41232100 0.09452300<br>H -1.84060800 -3.23683200 0.35652200<br>H -2.55852400 1.56298400 -0.67045600<br>H 2.86015100 1.86606100 -0.43095000<br>H 4.77407200 -1.93246200 0.48080800<br>H 2.58293000 -3.00171100 0.56078800<br>H 6.35400800 2.35012100 -0.34172100<br>H 4.86728200 2.29857600 -1.30171200<br>H 4.79498000 2.67455800 0.43582100<br>H 6.38267800 -0.55705500 1.23623900<br>H 6.54793000 -0.89818300 -0.50263500<br>H 7.26083600 0.56746100 0.18962900<br>H -5.86300400 -1.07175000 0.95797600<br>H -6.84698000 -0.16780000 -0.21122500	Zero-point correction= 0.322140 (Hartree/Particle)<br>Thermal correction to Energy= 0.342976<br>Thermal correction to Enthalpy= 0.343920<br>Thermal correction to Gibbs Free Energy= 0.271418<br>Sum of electronic and zero-point Energies= -1258.094924<br>Sum of electronic and thermal Energies= -1258.074088<br>Sum of electronic and thermal Enthalpies= -1258.073144<br>Sum of electronic and thermal Free Energies= -1258.145646

H -5.97137700 -1.60710600 -0.73608500<br>H -4.47280800 1.96974100 -1.38932100<br>H -6.09049800 1.72775300 -0.68057100<br>H -4.68853400 2.07381100 0.38287800<br>O -3.91296400 2.57519700 1.71520600<br>H -3.15016000 1.97243300 1.65092700	
**Name**	**MB-C2-C1-OH-RAF**
Cartesian Coordinates	Frequency and Energy
C 3.61277700 1.26217700 -0.46872500<br>C 3.66742000 -0.23116400 -0.23215100<br>C 2.45501400 -0.97367900 -0.13095900<br>C 2.23719600 1.83397700 -0.38296000<br>C 1.23482000 -0.36232500 -0.18471600<br>S -0.14493600 -1.39577400 -0.05616800<br>N -0.11630000 1.75368200 -0.39622100<br>C 1.08165100 1.09849000 -0.33099500<br>C -1.49882600 -0.27900200 -0.11929500<br>C -2.75358900 -0.82555800 -0.00282800<br>C -3.91082600 -0.00248900 -0.04821500<br>C -3.72236300 1.40558500 -0.23152100<br>C -2.47259800 1.92806900 -0.34694400<br>C -1.28604700 1.12591400 -0.29067100<br>N -5.14302000 -0.52930700 0.07903000<br>C -5.31784300 -1.96553900 0.27960300<br>C -6.32507400 0.32971000 0.04065300<br>N 4.82825400 -0.82387100 -0.16107800<br>C 6.11847200 -0.13112200 -0.24924500<br>C 4.93217000 -2.28423800 -0.01181500<br>H 4.23198000 1.75441000 0.28811800<br>H 2.16244300 2.90424800 -0.53368800<br>H 2.49920700 -2.04706900 -0.00279300<br>H -2.85618600 -1.89578500 0.12576300<br>H -4.57697800 2.06626200 -0.27483800<br>H -2.33666800 2.99457500 -0.48126500<br>H -6.37930400 -2.18181800 0.34927300<br>H -4.89735700 -2.52482100 -0.55869400<br>H -4.82887300 -2.28704300 1.20210700<br>H -6.29017000 1.06918800 0.84353400<br>H -6.39422200 0.84802000 -0.91778200<br>H -7.20748300 -0.28890400 0.17049300<br>H 6.00541500 0.94328200 -0.17273400<br>H 6.74468600 -0.49601000 0.56427800<br>H 6.58598000 -0.38401000 -1.20220400<br>H 4.42086700 -2.78051900 -0.83590100<br>H 5.98457100 -2.55049600 -0.03141100<br>H 4.49615700 -2.59354000 0.93828500<br>O 2.07477200 2.02425300 1.89936900<br>H 2.06703800 1.06905700 2.07410600<br>O 4.16671200 1.47059300 -1.76780600<br>H 4.34641500 2.41445200 -1.86720900	Zero-point correction= 0.340838 (Hartree/Particle)<br>Thermal correction to Energy= 0.363776<br>Thermal correction to Enthalpy= 0.364721<br>Thermal correction to Gibbs Free Energy= 0.286447<br>Sum of electronic and zero-point Energies= -1333.865484<br>Sum of electronic and thermal Energies= -1333.842546<br>Sum of electronic and thermal Enthalpies= -1333.841602<br>Sum of electronic and thermal Free Energies= -1333.919876
**Name**	**MB-C2-C3-OH-RAF**
Cartesian Coordinates	Frequency and Energy
C 3.51741900 1.57871900 -0.18156100<br>C 3.66370300 0.05966200 -0.07328700	Zero-point correction= 0.343142 (Hartree/Particle)<br>Thermal correction to Energy= 0.364660

C 2.43239300 -0.73067800 0.01876500<br>C 2.11287400 2.06316800 -0.01479800<br>C 1.14224700 -0.15354600 -0.04526800<br>S -0.17800700 -1.26362400 0.03981300<br>N -0.22811900 1.90451400 -0.02156900<br>C 0.96719900 1.27020600 -0.03939600<br>C -1.56693300 -0.18677500 -0.00689900<br>C -2.80939900 -0.78414700 -0.00804900<br>C -3.98836000 0.00281700 -0.02372100<br>C -3.83490600 1.42153200 -0.03955100<br>C -2.59317900 1.99135200 -0.03767300<br>C -1.39433900 1.22770300 -0.02301700<br>N -5.21173000 -0.56795300 -0.02359500<br>C -5.34574600 -2.01932900 0.00423700<br>C -6.41091900 0.26286200 -0.03859400<br>N 4.81962400 -0.48287800 -0.54191100<br>C 6.05733000 0.18429500 -0.11125200<br>C 4.95264900 -1.94404500 -0.53714900<br>H 4.15018100 2.03302700 0.58871400<br>H 1.98591800 3.13848500 0.02713100<br>H 2.51851300 -1.80821500 -0.03102600<br>H -2.87826900 -1.86423600 0.00511100<br>H -4.70725500 2.05999900 -0.05190500<br>H -2.49120600 3.07031000 -0.04955300<br>H -6.40203400 -2.27267900 0.00598700<br>H -4.87946500 -2.47415800 -0.87371800<br>H -4.88497300 -2.43935300 0.90228600<br>H -6.45594000 0.90608200 0.84394300<br>H -6.44339600 0.89067100 -0.93250300<br>H -7.28236300 -0.38558600 -0.03948200<br>H 6.25771600 -0.01955600 0.94594000<br>H 6.87935800 -0.20691700 -0.70784400<br>H 6.00030800 1.25677800 -0.27410200<br>H 4.17823000 -2.40681600 -1.14556200<br>H 5.91489500 -2.18896900 -0.98199600<br>H 4.92273300 -2.35019200 0.47908500<br>O 3.35364000 -0.36686500 1.46890400<br>H 2.89969200 0.34586300 1.95354200<br>O 3.98775800 1.93899300 -1.47588800<br>H 3.95818500 2.90242700 -1.54183800	Thermal correction to Enthalpy= 0.365604<br>Thermal correction to Gibbs Free Energy= 0.291950<br>Sum of electronic and zero-point Energies= -1333.869049<br>Sum of electronic and thermal Energies= -1333.847531<br>Sum of electronic and thermal Enthalpies= -1333.846587<br>Sum of electronic and thermal Free Energies= -1333.920241
**Name**	**MB-C2-C4-OH-RAF**
Cartesian Coordinates	Frequency and Energy
C 3.52905500 1.61281200 -0.10760200<br>C 3.64424800 0.10826600 -0.21908800<br>C 2.46139000 -0.69026800 -0.18827900<br>C 2.12817700 2.10849700 0.01214300<br>C 1.20752400 -0.11648200 -0.16165000<br>S -0.12304200 -1.20462400 -0.24498800<br>N -0.21170000 1.93032200 0.06386900<br>C 1.00290400 1.32773900 -0.02902800<br>C -1.52302600 -0.15478000 -0.10746300<br>C -2.75675500 -0.76542600 -0.13991100<br>C -3.94420100 0.00178100 -0.02544800<br>C -3.80836500 1.41782300 0.11882100	Zero-point correction= 0.340266 (Hartree/Particle)<br>Thermal correction to Energy= 0.363169<br>Thermal correction to Enthalpy= 0.364113<br>Thermal correction to Gibbs Free Energy= 0.286783<br>Sum of electronic and zero-point Energies= -1333.871733<br>Sum of electronic and thermal Energies= -1333.848830<br>Sum of electronic and thermal Enthalpies= -1333.847885<br>Sum of electronic and thermal Free Energies= -1333.925216

C	-2.57658500	2.00168400	0.14460200	
C	-1.36549500	1.25512100	0.03283500	
N	-5.15842900	-0.58467900	-0.05186700	
C	-5.27347400	-2.02896300	-0.22002200	
C	-6.36928600	0.21912000	0.08411400	
N	4.82280500	-0.43918200	-0.33596000	
C	6.09028300	0.29699500	-0.25312800	
C	4.96772800	-1.89222900	-0.50700900	
H	4.07635700	1.91768200	0.79530000	
H	2.01545500	3.18189200	0.11345600	
H	2.54203600	-1.76255900	-0.29876500	
H	-2.81427100	-1.84076700	-0.24903400	
H	-4.68883300	2.03909000	0.20513100	
H	-2.48551400	3.07638600	0.25185900	
H	-6.32587800	-2.29662500	-0.23612500	
H	-4.81761500	-2.35011000	-1.15996100	
H	-4.78994200	-2.55975300	0.60420400	
H	-6.37125600	0.76636600	1.02959400	
H	-6.46063900	0.93472200	-0.73685600	
H	-7.23020800	-0.44297000	0.06561900	
H	5.95066800	1.28692700	0.16517700	
H	6.75735800	-0.27789600	0.38789000	
H	6.52657300	0.37499400	-1.24968900	
H	4.36620500	-2.23183100	-1.34890200	
H	6.01317500	-2.10732200	-0.70702300	
H	4.65675000	-2.40450900	0.40524900	
O	2.72531200	-0.89563800	2.18086800	
H	2.36706600	0.00042500	2.31251500	
O	4.15346800	2.16184300	-1.26588400	
H	4.35835300	3.08817100	-1.08588000	
**Name**				**MB-C2-C5-OH-RAF**
Cartesian Coordinates				Frequency and Energy
C	3.62286000	1.57013000	-0.24012000	Zero-point correction= 0.342154 (Hartree/Particle)
C	3.71657000	0.06121100	-0.08685000	Thermal correction to Energy= 0.364203
C	2.53111900	-0.72432800	-0.07897800	Thermal correction to Enthalpy= 0.365147
C	2.22942800	2.09661900	-0.18056100	Thermal correction to Gibbs Free Energy= 0.290758
C	1.26512100	-0.12471900	-0.10213500	Sum of electronic and zero-point Energies= -1333.864298
S	-0.05102100	-1.15706000	-0.49919200	Sum of electronic and thermal Energies= -1333.842249
N	-0.11433400	1.95481000	-0.04382300	Sum of electronic and thermal Enthalpies= -1333.841305
C	1.09759300	1.33950400	-0.12084800	Sum of electronic and thermal Free Energies= -1333.915694
C	-1.43990600	-0.12139300	-0.21373700	
C	-2.67725600	-0.72352600	-0.22387200	
C	-3.85562500	0.04667900	-0.04476200	
C	-3.70603600	1.45750400	0.14328700	
C	-2.47231700	2.03431000	0.14385100	
C	-1.26769500	1.28477900	-0.03326000	
N	-5.07315000	-0.53131300	-0.04860900	
C	-5.20385200	-1.97000500	-0.25321600	
C	-6.27417200	0.27420300	0.15415700	
N	4.89034000	-0.50900600	0.00897000	
C	6.16378100	0.21761900	-0.01529500	
C	5.02094900	-1.97033600	0.11726100	
H	4.19043100	2.02734700	0.57584200	
H	2.13344700	3.17587200	-0.20457600	

H 2.59264300 -1.80336500 -0.11495000<br>H -2.74521400 -1.79450400 -0.36549700<br>H -4.57950300 2.07910300 0.28259200<br>H -2.37092100 3.10434300 0.28329500<br>H -6.25801200 -2.23067300 -0.24407600<br>H -4.77914000 -2.26681900 -1.21527400<br>H -4.69861900 -2.52553300 0.54095300<br>H -6.24356800 0.78494100 1.11926700<br>H -6.38354200 1.02046100 -0.63645800<br>H -7.13984500 -0.38153200 0.13602400<br>H 6.77248300 -0.15775400 0.80649100<br>H 6.66676900 0.00316900 -0.96004100<br>H 6.02177000 1.28613400 0.08863000<br>H 4.59925400 -2.44828000 -0.76695000<br>H 6.07721600 -2.21111400 0.18685300<br>H 4.50563900 -2.32075800 1.01202900<br>O 1.56751300 -0.51713200 1.96537700<br>H 2.13132200 0.23193700 2.22168900<br>O 4.27525300 1.97859700 -1.43848100<br>H 3.72158900 1.71642400 -2.18638100	
**Name**	**MB-C2-C7-OH-RAF**
Cartesian Coordinates	Frequency and Energy
C 3.55075800 1.45110400 -0.25856000<br>C 3.70762800 0.03969100 -0.14087100<br>C 2.52741900 -0.76106700 -0.08541400<br>C 2.30274200 2.01092700 -0.29418800<br>C 1.24952000 -0.15761800 0.00372900<br>S -0.10244700 -1.22286000 -0.37753300<br>N -0.06263100 1.91532200 -0.21538100<br>C 1.09433900 1.26236100 -0.20027900<br>C -1.45959100 -0.16841800 -0.15460200<br>C -2.69043100 -0.76260500 -0.13524700<br>C -3.87868400 0.01099700 -0.02494100<br>C -3.78123100 1.51077900 0.15428500<br>C -2.39544600 2.04807000 0.05057500<br>C -1.27077600 1.29117000 -0.08848900<br>N -5.05850500 -0.55389900 -0.03715600<br>C -5.19732000 -2.01608000 -0.10908000<br>C -6.32890300 0.17557900 0.04388600<br>N 4.91434600 -0.54747600 -0.10993200<br>C 6.12073200 0.26474000 -0.25200300<br>C 5.05102000 -1.99364300 0.07068000<br>H 4.42066000 2.08856400 -0.32439200<br>H 2.20276900 3.08577800 -0.39554000<br>H 2.59445700 -1.84109700 -0.07614700<br>H -2.75919300 -1.83984500 -0.20365800<br>H -4.38528300 1.98361900 -0.63036100<br>H -2.30072100 3.12715300 0.09545600<br>H -6.25336400 -2.25934500 -0.04176300<br>H -4.80024400 -2.38469700 -1.05530000<br>H -4.66901100 -2.48306600 0.72163500<br>H -6.76162800 0.02917600 1.03473700<br>H -6.19543500 1.23472600 -0.14225700<br>H -6.99833500 -0.24572000 -0.70506900	Zero-point correction= 0.342244 (Hartree/Particle)<br>Thermal correction to Energy= 0.364308<br>Thermal correction to Enthalpy= 0.365252<br>Thermal correction to Gibbs Free Energy= 0.290777<br>Sum of electronic and zero-point Energies= -1333.857367<br>Sum of electronic and thermal Energies= -1333.835303<br>Sum of electronic and thermal Enthalpies= -1333.834359<br>Sum of electronic and thermal Free Energies= -1333.908834

H	6.20866800	0.96435800	0.58256800	
H	6.98541700	-0.39126600	-0.26129200	
H	6.08929000	0.82865500	-1.18608000	
H	4.66630000	-2.52982300	-0.79941100	
H	6.10388000	-2.22807600	0.19198800	
H	4.51314100	-2.31710100	0.96264900	
O	1.29521400	-0.36826700	1.93128800	
H	1.93783600	0.31252200	2.18679600	
O	-4.35495500	1.78960600	1.43459300	
H	-4.46256500	2.74647200	1.51014100	
**Name**				**MB-C2-C8-OH-RAF**
Cartesian Coordinates				Frequency and Energy
C	3.46638900	1.51996500	-0.13394500	Zero-point correction= 0.340180 (Hartree/Particle)
C	3.63785100	0.10504700	-0.23285900	Thermal correction to Energy= 0.363012
C	2.47327400	-0.70665800	-0.16411500	Thermal correction to Enthalpy= 0.363956
C	2.22190700	2.06681300	-0.02875700	Thermal correction to Gibbs Free Energy= 0.286592
C	1.20521800	-0.12036900	-0.11253100	Sum of electronic and zero-point Energies= -1333.871285
S	-0.15259000	-1.21436700	-0.13797000	Sum of electronic and thermal Energies= -1333.848452
N	-0.14436900	1.93151300	0.05367300	Sum of electronic and thermal Enthalpies= -1333.847508
C	1.02705700	1.28363900	-0.02312900	Sum of electronic and thermal Free Energies= -1333.924873
C	-1.52377500	-0.16011800	-0.04667700	
C	-2.75201000	-0.75925900	-0.06246800	
C	-3.94809100	0.00835700	-0.00724700	
C	-3.86874400	1.51556200	0.10221700	
C	-2.48023600	2.05501700	0.11144600	
C	-1.34352200	1.29785300	0.04073900	
N	-5.12344500	-0.56341800	-0.04463700	
C	-5.25686000	-2.02748000	-0.05809100	
C	-6.39617100	0.16499400	-0.09187500	
N	4.86025000	-0.44965500	-0.34643100	
C	6.05122800	0.39389100	-0.37647100	
C	5.00838300	-1.90022000	-0.39509700	
H	4.32859300	2.17192100	-0.14471600	
H	2.10493800	3.14158300	0.04770700	
H	2.54878100	-1.77758300	-0.29409500	
H	-2.81416500	-1.83657600	-0.13658600	
H	-4.38381200	1.93602900	-0.77273000	
H	-2.39032500	3.13240100	0.18962300	
H	-6.30720900	-2.27130500	0.07158300	
H	-4.90532600	-2.42699200	-1.01045500	
H	-4.68655900	-2.46311200	0.76067100	
H	-6.87987100	0.11053900	0.88427500	
H	-6.25239800	1.20235600	-0.37159100	
H	-7.02493000	-0.32801800	-0.83209000	
H	6.15768300	0.95587600	0.55483200	
H	6.92315500	-0.24107000	-0.50260300	
H	6.00953200	1.09794400	-1.21049400	
H	4.51800900	-2.31452700	-1.27978600	
H	6.06637100	-2.14204000	-0.43849300	
H	4.57850100	-2.36409600	0.49614000	
O	2.48752700	-1.02901300	2.12502500	
H	2.47114600	-0.06866400	2.27684900	
O	-4.55652600	1.88201400	1.29524900	
H	-4.76141900	2.82453500	1.24428200	

**Name**	**MB-C2-C9-OH-RAF**
Cartesian Coordinates	Frequency and Energy
C 3.40839800 1.46941800 -0.12343200	Zero-point correction= 0.340689 (Hartree/Particle)
C 3.60429100 0.02725800 -0.12197300	Thermal correction to Energy= 0.363002
C 2.40374800 -0.77147500 -0.17262300	Thermal correction to Enthalpy= 0.363946
C 2.16217500 2.00823000 -0.06207200	Thermal correction to Gibbs Free Energy= 0.288475
C 1.14621600 -0.17937300 -0.08967800	Sum of electronic and zero-point Energies= -1333.846584
S -0.22828300 -1.26841800 -0.05631500	Sum of electronic and thermal Energies= -1333.824272
N -0.22467600 1.87665100 0.01461400	Sum of electronic and thermal Enthalpies= -1333.823328
C 0.99072200 1.20564500 -0.04046400	Sum of electronic and thermal Free Energies= -1333.898799
C -1.58248600 -0.19584300 -0.04169700	
C -2.82891300 -0.75634800 -0.06558500	
C -4.01956800 0.02497400 -0.04222000	
C -3.94097900 1.53411600 0.04882400	
C -2.55192700 2.06218200 0.03587600	
C -1.37441200 1.26168100 0.00625500	
N -5.19611000 -0.54169900 -0.08374300	
C -5.34188800 -2.00556600 -0.07411700	
C -6.46666100 0.19073900 -0.13900200	
N 4.78798900 -0.49202600 -0.60791600	
C 6.00983900 0.24456300 -0.27524700	
C 4.95962100 -1.94146000 -0.49948400	
H 4.26831800 2.12270900 -0.13516900	
H 2.03085900 3.08338000 -0.03825600	
H 2.48290500 -1.84917500 -0.20966600	
H -2.91325100 -1.83322200 -0.12435900	
H -4.47055700 1.95440300 -0.81564600	
H -2.43537200 3.13733800 0.08532200	
H -6.39733200 -2.23769500 0.03228300	
H -4.97009600 -2.42449700 -1.00999400	
H -4.79638400 -2.43038300 0.76700800	
H -6.32095700 1.23342600 -0.39438200	
H -7.08405700 -0.28738000 -0.89841500	
H -6.96366600 0.11308100 0.82910400	
H 6.16915100 0.29131400 0.80699000	
H 6.85185100 -0.27005800 -0.73318500	
H 5.98679700 1.25363800 -0.68035500	
H 4.20968100 -2.46752000 -1.08818300	
H 5.93492000 -2.20112000 -0.90553100	
H 4.90810800 -2.28042800 0.54092100	
O 3.76404100 -0.25464400 1.77128700	
H 3.06538800 0.30527600 2.13838100	
O -4.60053600 1.90913900 1.25602100	
H -4.84328700 2.84171700 1.18798200	

**Name**	**MB-C2-C10-OH-RAF**
Cartesian Coordinates	Frequency and Energy
C -3.45783200 -1.26008200 -0.35346700	Zero-point correction= 0.340297 (Hartree/Particle)
C -3.57824400 0.16360400 -0.24965700	Thermal correction to Energy= 0.363107
C -2.38573000 0.91524600 -0.09912300	Thermal correction to Enthalpy= 0.364051
C -2.20574600 -1.85389900 -0.40608700	Thermal correction to Gibbs Free Energy= 0.286837
C -1.15618200 0.29478700 -0.10226300	Sum of electronic and zero-point Energies= -1333.869740
S 0.24617700 1.32654100 0.11899200	Sum of electronic and thermal Energies= -1333.846930
N 0.14695100 -1.80158300 -0.31274600	Sum of electronic and thermal Enthalpies= -1333.845986
C -1.00564800 -1.11533000 -0.26831700	Sum of electronic and thermal Free Energies= -1333.923200

C	1.58120000	0.23418400	-0.00361500	
C	2.82699400	0.78426400	0.10728500	
C	4.00139300	-0.01259800	0.01659300	
C	3.88134300	-1.50614300	-0.19280100	
C	2.47745700	-1.99566100	-0.29163400	
C	1.36362800	-1.20927200	-0.20751900	
N	5.19113400	0.52102100	0.11549500	
C	5.36223800	1.94757800	0.42654500	
C	6.44584100	-0.21186300	-0.08967500	
N	-4.78864400	0.75461300	-0.25848900	
C	-6.00203500	-0.04786800	-0.38680400	
C	-4.89907200	2.20374900	-0.13418600	
H	-4.32864100	-1.86550600	-0.55543900	
H	-2.12608300	-2.92627100	-0.53707400	
H	-2.43605700	1.99050300	0.01734000	
H	2.91972900	1.85329400	0.24340500	
H	4.38860200	-1.75004900	-1.13666100	
H	2.35703800	-3.06365600	-0.43318600	
H	6.40975100	2.11757500	0.65804900	
H	5.07520500	2.55574100	-0.43278300	
H	4.75908500	2.21685000	1.29188000	
H	6.91829200	-0.39998800	0.87524300	
H	6.27831300	-1.15041700	-0.60592600	
H	7.09694600	0.42150300	-0.69101500	
H	-6.07228900	-0.77826900	0.42233600	
H	-6.86184500	0.61364400	-0.33518600	
H	-6.02375600	-0.57562400	-1.34332100	
H	-4.37037000	2.70611600	-0.94795600	
H	-5.94864500	2.47887700	-0.17916600	
H	-4.48678600	2.54630300	0.81829400	
O	-3.64715300	-1.82112900	1.79774900	
H	-2.89826400	-1.26453700	2.06891200	
O	4.55302000	-2.13177800	0.89723000	
H	4.72545600	-3.05119700	0.65650100	
**Name**				**MB-C2-C11-OH-RAF**
Cartesian Coordinates				Frequency and Energy
C	3.52241200	1.15651200	-0.37921100	Zero-point correction= 0.342095 (Hartree/Particle)
C	3.62278900	-0.26026600	-0.18312700	Thermal correction to Energy= 0.364345
C	2.42323500	-0.98842900	0.00330700	Thermal correction to Enthalpy= 0.365289
C	2.28786400	1.80376200	-0.28192100	Thermal correction to Gibbs Free Energy= 0.290323
C	1.20139600	-0.34419800	-0.02324100	Sum of electronic and zero-point Energies= -1333.861922
S	-0.21164600	-1.35964500	0.18685500	Sum of electronic and thermal Energies= -1333.839672
N	-0.07496500	1.75241200	-0.28965100	Sum of electronic and thermal Enthalpies= -1333.838728
C	1.05739200	1.06091100	-0.21163300	Sum of electronic and thermal Free Energies= -1333.913694
C	-1.53924300	-0.26282000	0.01384200	
C	-2.78779500	-0.81196700	0.10523200	
C	-3.95562900	-0.01496900	-0.03753000	
C	-3.82296700	1.47317300	-0.27498100	
C	-2.41521700	1.95558600	-0.35349500	
C	-1.31025400	1.17378400	-0.21540100	
N	-5.15026400	-0.54303800	0.04003300	
C	-5.33435200	-1.96146800	0.37797000	
C	-6.39565900	0.18821800	-0.21843900	
N	4.82664000	-0.85957000	-0.19209400	

C	6.05498700	-0.08716200	-0.38583800	
C	4.91840600	-2.30469500	-0.00920800	
H	4.40560900	1.75046100	-0.56353500	
H	2.21688700	2.84331300	-0.57144000	
H	2.45521000	-2.06021200	0.14959500	
H	-2.88559600	-1.87755300	0.26257900	
H	-4.31556300	1.70117700	-1.23012800	
H	-2.29015300	3.01934300	-0.52215300	
H	-6.38878900	-2.12420500	0.58213300	
H	-5.02358900	-2.59020400	-0.45802900	
H	-4.75781000	-2.21257200	1.26673700	
H	-6.89837400	0.39590200	0.72697300	
H	-6.20824300	1.11683700	-0.74585500	
H	-7.02967900	-0.45418400	-0.82856000	
H	6.16315200	0.66663400	0.39609700	
H	6.89998200	-0.76699500	-0.33729700	
H	6.05406000	0.40339700	-1.36087300	
H	4.38460900	-2.82632000	-0.80721400	
H	5.96340300	-2.59679500	-0.03266900	
H	4.48883300	-2.59548100	0.95223000	
O	2.76479100	2.28104300	1.60103100	
H	2.69905000	1.40100600	2.00378800	
O	-4.52078500	2.11708400	0.79139600	
H	-4.63814600	3.04567600	0.55299900	
**Name**				**MB-C2-C12-OH-RAF**
Cartesian Coordinates				Frequency and Energy
C	-3.59460500	-1.31096100	-0.20473700	Zero-point correction= 0.340794 (Hartree/Particle)
C	-3.73959900	0.11315500	-0.17275100	Thermal correction to Energy= 0.363424
C	-2.55638600	0.89959900	-0.12954400	Thermal correction to Enthalpy= 0.364369
C	-2.36382900	-1.88536700	-0.14782000	Thermal correction to Gibbs Free Energy= 0.288419
C	-1.32230000	0.30239400	-0.07550100	Sum of electronic and zero-point Energies= -1333.862925
S	0.07458100	1.35564400	0.02242800	Sum of electronic and thermal Energies= -1333.840295
N	0.02352600	-1.79760400	-0.20215400	Sum of electronic and thermal Enthalpies= -1333.839351
C	-1.16409000	-1.11785100	0.01038600	Sum of electronic and thermal Free Energies= -1333.915300
C	1.41209200	0.26615200	-0.04212200	
C	2.65946800	0.81567000	0.03505800	
C	3.84055800	0.02892200	-0.09084300	
C	3.73652300	-1.46018500	-0.34710800	
C	2.33682000	-1.96798300	-0.36814600	
C	1.20047800	-1.18543800	-0.20834300	
N	5.02468000	0.57331900	-0.00166400	
C	5.19369500	1.98849400	0.35910700	
C	6.28092500	-0.13511600	-0.27430800	
N	-4.95671200	0.68880100	-0.20061000	
C	-6.16161100	-0.13343500	-0.27315100	
C	-5.08775100	2.14245900	-0.19828700	
H	-4.46849700	-1.94109400	-0.29277500	
H	-2.25604900	-2.96299200	-0.18942700	
H	-2.62353600	1.97988100	-0.15006200	
H	2.75304100	1.88752000	0.14889900	
H	4.18823500	-1.65846700	-1.32869800	
H	2.21835700	-3.03791400	-0.48768200	
H	6.23794500	2.14423900	0.61507500	
H	4.92668000	2.62500400	-0.48610900	

H	4.57597200	2.23182900	1.22123800	
H	6.77793700	-0.36878100	0.66798700	
H	6.11067300	-1.04524500	-0.83836800	
H	6.91068200	0.53747800	-0.85551700	
H	-6.21875000	-0.81419700	0.57884200	
H	-7.02862500	0.52022200	-0.25459400	
H	-6.18263700	-0.71848600	-1.19565100	
H	-4.62809300	2.57906300	-1.08859600	
H	-6.14337200	2.39709800	-0.18972200	
H	-4.61708500	2.57243600	0.68867500	
O	-0.91462000	-1.20526400	2.04444800	
H	-0.81423900	-2.16601500	2.12692100	
O	4.46849400	-2.12918800	0.67347300	
H	4.88266600	-2.91163700	0.28953900	
**Name**				**MB-C2-C14-OH-RAF**
Cartesian Coordinates				Frequency and Energy
C	-3.63833500	-1.42138200	-0.24619300	Zero-point correction= 0.340940 (Hartree/Particle)
C	-3.72112100	0.08999200	-0.21429300	Thermal correction to Energy= 0.363683
C	-2.53048900	0.86918700	-0.23079200	Thermal correction to Enthalpy= 0.364628
C	-2.24756300	-1.94850400	-0.29774300	Thermal correction to Gibbs Free Energy= 0.287770
C	-1.29408500	0.29198100	-0.20994100	Sum of electronic and zero-point Energies= -1333.868071
S	0.06201900	1.35765200	-0.14159800	Sum of electronic and thermal Energies= -1333.845327
N	0.10246700	-1.79242200	-0.35155500	Sum of electronic and thermal Enthalpies= -1333.844383
C	-1.09871100	-1.17289900	-0.21072000	Sum of electronic and thermal Free Energies= -1333.921241
C	1.44005200	0.27123700	-0.13975300	
C	2.68292600	0.84971300	-0.04370500	
C	3.85853700	0.05320300	-0.09417100	
C	3.70199900	-1.36048600	-0.26198700	
C	2.46291100	-1.91444200	-0.35162100	
C	1.26173600	-1.13980900	-0.28334200	
N	5.07831000	0.61084000	0.00632200	
C	5.21760900	2.04882500	0.21852200	
C	6.28104800	-0.21213000	-0.10340700	
N	-4.89857000	0.65658400	-0.17001700	
C	-6.16379500	-0.07532800	-0.03863500	
C	-5.04465000	2.11868900	-0.22971900	
H	-4.11562600	-1.79818700	0.66890200	
H	-2.14386100	-3.02419100	-0.37686500	
H	-2.60582500	1.94801800	-0.21075800	
H	2.76220000	1.92461400	0.05704400	
H	4.57229500	-2.00011100	-0.30669200	
H	2.35046800	-2.98602700	-0.46786600	
H	6.27122400	2.28495200	0.33322300	
H	4.82095000	2.60890300	-0.63184300	
H	4.68903300	2.35697800	1.12317600	
H	6.33527600	-0.93488900	0.71411200	
H	6.29589000	-0.74960800	-1.05351300	
H	7.15087700	0.43623100	-0.05625000	
H	-6.00367500	-1.09590800	0.28879100	
H	-6.76791400	0.45194800	0.69847200	
H	-6.68322500	-0.07227500	-0.99780200	
H	-4.49872100	2.51564900	-1.08395500	
H	-6.10008200	2.34741100	-0.34533900	
H	-4.67355700	2.56980000	0.69168600	

O -1.33879000 -1.20468100 1.99219200<br>H -1.00341500 -2.11128400 2.09080700<br>O -4.37044300 -1.85136900 -1.39094400<br>H -4.58109500 -2.78683500 -1.27662800	
**Name**	**MB-C2-N13-OH-RAF**
Cartesian Coordinates	Frequency and Energy
C 3.71419000 1.32965000 -0.13089600<br>C 3.79222200 -0.18008300 -0.08158200<br>C 2.62392400 -0.93510800 0.08606500<br>C 2.32949000 1.84571900 -0.30241000<br>C 1.37016800 -0.33268500 0.13159100<br>S 0.02423200 -1.39587500 0.40491800<br>N -0.03417300 1.65381700 -0.20606900<br>C 1.21290900 1.06990700 -0.09796900<br>C -1.35928700 -0.38161000 0.09601600<br>C -2.60182900 -0.96430600 0.14469100<br>C -3.77717800 -0.19326900 -0.07004300<br>C -3.63024800 1.21707800 -0.31724200<br>C -2.40246000 1.78714500 -0.35099700<br>C -1.21028600 1.02044200 -0.15384300<br>N -4.98702300 -0.75976600 -0.04326900<br>C -5.12923300 -2.19395000 0.21050200<br>C -6.19425300 0.03833200 -0.27266900<br>N 4.98749100 -0.75662000 -0.16434200<br>C 6.25736100 -0.03617800 -0.25289200<br>C 5.11342600 -2.20794900 -0.03599100<br>H 2.21954800 2.89708200 -0.53759800<br>H 2.68373000 -2.01143900 0.17465500<br>H -2.67995600 -2.02216200 0.35801000<br>H -4.50358000 1.83124500 -0.48359600<br>H -2.29162000 2.84638100 -0.54065600<br>H -6.18424100 -2.44744400 0.20035900<br>H -4.61725000 -2.77051700 -0.56235100<br>H -4.71210000 -2.45176900 1.18580000<br>H -6.29668500 0.80788400 0.49445200<br>H -6.15829400 0.51180500 -1.25500900<br>H -7.05661800 -0.61925000 -0.23041700<br>H 6.79700800 -0.13661600 0.69189700<br>H 6.85353100 -0.48409900 -1.04818000<br>H 6.11224400 1.01616600 -0.46593800<br>H 4.55759200 -2.71211600 -0.82858500<br>H 6.16452600 -2.47090700 -0.11878500<br>H 4.73893800 -2.54104800 0.93441900<br>O -0.12987300 3.08578900 0.76800800<br>H 0.13721900 3.74213900 0.10797400<br>H 4.33174500 1.69834800 -0.95506900<br>O 4.29515500 1.78365900 1.11219800<br>H 4.36540000 2.74604400 1.06541500	Zero-point correction= 0.342165 (Hartree/Particle)<br>Thermal correction to Energy= 0.364293<br>Thermal correction to Enthalpy= 0.365237<br>Thermal correction to Gibbs Free Energy= 0.290746<br>Sum of electronic and zero-point Energies= -1333.840546<br>Sum of electronic and thermal Energies= -1333.818419<br>Sum of electronic and thermal Enthalpies= -1333.817475<br>Sum of electronic and thermal Free Energies= -1333.891965
**Name**	**MB-C2-C16-OH-FHT**
Cartesian Coordinates	Frequency and Energy
C -3.37617000 -1.27520700 -0.62538400<br>C -3.42575500 0.21457900 -0.36444500<br>C -2.22961900 0.94729300 -0.24572700<br>C -2.00879000 -1.85868900 -0.54130700	Zero-point correction= 0.336554 (Hartree/Particle)<br>Thermal correction to Energy= 0.359232<br>Thermal correction to Enthalpy= 0.360176<br>Thermal correction to Gibbs Free Energy= 0.282744

C	-1.00380000	0.31927400	-0.26296600	Sum of electronic and zero-point Energies= -1333.861134
S	0.37287600	1.34037000	-0.12075700	Sum of electronic and thermal Energies= -1333.838456
N	0.33017200	-1.79721800	-0.36240800	Sum of electronic and thermal Enthalpies= -1333.837512
C	-0.85720500	-1.13586100	-0.39000000	Sum of electronic and thermal Free Energies= -1333.914944
C	1.72346900	0.22167800	-0.09558200	
C	2.97811700	0.77094600	0.04744700	
C	4.12859200	-0.05815500	0.07337600	
C	3.93376100	-1.46982900	-0.05214500	
C	2.68273100	-1.99198600	-0.19159000	
C	1.50712000	-1.18225900	-0.22124300	
N	5.36298000	0.46621800	0.21289700	
C	5.53831600	1.90742700	0.35346700	
C	6.53447900	-0.40375900	0.25027500	
N	-4.60481200	0.80042500	-0.26108000	
C	-5.84269700	0.05854400	-0.22210000	
C	-4.69615900	2.23461800	0.05002600	
H	-4.01214000	-1.76726000	0.12262900	
H	-1.94197300	-2.93427600	-0.65819900	
H	-2.26603000	2.02068700	-0.11601600	
H	3.07985600	1.84463900	0.14117100	
H	4.78394500	-2.13727300	-0.03554700	
H	2.54626700	-3.06316000	-0.28493600	
H	6.59906700	2.12454000	0.43930600	
H	5.14362700	2.43532900	-0.51813400	
H	5.03089200	2.27816000	1.24807800	
H	6.47770000	-1.10293800	1.08822000	
H	6.62954900	-0.97314200	-0.67728200	
H	7.42047600	0.21240000	0.37138700	
H	-6.05759400	-0.22163100	0.87481400	
H	-6.66739700	0.69208000	-0.53886900	
H	-5.80558500	-0.85538300	-0.80197000	
H	-4.21436000	2.81605700	-0.73560100	
H	-5.74545100	2.50660800	0.10457700	
H	-4.21600300	2.43895200	1.00798300	
O	-6.02649300	-0.87518800	2.22955900	
H	-5.52523100	-1.66759100	1.96436100	
O	-3.92214400	-1.47667400	-1.93198200	
H	-4.10472500	-2.41964000	-2.03595300	
**Name**				**MB-C2-C17-OH-FHT**
Cartesian Coordinates				Frequency and Energy
C	-3.38610900	-1.81689900	0.06030000	Zero-point correction= 0.336303 (Hartree/Particle)
C	-3.54520900	-0.34548700	-0.25001600	Thermal correction to Energy= 0.358926
C	-2.40318900	0.47157300	-0.36495100	Thermal correction to Enthalpy= 0.359871
C	-1.97104100	-2.27924300	0.12286500	Thermal correction to Gibbs Free Energy= 0.283330
C	-1.13305300	-0.04779600	-0.24080400	Sum of electronic and zero-point Energies= -1333.861825
S	0.15853700	1.07510500	-0.40170100	Sum of electronic and thermal Energies= -1333.839202
N	0.36303000	-2.01844100	0.14146800	Sum of electronic and thermal Enthalpies= -1333.838258
C	-0.87480100	-1.46965700	0.00969900	Sum of electronic and thermal Free Energies= -1333.914799
C	1.59476700	0.09999400	-0.15205600	
C	2.80257100	0.76026400	-0.19245900	
C	4.01670400	0.05132700	-0.00646300	
C	3.93435600	-1.35855500	0.22028300	
C	2.72793000	-1.99172900	0.25495100	
C	1.48963200	-1.30396100	0.07516600	

N 5.20707400 0.68400700 -0.04171000<br>C 5.26809300 2.12221900 -0.27726900<br>C 6.44368500 -0.05699700 0.18952300<br>N -4.76373500 0.15101900 -0.37250600<br>C -5.99095900 -0.64627500 -0.22994900<br>C -4.97168500 1.57073900 -0.52547900<br>H -1.82661600 -3.33567300 0.31834100<br>H -2.51012300 1.53357900 -0.54239800<br>H 2.81770300 1.82901600 -0.36506900<br>H 4.83668100 -1.93572500 0.36595700<br>H 2.67842500 -3.06034800 0.42908600<br>H 6.30944700 2.42994800 -0.29776700<br>H 4.81034000 2.37944600 -1.23553200<br>H 4.75539500 2.67250200 0.51622300<br>H 6.43945900 -0.52710200 1.17584200<br>H 6.58528900 -0.83069200 -0.56882600<br>H 7.27872200 0.63584400 0.13870600<br>H -6.40033100 -0.50667700 0.77140700<br>H -6.70472600 -0.28780100 -0.96860700<br>H -5.79669500 -1.69912300 -0.40106700<br>H -4.38473800 1.99789800 -1.33654000<br>H -6.02983800 1.78260800 -0.64545700<br>H -4.64374800 2.10996200 0.45106300<br>O -4.08901300 2.63560900 1.69861500<br>H -3.26627900 2.11444900 1.72656200<br>H -3.89887000 -2.38382100 -0.72774000<br>O -4.03135200 -2.03497800 1.31551500<br>H -4.18665200 -2.98320500 1.41316600	
**Name**	**MB-C2-C19-OH-FHT**
Cartesian Coordinates	Frequency and Energy
C -3.32386800 -1.72268800 -0.07178900<br>C -3.53152900 -0.34425600 -0.35491700<br>C -2.39997900 0.49260900 -0.43286200<br>C -2.06065000 -2.21567300 0.10765600<br>C -1.12884500 -0.02482100 -0.24887300<br>S 0.19983500 1.11212500 -0.36211700<br>N 0.29364000 -1.98407400 0.22642200<br>C -0.90315800 -1.39592300 0.02845700<br>C 1.59787600 0.13120000 -0.09563600<br>C 2.80781900 0.76706900 -0.14422400<br>C 4.02544200 0.05978600 0.04555700<br>C 3.99338500 -1.42196600 0.35147600<br>C 2.62543800 -2.00963200 0.38032800<br>C 1.46321300 -1.31085600 0.17082100<br>N 5.18223500 0.66516200 -0.03400900<br>C 5.26603700 2.11753400 -0.24646200<br>C 6.47959600 -0.01306700 0.06373500<br>N -4.79735600 0.15189200 -0.54022700<br>C -5.94120300 -0.65812500 -0.12445000<br>C -4.98948000 1.57212600 -0.60502800<br>H -4.16429900 -2.39763600 0.00498000<br>H -1.91187200 -3.26735500 0.32293800<br>H -2.50722700 1.55108300 -0.63209700<br>H 2.83575100 1.82822700 -0.35291600	Zero-point correction= 0.336292 (Hartree/Particle)<br>Thermal correction to Energy= 0.358874<br>Thermal correction to Enthalpy= 0.359818<br>Thermal correction to Gibbs Free Energy= 0.283535<br>Sum of electronic and zero-point Energies= -1333.866166<br>Sum of electronic and thermal Energies= -1333.843585<br>Sum of electronic and thermal Enthalpies= -1333.842640<br>Sum of electronic and thermal Free Energies= -1333.918924

H 4.56074200 -1.93444700 -0.43767800<br>H 2.56844100 -3.07021600 0.59640300<br>H 6.30763800 2.41130600 -0.15538800<br>H 4.90193300 2.37200500 -1.24285300<br>H 4.68093800 2.64130100 0.50817100<br>H 6.93038100 0.20993000 1.03179400<br>H 6.37949400 -1.08573700 -0.05432100<br>H 7.11536900 0.38209600 -0.72781700<br>H -5.88179700 -0.91215900 0.93842300<br>H -6.84954700 -0.08942900 -0.30462200<br>H -5.99682100 -1.57747800 -0.70732500<br>H -4.37219800 2.04061900 -1.37111100<br>H -6.03677300 1.80032300 -0.78560200<br>H -4.71118900 2.06434500 0.38713400<br>O -4.08205700 2.46105500 1.81021100<br>H -3.33536700 1.83671400 1.76552700<br>O 4.63718600 -1.59794500 1.61068600<br>H 4.86579400 -2.53182300 1.70368900	
**Name**	**MB-C2-C20-OH-FHT**
Cartesian Coordinates	Frequency and Energy
C -3.33900800 -1.19288800 -0.54027300<br>C -3.44211200 0.22012600 -0.41602700<br>C -2.25222000 0.95862200 -0.25511900<br>C -2.11597900 -1.80778200 -0.51678300<br>C -1.02565800 0.31995100 -0.23592100<br>S 0.38507900 1.34068300 -0.03079300<br>N 0.24546900 -1.79360400 -0.35472100<br>C -0.90251700 -1.08687800 -0.36616600<br>C 1.70165200 0.22093900 -0.03854000<br>C 2.95300800 0.74988800 0.11968300<br>C 4.11326400 -0.07043500 0.10641600<br>C 3.97298200 -1.56625400 -0.07311700<br>C 2.56647600 -2.03340500 -0.22288300<br>C 1.46094400 -1.22144400 -0.21168700<br>N 5.30869700 0.44188600 0.24865900<br>C 5.49415800 1.87400700 0.52385900<br>C 6.55639700 -0.32196700 0.13041000<br>N -4.65983200 0.85088100 -0.44730600<br>C -5.85940300 0.06680600 -0.35661700<br>C -4.74181000 2.24846100 -0.03173200<br>H -4.22505800 -1.80168000 -0.65111600<br>H -2.04709400 -2.88523100 -0.61067200<br>H -2.28706500 2.03458200 -0.14436900<br>H 3.05949200 1.82005100 0.23628600<br>H 4.51408000 -1.83990200 -0.99008500<br>H 2.43102700 -3.10253300 -0.33865200<br>H 6.53438300 2.03323700 0.79283500<br>H 5.25351600 2.46065600 -0.36424200<br>H 4.86167800 2.18095700 1.35509800<br>H 6.97544100 -0.48749700 1.12373600<br>H 6.39680500 -1.27390300 -0.36314200<br>H 7.25014700 0.27847600 -0.45665600<br>H -5.92869800 -0.45310600 0.65722900<br>H -6.73193100 0.70995200 -0.43907400	Zero-point correction= 0.336183 (Hartree/Particle)<br>Thermal correction to Energy= 0.358892<br>Thermal correction to Enthalpy= 0.359837<br>Thermal correction to Gibbs Free Energy= 0.282141<br>Sum of electronic and zero-point Energies= -1333.866055<br>Sum of electronic and thermal Energies= -1333.843345<br>Sum of electronic and thermal Enthalpies= -1333.842401<br>Sum of electronic and thermal Free Energies= -1333.920097

H -5.90415900 -0.71540900 -1.11308800<br>H -4.14958100 2.88153000 -0.69324200<br>H -5.77829500 2.56832700 -0.09490400<br>H -4.38850000 2.37840600 0.99636000<br>O -5.64437700 -1.20942500 2.04350000<br>H -4.71584600 -1.42458800 1.84202100<br>O 4.58339400 -2.18177800 1.05714100<br>H 4.76820600 -3.10357100 0.83542900	
**Name**	**MB-C4-C1-OH-RAF**
Cartesian Coordinates	Frequency and Energy
C 3.49730300 1.14476800 -0.45863200<br>C 3.66353200 -0.26347200 -0.23047200<br>C 2.44305000 -1.15081300 -0.01839300<br>C 2.25135400 1.70719000 -0.42069000<br>C 1.12790100 -0.42227600 -0.14065300<br>S -0.24556100 -1.47798200 -0.04991200<br>N -0.11517700 1.67299300 -0.35330300<br>C 1.01131300 0.93761300 -0.29763300<br>C -1.56721100 -0.31426200 -0.03947600<br>C -2.84370100 -0.83410200 0.09885900<br>C -3.97391200 0.01056000 0.08584200<br>C -3.74809800 1.40376200 -0.09403600<br>C -2.47735200 1.89933600 -0.23091100<br>C -1.32820700 1.07347500 -0.20897500<br>N -5.23522000 -0.48730700 0.24586500<br>C -5.43864300 -1.92805200 0.15114500<br>C -6.37657200 0.36535900 -0.07064500<br>N 4.84996100 -0.79596100 -0.18888900<br>C 6.05951900 -0.00821800 -0.48966000<br>C 5.10591800 -2.19462200 0.18115100<br>H 4.35779100 1.77503200 -0.62678600<br>H 2.13799500 2.75831400 -0.65322400<br>H -2.96106700 -1.90373900 0.21338800<br>H -4.58116000 2.09169800 -0.12617500<br>H -2.32355300 2.96388400 -0.36612000<br>H -6.49544600 -2.14141400 0.29096400<br>H -5.12420300 -2.31871500 -0.82357800<br>H -4.88507100 -2.45106100 0.93229300<br>H -6.39250300 1.24874200 0.56818100<br>H -6.36576900 0.68750400 -1.11790400<br>H -7.28893500 -0.19572400 0.11511200<br>H 6.28050900 0.65563500 0.34717400<br>H 6.88088000 -0.70374700 -0.63325100<br>H 5.91525200 0.56692000 -1.40077300<br>H 5.19316400 -2.80223300 -0.72003700<br>H 6.04736300 -2.21578800 0.72559200<br>H 4.32073600 -2.57840700 0.82593200<br>O 2.74223800 2.20703700 1.62609000<br>H 2.58093700 1.32659900 2.01012700<br>H 2.50976200 -1.53599100 1.00913500<br>O 2.46739000 -2.22067900 -0.94764900<br>H 2.63075000 -3.05437500 -0.49027200	Zero-point correction= 0.342097 (Hartree/Particle)<br>Thermal correction to Energy= 0.364430<br>Thermal correction to Enthalpy= 0.365375<br>Thermal correction to Gibbs Free Energy= 0.289095<br>Sum of electronic and zero-point Energies= -1333.866234<br>Sum of electronic and thermal Energies= -1333.843900<br>Sum of electronic and thermal Enthalpies= -1333.842956<br>Sum of electronic and thermal Free Energies= -1333.919236
**Name**	**MB-C4-C2-OH-RAF**
Cartesian Coordinates	Frequency and Energy

C -3.41076400 -1.23651200 -0.31820900<br>C -3.59371000 0.19940800 -0.24351100<br>C -2.37778600 1.10648100 -0.10159600<br>C -2.15889300 -1.76512500 -0.42514400<br>C -1.06134900 0.37753300 -0.19850000<br>S 0.30667700 1.44227300 -0.13540500<br>N 0.19805300 -1.71686200 -0.41063000<br>C -0.94088000 -0.99217800 -0.34530700<br>C 1.63706600 0.28357400 -0.12370700<br>C 2.90575400 0.80785300 0.01222400<br>C 4.04473900 -0.03544800 -0.00799900<br>C 3.82696400 -1.43346200 -0.17971700<br>C 2.55904200 -1.93199500 -0.31067000<br>C 1.40319800 -1.11003400 -0.28663000<br>N 5.29150600 0.46929200 0.12916900<br>C 5.48814400 1.90600100 0.27237100<br>C 6.45154300 -0.41225100 0.06951300<br>N -4.77818500 0.72274000 -0.28015900<br>C -5.97886900 -0.10442800 -0.49902400<br>C -5.05338100 2.14413600 -0.01851800<br>H -4.26843000 -1.88064900 -0.43741200<br>H -2.04575700 -2.83724100 -0.53871600<br>H 3.02387500 1.87733800 0.13013800<br>H 4.66544100 -2.11534400 -0.20593800<br>H 2.40699300 -2.99798600 -0.43635900<br>H 6.55160100 2.10701200 0.36729200<br>H 5.10758300 2.44590300 -0.59941600<br>H 4.98262400 2.28460400 1.16473600<br>H 6.41202300 -1.16821700 0.85780700<br>H 6.51583000 -0.91847700 -0.89759600<br>H 7.34893400 0.18375400 0.20932900<br>H -6.20300500 -0.66514800 0.40993400<br>H -6.80381000 0.56230000 -0.73218900<br>H -5.82247300 -0.78181400 -1.33422800<br>H -5.13032700 2.68571200 -0.96101400<br>H -6.00446900 2.19255400 0.50714900<br>H -4.28411600 2.57921500 0.61278400<br>O -3.61823400 -1.30517000 1.91364600<br>H -2.68919600 -1.07983100 2.09648100<br>H -2.44031200 1.55640700 0.89964700<br>O -2.41881400 2.11503200 -1.09806100<br>H -2.56147900 2.97779700 -0.69068600	Zero-point correction= 0.342229 (Hartree/Particle)<br>Thermal correction to Energy= 0.364626<br>Thermal correction to Enthalpy= 0.365571<br>Thermal correction to Gibbs Free Energy= 0.288034<br>Sum of electronic and zero-point Energies= -1333.869878<br>Sum of electronic and thermal Energies= -1333.847481<br>Sum of electronic and thermal Enthalpies= -1333.846537<br>Sum of electronic and thermal Free Energies= -1333.924074
**Name**	**MB-C4-C3-OH-RAF**
Cartesian Coordinates	Frequency and Energy
C 3.61393800 1.32415300 0.20753300<br>C 3.83648600 -0.12257600 0.14856400<br>C 2.60987800 -1.00754000 -0.12305400<br>C 2.33593900 1.90119000 0.01120900<br>C 1.29950100 -0.28048900 0.04203400<br>S -0.05044900 -1.35444500 0.13543400<br>N -0.00533200 1.81624600 -0.07718500<br>C 1.16477300 1.12164200 -0.02853500<br>C -1.40732200 -0.22588600 0.06694400<br>C -2.66461300 -0.77575700 0.11152900	Zero-point correction= 0.344543 (Hartree/Particle)<br>Thermal correction to Energy= 0.365788<br>Thermal correction to Enthalpy= 0.366732<br>Thermal correction to Gibbs Free Energy= 0.293536<br>Sum of electronic and zero-point Energies= -1333.869464<br>Sum of electronic and thermal Energies= -1333.848219<br>Sum of electronic and thermal Enthalpies= -1333.847275<br>Sum of electronic and thermal Free Energies= -1333.920471

C -3.82282600 0.04894400 0.04701800	
C -3.62645000 1.45804500 -0.06176200	
C -2.36634800 1.98219300 -0.10227200	
C -1.19032000 1.18092000 -0.04089400	
N -5.05804000 -0.48580100 0.08531500	
C -5.23707400 -1.93096600 0.18602400	
C -6.23528700 0.37564800 0.02273300	
N 5.05212600 -0.64757500 -0.18221900	
C 6.22052700 0.23871900 -0.12589900	
C 5.35003500 -1.97745900 0.36871600	
H 4.47224400 1.97958000 0.23194900	
H 2.24899600 2.97885600 -0.00307200	
H -2.77010300 -1.85011400 0.19314500	
H -4.47745700 2.12273400 -0.11242200	
H -2.22757600 3.05410900 -0.18377400	
H -6.30032800 -2.15188100 0.18548400	
H -4.77297300 -2.44050100 -0.66168700	
H -4.79995900 -2.31513800 1.11087800	
H -6.24974700 1.07728800 0.86005000	
H -6.25596800 0.94120900 -0.91179100	
H -7.12399400 -0.24611900 0.07415500	
H 6.41536900 0.59051200 0.89262700	
H 7.08298100 -0.32992900 -0.46811100	
H 6.09332400 1.08716300 -0.79560400	
H 6.23567100 -2.36447000 -0.13134900	
H 5.54609000 -1.91643400 1.44442500	
H 4.53613200 -2.67839400 0.19715000	
O 3.77603500 0.41782100 1.66508800	
H 2.88594800 0.30682400 2.04886000	
H 2.63762700 -1.85209800 0.57352000	
O 2.65753100 -1.46602000 -1.46304000	
H 3.24986500 -2.22658300 -1.52372900	
**Name**	**MB-C4-C5-OH-RAF**
Cartesian Coordinates	Frequency and Energy
C 3.52660200 1.41956600 -0.53666000	Zero-point correction= 0.342940 (Hartree/Particle)
C 3.68881900 -0.01189200 -0.33858000	Thermal correction to Energy= 0.365130
C 2.56477700 -0.89768300 -0.84415100	Thermal correction to Enthalpy= 0.366074
C 2.29643800 1.93173500 -0.69929200	Thermal correction to Gibbs Free Energy= 0.291162
C 1.23687900 -0.25698500 -0.54905300	Sum of electronic and zero-point Energies= -1333.864907
S -0.10709300 -1.34170600 -0.70860100	Sum of electronic and thermal Energies= -1333.842717
N -0.06062100 1.83598700 -0.53318500	Sum of electronic and thermal Enthalpies= -1333.841772
C 1.07385600 1.13490500 -0.60573500	Sum of electronic and thermal Free Energies= -1333.916684
C -1.43853900 -0.22941800 -0.36499900	
C -2.67510400 -0.79348400 -0.14882000	
C -3.81024400 0.02859500 0.09446500	
C -3.63287700 1.44077300 0.11973000	
C -2.39134000 1.97508100 -0.08548400	
C -1.24854500 1.17546700 -0.33784100	
N -5.02546000 -0.52666700 0.29598300	
C -5.19824100 -1.97838200 0.32410300	
C -6.20076400 0.31819100 0.50110500	
N 4.77855500 -0.52446200 0.12900000	
C 5.87087900 0.29124200 0.68304800	
C 5.01954000 -1.97430200 0.22578100	

H 4.39745400 2.05948500 -0.57190500<br>H 2.16420900 2.99552000 -0.85989700<br>H -2.78009300 -1.87063700 -0.17390500<br>H -4.47016700 2.09669900 0.31143700<br>H -2.25026100 3.04857700 -0.05916800<br>H -6.23681000 -2.19854500 0.54618000<br>H -4.93606000 -2.40504800 -0.64623400<br>H -4.56112500 -2.41541400 1.09461300<br>H -6.09373500 0.88600200 1.42796800<br>H -6.31105500 1.01139900 -0.33375300<br>H -7.07758400 -0.31677600 0.56339600<br>H 5.50668500 1.26158700 1.00256600<br>H 6.26520600 -0.24411300 1.54503700<br>H 6.65544100 0.39685300 -0.06724600<br>H 6.09599400 -2.12551600 0.19115000<br>H 4.63112400 -2.33599100 1.17905100<br>H 4.56318100 -2.50913400 -0.60105000<br>O 1.95788100 -0.36917400 1.65641600<br>H 1.03343400 -0.35654000 1.92665500<br>H 2.59497600 -1.87005300 -0.35653700<br>O 2.68566000 -1.02862900 -2.25522100<br>H 3.38100100 -1.66903600 -2.45539300	
**Name**	**MB-C4-C7-OH-RAF**
Cartesian Coordinates	Frequency and Energy
C 3.55222400 1.46761600 -0.19622600<br>C 3.74532300 0.06438200 -0.10051700<br>C 2.58406200 -0.77217100 -0.09541400<br>C 2.28241500 1.98955300 -0.25758800<br>C 1.28635500 -0.21236300 -0.05331100<br>S -0.03017600 -1.30118300 -0.52124300<br>N -0.08808200 1.83569100 -0.22218400<br>C 1.10194100 1.20788800 -0.21767900<br>C -1.38641500 -0.23977800 -0.22912500<br>C -2.71431100 -0.94557100 -0.16894400<br>C -3.90655800 -0.00491600 -0.04539500<br>C -3.69632200 1.41111100 0.08655000<br>C -2.44770300 1.91605600 0.03125800<br>C -1.23845600 1.11967900 -0.13630700<br>N -5.10503100 -0.50999800 -0.03667900<br>C -5.40632500 -1.93212800 -0.24554200<br>C -6.28781200 0.33587400 0.20606300<br>N 4.96620000 -0.49231300 -0.04518900<br>C 6.15185400 0.35493100 -0.14220300<br>C 5.13886900 -1.93633600 0.11839900<br>H 4.40244500 2.13359400 -0.22422700<br>H 2.15415400 3.06358900 -0.33593500<br>H 2.68252400 -1.84927000 -0.10609400<br>H -2.83909100 -1.50269900 -1.10599700<br>H -4.54027700 2.07434500 0.20540400<br>H -2.30283100 2.98826900 0.10816000<br>H -5.58547800 -2.40794800 0.71939800<br>H -6.31073300 -1.98637400 -0.84838000<br>H -4.60183800 -2.43887000 -0.76796300<br>H -6.14503800 0.92512000 1.10950500	Zero-point correction= 0.343240 (Hartree/Particle)<br>Thermal correction to Energy= 0.365149<br>Thermal correction to Enthalpy= 0.366094<br>Thermal correction to Gibbs Free Energy= 0.292126<br>Sum of electronic and zero-point Energies= -1333.856808<br>Sum of electronic and thermal Energies= -1333.834899<br>Sum of electronic and thermal Enthalpies= -1333.833955<br>Sum of electronic and thermal Free Energies= -1333.907922

H -6.45470100 0.99222200 -0.64841800		
H -7.14491700 -0.31747100 0.33758800		
H 6.20177000 1.04120800 0.70679100		
H 7.03499000 -0.27629300 -0.14278600		
H 6.12827500 0.93638300 -1.06592600		
H 4.80982300 -2.47128400 -0.77525000		
H 6.19232700 -2.14079300 0.28325900		
H 4.57359800 -2.29184900 0.98077300		
O 1.30258500 -0.51131200 1.87387900		
H 1.89432400 0.19707400 2.17308100		
O -2.75152100 -1.83779000 0.93813900		
H -2.79175300 -2.74737000 0.61934200		
**Name**	**MB-C4-C8-OH-RAF**	
Cartesian Coordinates	Frequency and Energy	
C 3.62404000 1.57789600 -0.01188400	Zero-point correction=	0.342184 (Hartree/Particle)
C 3.81110200 0.17942200 -0.21179900	Thermal correction to Energy=	0.363967
C 2.64583600 -0.64581400 -0.24942200	Thermal correction to Enthalpy=	0.364911
C 2.36856700 2.10838400 0.09931800	Thermal correction to Gibbs Free Energy=	0.291067
C 1.37506300 -0.07139700 -0.18825700	Sum of electronic and zero-point Energies=	-1333.876634
S 0.02947900 -1.18513200 -0.30575700	Sum of electronic and thermal Energies=	-1333.854851
N 0.00226900 1.95915500 0.13159400	Sum of electronic and thermal Enthalpies=	-1333.853907
C 1.19033400 1.31786100 0.01842100	Sum of electronic and thermal Free Energies=	-1333.927750
C -1.30714500 -0.08758500 -0.15017800		
C -2.65051800 -0.74343500 -0.26698900		
C -3.83020300 0.21546100 -0.11593700		
C -3.60297200 1.61372300 0.09793500		
C -2.33838800 2.09138000 0.16552400		
C -1.13989100 1.27482800 0.04498400		
N -5.03853300 -0.27895200 -0.17474400		
C -5.35225400 -1.68631000 -0.47195000		
C -6.21882600 0.56698500 0.07365000		
N 5.03616200 -0.36396800 -0.32130600		
C 6.22188700 0.48469000 -0.32205400		
C 5.19896200 -1.81453800 -0.37698500		
H 4.47950700 2.23420300 0.05944000		
H 2.22954600 3.17105000 0.25726600		
H 2.73186800 -1.68443100 -0.53197000		
H -2.73077900 -1.18928000 -1.26865300		
H -4.43688100 2.29361700 0.19142100		
H -2.15872900 3.15056700 0.31630200		
H -6.20210600 -1.69493400 -1.15337000		
H -4.51105400 -2.19764700 -0.92480900		
H -5.61319500 -2.19843500 0.45490400		
H -6.09546600 1.11949800 1.00382800		
H -6.36819000 1.25904600 -0.75686700		
H -7.08725900 -0.07970300 0.16233300		
H 6.37569500 0.95256700 0.65484000		
H 7.08953400 -0.12646000 -0.55455400		
H 6.13971500 1.26293300 -1.08340200		
H 4.87359500 -2.21074400 -1.34383100		
H 6.25075900 -2.05323800 -0.24404000		
H 4.62770600 -2.28126600 0.42736300		
O 2.74908300 -1.43573100 1.76788500		
H 2.75733500 -0.59191500 2.24709000		

O -2.80301300 -1.82352800 0.63892300<br>H -2.36468000 -1.60217900 1.46928600	
**Name**	**MB-C4-C9-OH-RAF**
Cartesian Coordinates	Frequency and Energy
C 3.39871800 1.48297700 -0.23740200<br>C 3.62226200 0.04961400 -0.13758400<br>C 2.43328700 -0.77145900 -0.10665700<br>C 2.14423600 2.00035500 -0.19322300<br>C 1.16389000 -0.19761800 -0.05897500<br>S -0.16676500 -1.32303200 0.06445700<br>N -0.24418900 1.83881200 -0.06046600<br>C 0.97444200 1.19057200 -0.09847100<br>C -1.51172600 -0.25543400 -0.01766000<br>C -2.83945000 -0.96446800 -0.05009000<br>C -4.03214300 -0.01884900 -0.00657500<br>C -3.82820800 1.41293500 0.01655900<br>C -2.59199200 1.93456300 -0.01393500<br>C -1.36309000 1.13882700 -0.03224400<br>N -5.22936000 -0.51697900 0.00185700<br>C -5.52845900 -1.94744400 -0.16234300<br>C -6.42489200 0.33269200 0.16633000<br>N 4.81085400 -0.48073900 -0.58253400<br>C 6.01688300 0.30615700 -0.31182500<br>C 5.01123700 -1.91629300 -0.38188600<br>H 4.24531600 2.14890400 -0.31455800<br>H 1.99766200 3.07306700 -0.24468400<br>H 2.53091300 -1.84774700 -0.06966000<br>H -2.90647400 -1.51755200 -0.99637800<br>H -4.68376900 2.07262800 0.02258000<br>H -2.45795600 3.01005300 -0.02485900<br>H -6.44465900 -2.01706500 -0.74494200<br>H -4.73300400 -2.45915600 -0.69463600<br>H -5.68576300 -2.39850900 0.81781300<br>H -6.26801100 1.04954700 0.96772300<br>H -6.63916600 0.84814900 -0.77050900<br>H -7.25645100 -0.31580500 0.42591800<br>H 6.15556600 0.46804100 0.76230500<br>H 6.87405600 -0.23980700 -0.69932900<br>H 5.98420900 1.26693900 -0.82073200<br>H 4.26154800 -2.49258100 -0.92167900<br>H 5.98444500 -2.18596500 -0.78614400<br>H 4.98154000 -2.18416000 0.67961700<br>O 3.72134300 -0.19634300 1.77456500<br>H 3.08904500 0.46124100 2.09596600<br>O -2.91660300 -1.86515900 1.04514800<br>H -3.04811700 -2.76520200 0.72334600	Zero-point correction= 0.343610 (Hartree/Particle)<br>Thermal correction to Energy= 0.365256<br>Thermal correction to Enthalpy= 0.366200<br>Thermal correction to Gibbs Free Energy= 0.293136<br>Sum of electronic and zero-point Energies= -1333.858001<br>Sum of electronic and thermal Energies= -1333.836355<br>Sum of electronic and thermal Enthalpies= -1333.835411<br>Sum of electronic and thermal Free Energies= -1333.908475
**Name**	**MB-C4-C10-OH-RAF**
Cartesian Coordinates	Frequency and Energy
C -3.44206700 -1.21471900 -0.37844300<br>C -3.57702000 0.22040800 -0.18399900<br>C -2.40226900 0.97874100 -0.02754600<br>C -2.20330200 -1.79555600 -0.51499300<br>C -1.16194100 0.36831000 -0.10649100<br>S 0.23473400 1.34620100 0.10530500	Zero-point correction= 0.342073 (Hartree/Particle)<br>Thermal correction to Energy= 0.364337<br>Thermal correction to Enthalpy= 0.365281<br>Thermal correction to Gibbs Free Energy= 0.290033<br>Sum of electronic and zero-point Energies= -1333.845121<br>Sum of electronic and thermal Energies= -1333.822857

N 0.14479500 -1.70029300 -0.42430700<br>C -0.99735000 -1.04767600 -0.34909300<br>C 1.52389100 0.28279000 -0.10558700<br>C 2.87941800 0.96510600 -0.06915700<br>C 4.03123800 -0.01730500 0.02558000<br>C 3.80113900 -1.37955300 -0.14118000<br>C 2.52742200 -1.90522800 -0.33927600<br>C 1.36323300 -1.09817000 -0.30199300<br>N 5.25914200 0.49643400 0.24291300<br>C 5.59335500 1.90440700 0.03741900<br>C 6.38501300 -0.39374400 0.49377100<br>N -4.79291800 0.77305500 -0.13107300<br>C -6.00101400 -0.03958000 -0.28376700<br>C -4.92628300 2.21189100 0.08111400<br>H -4.31687700 -1.80242500 -0.61612200<br>H -2.11686300 -2.85563300 -0.71948200<br>H -2.45728400 2.04563400 0.14383700<br>H 2.97756800 1.51781800 -1.01478100<br>H 4.64117900 -2.06111200 -0.14856000<br>H 2.39385700 -2.96804700 -0.49390900<br>H 6.58364500 1.95448600 -0.41528600<br>H 4.89700700 2.37989500 -0.65100000<br>H 5.61095300 2.45466700 0.98115600<br>H 6.10556500 -1.16356700 1.21319300<br>H 6.72866400 -0.87328000 -0.42854900<br>H 7.20017400 0.19316100 0.91249800<br>H -6.03217000 -0.83073800 0.46744900<br>H -6.86561100 0.60301700 -0.14842200<br>H -6.04804300 -0.48564500 -1.27943200<br>H -4.42310600 2.76640700 -0.71445600<br>H -5.98064500 2.47015400 0.07703400<br>H -4.49419900 2.50112700 1.04219300<br>O -3.60761800 -1.67318100 1.70993600<br>H -3.59484700 -2.63995100 1.60920400<br>O 2.87114700 1.88666500 1.01229000<br>H 3.16408600 2.75313500 0.70586400	Sum of electronic and thermal Enthalpies= -1333.821913<br>Sum of electronic and thermal Free Energies= -1333.897160
**Name**	**MB-C4-C11-OH-RAF**
Cartesian Coordinates	Frequency and Energy
C 3.51685100 1.17474200 -0.34019300<br>C 3.64474400 -0.25884300 -0.22208600<br>C 2.46806000 -1.01904400 -0.07876800<br>C 2.28980400 1.79762700 -0.22361400<br>C 1.22892000 -0.39456600 -0.07099100<br>S -0.17611300 -1.38090500 0.10263900<br>N -0.06913800 1.69311200 -0.24531900<br>C 1.05970400 1.03003000 -0.18655100<br>C -1.46329900 -0.29195800 0.00746700<br>C -2.82025000 -0.93686300 0.24192200<br>C -3.98022000 -0.02302000 -0.09078700<br>C -3.74869500 1.33678400 -0.29572000<br>C -2.46637600 1.87037700 -0.32167000<br>C -1.29823800 1.07577200 -0.19089400<br>N -5.21087900 -0.56974400 -0.13541600<br>C -5.50176400 -1.93312700 0.30141000	Zero-point correction= 0.342960 (Hartree/Particle)<br>Thermal correction to Energy= 0.365117<br>Thermal correction to Enthalpy= 0.366062<br>Thermal correction to Gibbs Free Energy= 0.291028<br>Sum of electronic and zero-point Energies= -1333.840573<br>Sum of electronic and thermal Energies= -1333.818416<br>Sum of electronic and thermal Enthalpies= -1333.817471<br>Sum of electronic and thermal Free Energies= -1333.892505

C	-6.36896700	0.30603700	-0.28896400	
N	4.86147700	-0.82449100	-0.26040400	
C	6.07549100	-0.01499600	-0.37988900	
C	4.98405500	-2.27497800	-0.16385800	
H	4.39604000	1.78715900	-0.48206200	
H	2.20095500	2.85443400	-0.43467700	
H	2.51912600	-2.09563000	0.01530800	
H	-2.87468400	-1.83239400	-0.38336200	
H	-4.58238000	2.00918600	-0.44235800	
H	-2.32856600	2.93476300	-0.46505600	
H	-6.37664800	-2.28667700	-0.24122700	
H	-4.67799900	-2.60804700	0.08285600	
H	-5.71382400	-1.96073200	1.37424400	
H	-6.44909900	0.99692800	0.55553400	
H	-6.29333800	0.88168100	-1.21247800	
H	-7.26521200	-0.30703600	-0.33091900	
H	6.15313800	0.69175600	0.44821600	
H	6.93282200	-0.68035300	-0.35182800	
H	6.08849400	0.53294600	-1.32385900	
H	4.44627300	-2.76056700	-0.98183900	
H	6.03406300	-2.54346700	-0.22326000	
H	4.57835400	-2.63239500	0.78604500	
O	2.65735300	2.16962000	1.75665200	
H	2.67426500	1.25473600	2.08339100	
O	-2.89405700	-1.30461600	1.61932200	
H	-3.00025700	-2.26150200	1.68887600	
**Name**				**MB-C4-C12-OH-RAF**
Cartesian Coordinates				Frequency and Energy
C	3.65522000	1.37285100	0.06581600	Zero-point correction= 0.343073 (Hartree/Particle)
C	3.83852300	-0.04077100	0.00329600	Thermal correction to Energy= 0.365219
C	2.67532100	-0.86303500	0.08312000	Thermal correction to Enthalpy= 0.366163
C	2.41154700	1.90566500	0.23668500	Thermal correction to Gibbs Free Energy= 0.291416
C	1.43768800	-0.30690000	0.25506300	Sum of electronic and zero-point Energies= -1333.864765
S	0.07369800	-1.41961800	0.42272600	Sum of electronic and thermal Energies= -1333.842619
N	0.02049300	1.76189800	0.30629400	Sum of electronic and thermal Enthalpies= -1333.841675
C	1.24629500	1.10064700	0.43022100	Sum of electronic and thermal Free Energies= -1333.916422
C	-1.24755500	-0.32556500	0.23789800	
C	-2.58086800	-1.01643100	0.11672000	
C	-3.76367400	-0.07002700	-0.04145700	
C	-3.55514200	1.35812500	-0.03810000	
C	-2.31689700	1.86660800	0.07301400	
C	-1.09812100	1.06654500	0.21162100	
N	-4.95243100	-0.57069800	-0.17652900	
C	-5.24418100	-2.00763400	-0.28135000	
C	-6.14777300	0.29174600	-0.23909800	
N	5.06105300	-0.58627800	-0.14084600	
C	6.24065000	0.26842200	-0.23797600	
C	5.21846200	-2.03195200	-0.27145600	
H	4.50154000	2.03572800	-0.04756900	
H	2.27542400	2.98092500	0.25764000	
H	2.76716800	-1.93873500	0.00432100	
H	-2.55117500	-1.64059000	-0.78407600	
H	-4.39954800	2.02329100	-0.14537200	
H	-2.17304800	2.94111800	0.06066800	

H -5.66125400 -2.35117700 0.66588100<br>H -5.98776500 -2.12627700 -1.06762200<br>H -4.35761400 -2.58056300 -0.52523700<br>H -6.14718000 0.98868700 0.59611200<br>H -6.16016100 0.83438100 -1.18443300<br>H -7.02199600 -0.34838400 -0.17253600<br>H 6.31624900 0.92134400 0.63373500<br>H 7.12420400 -0.36163900 -0.27766100<br>H 6.20580400 0.88547100 -1.13955200<br>H 4.69524800 -2.40368300 -1.15604700<br>H 6.27574700 -2.26005000 -0.36886600<br>H 4.82986800 -2.54564400 0.61068900<br>O 1.14177100 1.08554100 2.47387000<br>H 1.06459500 2.04356700 2.59298500<br>O -2.79238700 -1.90762100 1.19772100<br>H -2.75021900 -1.40913600 2.02607700	
**Name**	**MB-C4-C14-OH-RAF**
Cartesian Coordinates	Frequency and Energy
C -3.54391900 -1.31732500 -0.37292000<br>C -3.74667700 0.09953500 -0.19629200<br>C -2.56068300 1.02149000 0.05992500<br>C -2.31173300 -1.84412900 -0.29005600<br>C -1.23323900 0.31814500 0.05813000<br>S 0.11168500 1.38485900 0.29396300<br>N 0.05880700 -1.76955400 -0.15548200<br>C -1.09653000 -1.06939000 -0.01973300<br>C 1.46185600 0.26727600 0.05001200<br>C 2.71909800 0.81745100 0.05644200<br>C 3.87423100 0.00153600 -0.10968600<br>C 3.67618000 -1.40151300 -0.28280500<br>C 2.41681300 -1.92762600 -0.28745600<br>C 1.24501300 -1.13464800 -0.12399300<br>N 5.10757200 0.53870800 -0.10410400<br>C 5.28840200 1.97775800 0.06370600<br>C 6.28320300 -0.31205900 -0.27035600<br>N -4.93816700 0.61360600 -0.23858700<br>C -6.14229200 -0.23181900 -0.34634100<br>C -5.21708700 2.05535400 -0.18053500<br>H -4.38624800 -1.95814200 -0.58802000<br>H -2.16674000 -2.90983000 -0.42694800<br>H -2.54168800 1.75504700 -0.75619800<br>H 2.82875500 1.88568200 0.19312400<br>H 4.52495100 -2.05833200 -0.41201200<br>H 2.27451000 -2.99376300 -0.42158100<br>H 6.35030900 2.20211200 0.03101000<br>H 4.78872700 2.52847600 -0.73670700<br>H 4.88922200 2.31129300 1.02452300<br>H 6.34163400 -1.05544000 0.52797200<br>H 6.25783500 -0.82832700 -1.23272400<br>H 7.17154600 0.31123800 -0.23207400<br>H -6.07809100 -1.06243200 0.35221700<br>H -7.00124600 0.38274500 -0.09387300<br>H -6.24187100 -0.60101100 -1.36757000<br>H -4.36545000 2.63880000 -0.51785200	Zero-point correction= 0.342161 (Hartree/Particle)<br>Thermal correction to Energy= 0.364621<br>Thermal correction to Enthalpy= 0.365565<br>Thermal correction to Gibbs Free Energy= 0.289593<br>Sum of electronic and zero-point Energies= -1333.866621<br>Sum of electronic and thermal Energies= -1333.844162<br>Sum of electronic and thermal Enthalpies= -1333.843217<br>Sum of electronic and thermal Free Energies= -1333.919189

H -6.05737900 2.24864200 -0.84386600	
H -5.48966000 2.33032300 0.83899400	
O -1.46748900 -1.28574700 2.14642500	
H -0.54632000 -1.09195200 2.38570700	
O -2.74150000 1.68164500 1.30560400	
H -2.95200500 2.61142200 1.15663000	
**Name**	**MB-C4-N13-OH-RAF**
Cartesian Coordinates	Frequency and Energy
C 3.60765700 1.18321900 -0.01920800	Zero-point correction= 0.341673 (Hartree/Particle)
C 3.76383400 -0.23192300 0.09384500	Thermal correction to Energy= 0.364027
C 2.65293500 -1.02406100 0.75505700	Thermal correction to Enthalpy= 0.364971
C 2.38221800 1.76796500 0.10878400	Thermal correction to Gibbs Free Energy= 0.289354
C 1.33502900 -0.42215300 0.40871100	Sum of electronic and zero-point Energies= -1333.841876
S -0.01248600 -1.49254400 0.44204500	Sum of electronic and thermal Energies= -1333.819522
N 0.01766300 1.60865100 0.19359800	Sum of electronic and thermal Enthalpies= -1333.818577
C 1.19908700 0.97874300 0.25535000	Sum of electronic and thermal Free Energies= -1333.894195
C -1.35874300 -0.42204200 0.14870600	
C -2.60370700 -1.00156700 -0.01950700	
C -3.75644400 -0.19977900 -0.18199800	
C -3.58942800 1.22265500 -0.17435800	
C -2.35517400 1.78170200 -0.02211000	
C -1.19427100 0.98780700 0.13614700	
N -4.97446700 -0.75012600 -0.33896100	
C -5.12146600 -2.20042100 -0.37731900	
C -6.15179600 0.09375000 -0.52269600	
N 4.86887500 -0.83280300 -0.29876500	
C 5.91662700 -0.08877400 -1.00659500	
C 5.19527400 -2.24095500 -0.05901900	
H 4.47356000 1.80052200 -0.21513400	
H 2.27206400 2.84347900 0.05608800	
H 2.67211300 -2.06058500 0.41421800	
H -2.68447000 -2.08012800 -0.01144600	
H -4.44499200 1.87003500 -0.30353200	
H -2.23872100 2.85805600 -0.03290000	
H -6.17144600 -2.44235800 -0.51411200	
H -4.55142000 -2.63047700 -1.20552700	
H -4.77798700 -2.65332000 0.55630400	
H -6.29060200 0.76402900 0.32840800	
H -6.07072100 0.69241000 -1.43360100	
H -7.02604300 -0.54549100 -0.60378100	
H 6.49395700 0.51453900 -0.30273500	
H 6.57541900 -0.80891600 -1.48549000	
H 5.47421500 0.55286300 -1.76648800	
H 4.53906400 -2.69509000 0.67556600	
H 5.13616000 -2.79168600 -0.99933300	
H 6.21874300 -2.28542500 0.31371600	
O -0.04299800 2.97153500 1.31278000	
H -0.08838800 3.69021700 0.66420900	
O 2.81341300 -0.98443100 2.18321600	
H 3.56804700 -1.53571200 2.43224500	
**Name**	**MB-C4-C16-OH-FHT**
Cartesian Coordinates	Frequency and Energy
C -3.30963000 -1.17835900 -0.36143800	Zero-point correction= 0.337732 (Hartree/Particle)
C -3.46898100 0.24197200 -0.26423500	Thermal correction to Energy= 0.360001

C -2.24887000 1.13469500 -0.08257300	Thermal correction to Enthalpy= 0.360945
C -2.07491300 -1.72504400 -0.36669100	Thermal correction to Gibbs Free Energy= 0.284463
C -0.93828100 0.39378700 -0.10650700	Sum of electronic and zero-point Energies= -1333.861370
S 0.43649600 1.42839300 0.10256600	Sum of electronic and thermal Energies= -1333.839101
N 0.28943900 -1.71492200 -0.29506600	Sum of electronic and thermal Enthalpies= -1333.838157
C -0.83440600 -0.97223900 -0.25416500	Sum of electronic and thermal Free Energies= -1333.914639
C 1.75697100 0.27095400 -0.04041300	
C 3.03721400 0.78772000 0.04241600	
C 4.16593200 -0.05873000 -0.03682200	
C 3.92984800 -1.45518400 -0.18027500	
C 2.65341500 -1.94664600 -0.26286400	
C 1.50712300 -1.11708800 -0.20201800	
N 5.43015300 0.44110100 0.01741400	
C 5.63055500 1.84219600 0.36544300	
C 6.55407200 -0.47280800 0.19122100	
N -4.65427200 0.79733500 -0.29951000	
C -5.85865700 -0.01037200 -0.31833900	
C -4.88055600 2.25007500 -0.20224800	
H -4.17470000 -1.82092900 -0.43336500	
H -1.96962700 -2.80102100 -0.45276400	
H -2.24517700 1.84436700 -0.92031100	
H 3.16284500 1.85435200 0.17531400	
H 4.76025400 -2.14571100 -0.22584800	
H 2.49048000 -3.01248000 -0.37572800	
H 6.69561100 2.05823900 0.34449100	
H 5.13963300 2.49558000 -0.35814200	
H 5.24264500 2.07076100 1.36442100	
H 6.47320500 -1.04026700 1.12454100	
H 6.61749400 -1.17494100 -0.64126800	
H 7.47253700 0.10774600 0.21239800	
H -5.95032100 -0.55142100 0.71151000	
H -6.73285600 0.62694700 -0.39984700	
H -5.83964100 -0.77446300 -1.09116000	
H -4.05023500 2.80283800 -0.63316900	
H -5.77942200 2.47926000 -0.76779300	
H -5.02000500 2.52846800 0.84230600	
O -5.85806100 -1.27003100 1.94020900	
H -5.32042700 -2.01953400 1.62675800	
O -2.37534400 1.82920300 1.15025100	
H -2.43426900 2.77886300 0.98971800	
**Name**	**MB-C4-C17-OH-FHT**
Cartesian Coordinates	Frequency and Energy
C -3.29265300 -1.78170900 -0.13261000	Zero-point correction= 0.337587 (Hartree/Particle)
C -3.56390000 -0.38541700 -0.30137300	Thermal correction to Energy= 0.359988
C -2.42817800 0.57003400 -0.63522300	Thermal correction to Enthalpy= 0.360932
C -2.01580900 -2.21344200 -0.05734300	Thermal correction to Gibbs Free Energy= 0.284372
C -1.06708900 0.00152400 -0.33205700	Sum of electronic and zero-point Energies= -1333.860567
S 0.21433100 1.16533700 -0.41603800	Sum of electronic and thermal Energies= -1333.838165
N 0.32876500 -1.96105300 0.09713100	Sum of electronic and thermal Enthalpies= -1333.837221
C -0.84723200 -1.34274600 -0.10309100	Sum of electronic and thermal Free Energies= -1333.913781
C 1.62281700 0.14820000 -0.11891100	
C 2.85061800 0.78422800 -0.10351500	
C 4.03930200 0.05580600 0.13213200	
C 3.92098700 -1.34927000 0.32625000	

C 2.69400700 -1.95909000 0.30755000<br>C 1.48876400 -1.24919000 0.09039000<br>N 5.24881700 0.67455800 0.17637600<br>C 5.35452400 2.07013500 -0.22992500<br>C 6.46650200 -0.12829200 0.20002800<br>N -4.78070400 0.08397000 -0.22190000<br>C -5.90653300 -0.79104600 0.16160000<br>C -5.09648700 1.48359900 -0.40339700<br>H -4.10629200 -2.48923300 -0.06433300<br>H -1.81437300 -3.27164900 0.06741400<br>H 2.89070300 1.85177200 -0.27621300<br>H 4.80268100 -1.95241500 0.49128200<br>H 2.61987400 -3.02968900 0.46101700<br>H 6.38745600 2.38909900 -0.11839600<br>H 5.05188400 2.21086900 -1.27343200<br>H 4.73367700 2.70697000 0.40268400<br>H 6.48984400 -0.77320100 1.07962300<br>H 6.55712700 -0.75214200 -0.69558100<br>H 7.32217900 0.54012500 0.24994200<br>H -5.64938800 -1.34797800 1.06040700<br>H -6.76805900 -0.16165600 0.35915400<br>H -6.13163800 -1.47594900 -0.65597400<br>H -4.44774500 1.97432100 -1.11897400<br>H -6.14304900 1.59621400 -0.67220800<br>H -4.97096000 2.01477300 0.62241300<br>O -4.47357300 2.72603100 1.78357000<br>H -3.65554400 3.06978300 1.38103100<br>H -2.57042200 1.48005600 -0.04148100<br>O -2.54709300 0.86699700 -2.02620500<br>H -2.42156400 1.81450000 -2.16334100	
**Name**	**MB-C4-C19-OH-FHT**
Cartesian Coordinates	Frequency and Energy
C 3.69866200 1.34850700 0.02617000<br>C 3.86530500 -0.05025100 -0.13024000<br>C 2.72502700 -0.87060900 -0.20095800<br>C 2.43953700 1.87917400 0.12821000<br>C 1.45726400 -0.30665900 -0.08884400<br>S 0.11370000 -1.43017500 -0.16005800<br>N 0.06425400 1.72011800 0.21776600<br>C 1.27722100 1.07704000 0.08407600<br>C -1.21842700 -0.34983700 -0.02637600<br>C -2.55511100 -1.03845800 -0.11674000<br>C -3.74370200 -0.09908100 0.02985000<br>C -3.52982400 1.31250700 0.26634600<br>C -2.28825900 1.81816700 0.30980800<br>C -1.06340300 1.02442300 0.15790400<br>N -4.94151700 -0.58532500 -0.05138800<br>C -5.25220800 -1.98275000 -0.38710800<br>C -6.13298700 0.25125200 0.19380500<br>N 5.11761200 -0.61539300 -0.17711900<br>C 6.29606500 0.22137400 0.02045100<br>C 5.26819100 -2.04126900 -0.18571100<br>H 4.55676300 2.00377800 0.07494500<br>H 2.30714200 2.94671200 0.25478800	Zero-point correction= 0.341016 (Hartree/Particle)<br>Thermal correction to Energy= 0.362972<br>Thermal correction to Enthalpy= 0.363916<br>Thermal correction to Gibbs Free Energy= 0.289373<br>Sum of electronic and zero-point Energies= -1333.868637<br>Sum of electronic and thermal Energies= -1333.846681<br>Sum of electronic and thermal Enthalpies= -1333.845736<br>Sum of electronic and thermal Free Energies= -1333.920279

H 2.81689100 -1.93986300 -0.33631900<br>H -2.62784100 -1.49774100 -1.10953100<br>H -4.37921900 1.97028700 0.38181500<br>H -2.14189000 2.88076600 0.46611700<br>H -6.08095300 -1.96318200 -1.09331500<br>H -4.40225000 -2.48814000 -0.82924300<br>H -5.56291500 -2.49927800 0.52166600<br>H -6.26303500 0.95342800 -0.62982200<br>H -6.99362700 -0.40782600 0.25240500<br>H -6.02296400 0.78571800 1.13444200<br>H 6.24354600 0.71626700 0.99303300<br>H 7.17939300 -0.40597400 -0.02754600<br>H 6.34514700 0.97988600 -0.76313000<br>H 4.74671200 -2.48884000 -1.03473200<br>H 6.32177900 -2.29782900 -0.21291000<br>H 4.76709000 -2.43247800 0.75903200<br>O 3.75385800 -2.72113400 2.18201700<br>H 3.10518800 -2.00925600 2.17231800<br>O -2.64442600 -2.10287800 0.81217500<br>H -2.51546300 -1.74955700 1.70407800	
**Name**	**MB-C4-C20-OH-FHT**
Cartesian Coordinates	Frequency and Energy
C -3.33558900 -1.18182400 -0.59702800<br>C -3.47631800 0.22053800 -0.43673800<br>C -2.31145500 0.97304000 -0.21123100<br>C -2.09580100 -1.77267800 -0.55225200<br>C -1.06683700 0.35910000 -0.17177400<br>S 0.30953000 1.40643500 0.14724000<br>N 0.26917800 -1.72829200 -0.31810100<br>C -0.91066000 -1.03588600 -0.34232300<br>C 1.61763900 0.28173500 0.01849300<br>C 2.97332500 0.93151400 0.11899700<br>C 4.13208200 -0.05684500 0.11687500<br>C 3.87846500 -1.46864400 -0.02732000<br>C 2.62154400 -1.92123100 -0.17552000<br>C 1.42860300 -1.07527200 -0.16130400<br>N 5.34331400 0.39554900 0.23718100<br>C 5.69081900 1.82380000 0.23863400<br>C 6.49670200 -0.51500100 0.36775100<br>N -4.71331700 0.82965000 -0.49795200<br>C -5.88588200 0.00864600 -0.38381200<br>C -4.83337900 2.19500800 0.00549900<br>H -4.20120900 -1.80891200 -0.75764300<br>H -2.00363300 -2.84527400 -0.67808300<br>H -2.36881400 2.04355700 -0.06579500<br>H 3.10025800 1.56623700 -0.76880900<br>H 4.70549800 -2.16313300 -0.04650300<br>H 2.44536200 -2.98261200 -0.31073900<br>H 6.65026500 1.92183600 -0.26446200<br>H 4.95284100 2.40834500 -0.30339000<br>H 5.78154300 2.17834900 1.26557000<br>H 6.28799800 -1.27830600 1.11362700<br>H 6.71419600 -0.97561900 -0.59647100<br>H 7.34876800 0.07477600 0.69135800	Zero-point correction= 0.339006 (Hartree/Particle)<br>Thermal correction to Energy= 0.361037<br>Thermal correction to Enthalpy= 0.361981<br>Thermal correction to Gibbs Free Energy= 0.286985<br>Sum of electronic and zero-point Energies= -1333.868732<br>Sum of electronic and thermal Energies= -1333.846701<br>Sum of electronic and thermal Enthalpies= -1333.845757<br>Sum of electronic and thermal Free Energies= -1333.920753

H	-5.92263000	-0.50444700	0.62860800	
H	-6.77870700	0.62428000	-0.46295000	
H	-5.91925400	-0.77663400	-1.13756900	
H	-4.22074600	2.87865600	-0.58235900	
H	-5.87001500	2.50827300	-0.08760700	
H	-4.53221900	2.26165600	1.05714300	
O	-5.50690900	-1.26567000	2.05110400	
H	-4.59545200	-1.47271900	1.77610900	
O	3.02163200	1.71777400	1.29797000	
H	3.26936600	2.62444700	1.08178800	
**Name**				**MB-C14-C1-OH-RAF**
Cartesian Coordinates				Frequency and Energy
C	3.52481800	1.13629900	-0.59203200	Zero-point correction= 0.341962 (Hartree/Particle)
C	3.63401100	-0.25823800	-0.19810500	Thermal correction to Energy= 0.364286
C	2.42673100	-1.02794100	-0.02786400	Thermal correction to Enthalpy= 0.365230
C	2.30418900	1.68762300	-0.80904100	Thermal correction to Gibbs Free Energy= 0.290061
C	1.22077100	-0.51471400	-0.36038700	Sum of electronic and zero-point Energies= -1333.859157
S	-0.17490100	-1.53567200	-0.27955800	Sum of electronic and thermal Energies= -1333.836834
N	-0.05262900	1.64289500	-0.38981200	Sum of electronic and thermal Enthalpies= -1333.835890
C	1.03534300	0.87704700	-0.92840000	Sum of electronic and thermal Free Energies= -1333.911058
C	-1.48890300	-0.36528500	-0.25390700	
C	-2.76293900	-0.87436400	-0.17337900	
C	-3.88125700	-0.01720800	0.00460800	
C	-3.62717600	1.38461000	0.15584600	
C	-2.36257500	1.87483700	0.06888700	
C	-1.21919700	1.04560000	-0.20271300	
N	-5.13522800	-0.50754900	0.05301200	
C	-5.37453500	-1.94080300	-0.08784900	
C	-6.26680000	0.38006700	0.31025700	
N	4.81136500	-0.79288200	0.02458600	
C	6.04478500	0.00778300	-0.04520900	
C	4.94682800	-2.21575700	0.36876300	
H	4.41250200	1.74407800	-0.68750800	
H	2.22056300	2.71221200	-1.15263100	
H	2.49085700	-2.02942300	0.37630200	
H	-2.90589900	-1.94693400	-0.21445400	
H	-4.44750900	2.06684100	0.33052000	
H	-2.18409200	2.93960900	0.16399200	
H	-6.44585200	-2.11785800	-0.09415100	
H	-4.95377400	-2.31094500	-1.02485900	
H	-4.92983500	-2.49511100	0.74282100	
H	-6.16015300	0.87748800	1.27695200	
H	-6.35029400	1.13872800	-0.47083000	
H	-7.17771200	-0.21097300	0.31989600	
H	5.96365800	0.87801700	0.60465700	
H	6.86863100	-0.61178000	0.29216800	
H	6.22820800	0.32675700	-1.07173600	
H	4.39374000	-2.82613500	-0.34336500	
H	5.99822700	-2.47868600	0.32181000	
H	4.57241000	-2.39424000	1.37826200	
O	2.48601000	2.34293400	1.26129800	
H	2.32921800	1.48069600	1.68502400	
O	0.83791500	0.65242100	-2.33083000	
H	0.58099500	1.50001100	-2.72323600	

Name	MB-C14-C2-OH-RAF
Cartesian Coordinates	Frequency and Energy
C -3.41026700 -1.18266700 -0.63669500<br>C -3.55372900 0.22454900 -0.24742400<br>C -2.36593800 1.02761600 -0.11855700<br>C -2.20482600 -1.64310500 -1.05563100<br>C -1.15576300 0.54134100 -0.47409300<br>S 0.24071400 1.56120000 -0.35918100<br>N 0.06748800 -1.61281700 -0.36901600<br>C -0.93722500 -0.83851500 -1.05155700<br>C 1.53722800 0.37092500 -0.26934500<br>C 2.81901900 0.85662400 -0.18050900<br>C 3.90955700 -0.01208400 0.09389800<br>C 3.61489400 -1.39108200 0.34865100<br>C 2.34331400 -1.86012900 0.24720900<br>C 1.23652100 -1.03025400 -0.14328800<br>N 5.17368800 0.45010200 0.13941900<br>C 5.45903200 1.85284600 -0.14832600<br>C 6.27375000 -0.43734600 0.51036900<br>N -4.73695300 0.71654300 0.00796900<br>C -5.93947300 -0.12881300 0.06377300<br>C -4.93544000 2.14704900 0.28157900<br>H -4.29536700 -1.77052100 -0.82805100<br>H -2.10859200 -2.65395100 -1.43738200<br>H -2.44262700 2.01809000 0.31041800<br>H 2.98911600 1.92156000 -0.27759200<br>H 4.41120000 -2.07486900 0.60745600<br>H 2.13570400 -2.91094300 0.41249100<br>H 6.53542600 1.99600800 -0.16198800<br>H 5.05668700 2.13299200 -1.12379100<br>H 5.02562200 2.50521900 0.61429100<br>H 6.10998200 -0.86435300 1.50203400<br>H 6.37873700 -1.24943800 -0.21253300<br>H 7.19465800 0.13761000 0.52657700<br>H -5.71366900 -1.07106400 0.55729600<br>H -6.69082000 0.40050600 0.64282300<br>H -6.31881400 -0.30895200 -0.94332800<br>H -4.32705700 2.74627800 -0.39165900<br>H -5.98302400 2.37776700 0.11027100<br>H -4.67893500 2.36823300 1.31934500<br>O -3.31601500 -1.94039500 1.32248600<br>H -2.44107600 -1.58846500 1.56215700<br>O -0.57210200 -0.60764400 -2.41480600<br>H -0.30401000 -1.45899500 -2.79068200	Zero-point correction= 0.341801 (Hartree/Particle)<br>Thermal correction to Energy= 0.364370<br>Thermal correction to Enthalpy= 0.365314<br>Thermal correction to Gibbs Free Energy= 0.288761<br>Sum of electronic and zero-point Energies= -1333.859892<br>Sum of electronic and thermal Energies= -1333.837322<br>Sum of electronic and thermal Enthalpies= -1333.836378<br>Sum of electronic and thermal Free Energies= -1333.912931
**Name**	**MB-C14-C3-OH-RAF**
Cartesian Coordinates	Frequency and Energy
C 3.43185600 1.48181000 -0.49981400<br>C 3.61342900 0.05212400 -0.28291200<br>C 2.43242100 -0.78602100 -0.17841600<br>C 2.21646000 1.98276300 -0.72543100<br>C 1.20855900 -0.28955400 -0.53592000<br>S -0.13694700 -1.35165600 -0.63528200<br>N -0.07182100 1.78659600 -0.10578800<br>C 0.96752700 1.16255300 -0.88404800	Zero-point correction= 0.341959 (Hartree/Particle)<br>Thermal correction to Energy= 0.364395<br>Thermal correction to Enthalpy= 0.365339<br>Thermal correction to Gibbs Free Energy= 0.289528<br>Sum of electronic and zero-point Energies= -1333.862864<br>Sum of electronic and thermal Energies= -1333.840429<br>Sum of electronic and thermal Enthalpies= -1333.839485<br>Sum of electronic and thermal Free Energies= -1333.915296

C	-1.47546800	-0.23032100	-0.36890400	
C	-2.73545500	-0.77428900	-0.36829500	
C	-3.85967500	-0.00481500	0.04328500	
C	-3.61932100	1.32730700	0.51015900	
C	-2.36589100	1.85332200	0.49434900	
C	-1.22389800	1.13754400	-0.00519800	
N	-5.10096400	-0.52405500	0.01913700	
C	-5.32788600	-1.88831600	-0.45068100	
C	-6.23658600	0.25210500	0.51250600	
N	4.80422500	-0.46105200	-0.13299700	
C	6.02852500	0.33337000	-0.31621400	
C	4.99706600	-1.86372700	0.26513200	
H	4.28560500	2.14002700	-0.42550800	
H	2.07313200	3.05175800	-0.84723700	
H	2.54561800	-1.84944600	-0.02243300	
H	-2.86499700	-1.81322000	-0.64508300	
H	-4.44100200	1.93166900	0.86841400	
H	-2.19976600	2.87113500	0.82774300	
H	-6.39679200	-2.07920800	-0.46091200	
H	-4.93951700	-2.01550700	-1.46270300	
H	-4.84470200	-2.61284500	0.20968300	
H	-6.09479200	0.52199100	1.56116500	
H	-6.36593500	1.16391300	-0.07436000	
H	-7.13371800	-0.35276000	0.42408300	
H	6.26583700	0.86861400	0.60497000	
H	6.83622700	-0.35311900	-0.55427200	
H	5.90930100	1.03252500	-1.13886900	
H	4.80837800	-2.52431800	-0.58235000	
H	6.02534400	-1.97973400	0.59377300	
H	4.33139900	-2.11121100	1.08952700	
O	2.25318100	-0.53114200	2.00155300	
H	1.96230600	0.39647600	1.97219600	
O	0.62244400	1.14053100	-2.27323500	
H	0.40814200	2.04956200	-2.52968900	
**Name**				**MB-C14-C4-OH-RAF**
Cartesian Coordinates				Frequency and Energy
C	3.40693400	1.55107600	-0.38093000	Zero-point correction= 0.341801 (Hartree/Particle)
C	3.60164500	0.10883800	-0.30122200	Thermal correction to Energy= 0.364278
C	2.42819300	-0.74579200	-0.27351100	Thermal correction to Enthalpy= 0.365222
C	2.18633300	2.06275700	-0.54519400	Thermal correction to Gibbs Free Energy= 0.289077
C	1.19467900	-0.22647900	-0.56562200	Sum of electronic and zero-point Energies= -1333.861726
S	-0.14170900	-1.28546900	-0.77109500	Sum of electronic and thermal Energies= -1333.839248
N	-0.09148100	1.78043900	0.08479600	Sum of electronic and thermal Enthalpies= -1333.838304
C	0.93994300	1.25373900	-0.76840800	Sum of electronic and thermal Free Energies= -1333.914450
C	-1.48653600	-0.20218900	-0.39843400	
C	-2.74550400	-0.74284600	-0.46973600	
C	-3.87285400	-0.02431000	0.01884500	
C	-3.63365600	1.23729000	0.65218100	
C	-2.38057200	1.76221200	0.70515500	
C	-1.23850200	1.11703700	0.11675500	
N	-5.11592400	-0.52902000	-0.08763000	
C	-5.34317900	-1.81224000	-0.74707100	
C	-6.25417500	0.18308500	0.48903300	
N	4.79478100	-0.40867700	-0.19724600	

C 6.01874200 0.40036500 -0.30024200<br>C 4.99141300 -1.84126200 0.07445600<br>H 4.25482100 2.20722800 -0.24570100<br>H 2.03279200 3.13639000 -0.55845200<br>H 2.55426000 -1.81903900 -0.22714500<br>H -2.87226500 -1.74389100 -0.86319800<br>H -4.45681000 1.79085100 1.08197300<br>H -2.21575600 2.73063100 1.16309800<br>H -6.41285900 -1.98871900 -0.80747400<br>H -4.93269000 -1.80134900 -1.75840600<br>H -4.88117800 -2.62728600 -0.18425500<br>H -6.12245100 0.31626200 1.56485700<br>H -6.37405500 1.16302600 0.02215100<br>H -7.15303000 -0.40069400 0.31518400<br>H 6.27739200 0.80664000 0.67917500<br>H 6.81734400 -0.25152200 -0.64440000<br>H 5.88835500 1.20245100 -1.02011400<br>H 4.78888300 -2.42547400 -0.82433300<br>H 6.02397600 -1.98734900 0.37666700<br>H 4.33632800 -2.15727100 0.88453000<br>O 2.26838500 -0.50024800 1.90410500<br>H 1.76258600 -1.32345400 2.00588600<br>O 0.50727900 1.44968500 -2.11989200<br>H 1.21334700 1.17565600 -2.72335700	
**Name**	**MB-C14-C5-OH-RAF**
Cartesian Coordinates	Frequency and Energy
C 3.51620000 1.45258200 -0.24935600<br>C 3.68541200 0.00899800 -0.12650800<br>C 2.51480400 -0.82687600 -0.11734800<br>C 2.31192500 1.97805100 -0.48710600<br>C 1.26412300 -0.28101500 -0.33296100<br>S -0.07311300 -1.33971900 -0.58928700<br>N -0.04762500 1.83516800 -0.15311200<br>C 1.07293100 1.16774800 -0.75263500<br>C -1.41490300 -0.22518900 -0.32843400<br>C -2.67264500 -0.77970200 -0.32022500<br>C -3.81446200 -0.00112700 0.00513000<br>C -3.60225400 1.36661000 0.37627900<br>C -2.35525800 1.90507200 0.35730400<br>C -1.18750800 1.17010700 -0.05197100<br>N -5.05040500 -0.53681300 -0.00999000<br>C -5.24397400 -1.93794400 -0.37144500<br>C -6.20712300 0.25925000 0.39342000<br>N 4.87850100 -0.51953300 0.01301500<br>C 6.09093400 0.31232300 -0.03568700<br>C 5.04988100 -1.96277000 0.24232000<br>H 4.37230800 2.09717800 -0.10999900<br>H 2.18064600 3.05281300 -0.55637800<br>H 2.60604000 -1.89081300 0.05245100<br>H -2.78470000 -1.83543100 -0.53357700<br>H -4.43993400 1.98452200 0.66861900<br>H -2.20913200 2.94597600 0.62164200<br>H -6.30792700 -2.15485500 -0.37278800<br>H -4.84466400 -2.13756100 -1.36798200	Zero-point correction= 0.341569 (Hartree/Particle)<br>Thermal correction to Energy= 0.363935<br>Thermal correction to Enthalpy= 0.364879<br>Thermal correction to Gibbs Free Energy= 0.289263<br>Sum of electronic and zero-point Energies= -1333.859193<br>Sum of electronic and thermal Energies= -1333.836826<br>Sum of electronic and thermal Enthalpies= -1333.835882<br>Sum of electronic and thermal Free Energies= -1333.911498

H -4.75049300 -2.59841900 0.34625300<br>H -6.10735000 0.59542300 1.42799500<br>H -6.32224500 1.13167700 -0.25311000<br>H -7.09830000 -0.35563000 0.30982300<br>H 6.17824700 0.89784200 0.88109900<br>H 6.95072100 -0.34276900 -0.12940200<br>H 6.05406500 0.97657800 -0.89657300<br>H 4.69899200 -2.52252900 -0.62522400<br>H 6.10415700 -2.16312100 0.39817400<br>H 4.49251300 -2.26638800 1.12823400<br>O 1.25986400 -0.17189100 1.79594300<br>H 1.88358700 0.53955700 2.02469000<br>O 0.90418000 1.07873900 -2.17469400<br>H 0.76992600 1.97967200 -2.50442200	
**Name**	**MB-C14-C7-OH-RAF**
Cartesian Coordinates	Frequency and Energy
C 3.29458600 1.35127500 -0.51335800<br>C 3.56556400 -0.00471600 -0.16448900<br>C 2.49055400 -0.78416700 0.35396000<br>C 2.04540300 1.87552500 -0.34176000<br>C 1.23840200 -0.19139100 0.66508300<br>S -0.09184100 -1.35703600 0.79941200<br>N -0.22070800 1.78893600 0.34006200<br>C 0.94224400 1.16137000 0.22548600<br>C -1.46983700 -0.31181100 0.62131600<br>C -2.62707400 -0.82701500 0.15676300<br>C -3.80127100 0.00099000 0.02309500<br>C -3.69590200 1.42074900 0.33341400<br>C -2.54369500 1.92834000 0.77406600<br>C -1.29643000 1.13105700 1.03310100<br>N -4.93304700 -0.50831300 -0.39834400<br>C -5.09985100 -1.95420800 -0.60563200<br>C -6.10370600 0.33307400 -0.69187800<br>N 4.77347400 -0.56134600 -0.34861300<br>C 5.84193800 0.21436400 -0.97580400<br>C 5.04331600 -1.94645600 0.04086800<br>H 4.07590800 1.96896700 -0.93229300<br>H 1.85549900 2.90116000 -0.63714300<br>H 2.61829500 -1.84263100 0.53983200<br>H -2.66359900 -1.85654700 -0.17265100<br>H -4.56116900 2.05795100 0.22171000<br>H -2.45916100 2.98405100 1.01286600<br>H -6.16354300 -2.17055300 -0.63355200<br>H -4.64364200 -2.25392000 -1.55095500<br>H -4.65120000 -2.50739500 0.21627400<br>H -6.60366200 0.62008800 0.23463000<br>H -5.80325900 1.22001100 -1.24421400<br>H -6.78640700 -0.24612700 -1.30637700<br>H 6.09337900 1.08032100 -0.35963300<br>H 6.71782000 -0.41791200 -1.07884100<br>H 5.52847500 0.55932100 -1.96297300<br>H 4.49432100 -2.63996600 -0.59999500<br>H 6.10726100 -2.13311700 -0.06644900<br>H 4.75911700 -2.11061600 1.08049700	Zero-point correction= 0.342619 (Hartree/Particle)<br>Thermal correction to Energy= 0.364618<br>Thermal correction to Enthalpy= 0.365563<br>Thermal correction to Gibbs Free Energy= 0.291346<br>Sum of electronic and zero-point Energies= -1333.853747<br>Sum of electronic and thermal Energies= -1333.831748<br>Sum of electronic and thermal Enthalpies= -1333.830804<br>Sum of electronic and thermal Free Energies= -1333.905021

O 1.82852200 -0.04285000 2.49136000<br>H 2.42880100 0.71766000 2.43729200<br>O -1.08428200 1.09955600 2.44713700<br>H -0.86867500 2.00195700 2.72440300	
**Name**	**MB-C14-C8-OH-RAF**
Cartesian Coordinates	Frequency and Energy
C 3.24062100 1.42949300 -0.62458400<br>C 3.53283000 0.06710000 -0.29683300<br>C 2.45199100 -0.76204300 0.11115300<br>C 1.97979900 1.92529700 -0.50690700<br>C 1.14861200 -0.26031900 0.14868800<br>S -0.13701000 -1.42925700 0.28854800<br>N -0.28753200 1.74466000 0.17456800<br>C 0.86846400 1.13825200 -0.04508700<br>C -1.52673700 -0.38165200 0.34056800<br>C -2.72553100 -0.84473800 -0.06067500<br>C -3.90192300 -0.00651400 0.02741600<br>C -3.75213100 1.36537400 0.49907500<br>C -2.55814900 1.82026600 0.87894000<br>C -1.29804300 1.00233800 0.89568300<br>N -5.07458200 -0.46601600 -0.32523000<br>C -5.27266600 -1.87479900 -0.70385300<br>C -6.26683900 0.39755500 -0.35739400<br>N 4.78738700 -0.41713300 -0.35697100<br>C 5.88175000 0.42055900 -0.84246500<br>C 5.06674600 -1.79709500 0.03080800<br>H 4.03504700 2.08513400 -0.95337800<br>H 1.78343600 2.96811200 -0.72687500<br>H 2.59974700 -1.82877300 0.21423600<br>H -2.80822700 -1.83625200 -0.48539000<br>H -4.61884600 2.00758500 0.55571800<br>H -2.44070900 2.83840700 1.23700000<br>H -6.33730900 -2.08459400 -0.67976700<br>H -4.88996800 -2.04824400 -1.71090300<br>H -4.76944400 -2.52905000 0.00465900<br>H -6.62189400 0.58200600 0.65755800<br>H -6.03677700 1.33942400 -0.84951100<br>H -7.03776000 -0.11546000 -0.92316000<br>H 6.04530100 1.27184700 -0.17773500<br>H 6.78702500 -0.17808800 -0.87542600<br>H 5.66959300 0.79057100 -1.84759100<br>H 4.61736500 -2.50127900 -0.67464600<br>H 6.14257900 -1.94630400 0.03914700<br>H 4.67725300 -1.99801300 1.03049000<br>O 2.56574300 -0.52714300 2.36026100<br>H 2.44097500 0.43649400 2.33704900<br>O -0.90779100 0.79795800 2.25302000<br>H -0.69322500 1.66333600 2.63050600	Zero-point correction= 0.341839 (Hartree/Particle)<br>Thermal correction to Energy= 0.364340<br>Thermal correction to Enthalpy= 0.365284<br>Thermal correction to Gibbs Free Energy= 0.289729<br>Sum of electronic and zero-point Energies= -1333.865162<br>Sum of electronic and thermal Energies= -1333.842661<br>Sum of electronic and thermal Enthalpies= -1333.841717<br>Sum of electronic and thermal Free Energies= -1333.917272
**Name**	**MB-C14-C9-OH-RAF**
Cartesian Coordinates	Frequency and Energy
C 3.16118700 1.31841400 -0.79230400<br>C 3.49930800 0.00986700 -0.21657500<br>C 2.37892800 -0.81817600 0.11148700<br>C 1.91487600 1.82281100 -0.73387800	Zero-point correction= 0.342040 (Hartree/Particle)<br>Thermal correction to Energy= 0.363782<br>Thermal correction to Enthalpy= 0.364727<br>Thermal correction to Gibbs Free Energy= 0.291543

C 1.05758200 -0.24297100 0.18927600<br>S -0.19832000 -1.36258600 0.46433300<br>N -0.35081500 1.76495700 0.02572100<br>C 0.79887600 1.11823200 -0.13215200<br>C -1.59568500 -0.31861700 0.41071200<br>C -2.78565400 -0.83653900 0.05830200<br>C -3.96944700 0.00667700 0.04792500<br>C -3.82187000 1.42142800 0.35985000<br>C -2.62919200 1.92041300 0.68950500<br>C -1.36854900 1.11378900 0.81546000<br>N -5.13607500 -0.49828700 -0.25182000<br>C -5.30224700 -1.93035900 -0.56168800<br>C -6.34126000 0.34903600 -0.28653400<br>N 4.69683900 -0.57216000 -0.60952300<br>C 5.89973900 0.24497300 -0.42659600<br>C 4.93179700 -1.94257300 -0.15724000<br>H 3.94381900 1.89370500 -1.26618700<br>H 1.70398200 2.80468800 -1.14205000<br>H 2.51414100 -1.85421700 0.38689500<br>H -2.86623000 -1.87570900 -0.23258900<br>H -4.68628400 2.06818200 0.31916100<br>H -2.51422700 2.97523100 0.91905200<br>H -6.35550000 -2.12268700 -0.73165800<br>H -4.73846500 -2.18257800 -1.45998500<br>H -4.95722200 -2.53457900 0.27669300<br>H -6.51719800 0.78245100 0.69782800<br>H -6.21603700 1.14202600 -1.02300300<br>H -7.18620000 -0.27028600 -0.56492800<br>H 6.21914900 0.23770900 0.62081000<br>H 6.69867600 -0.17105000 -1.03958100<br>H 5.73789800 1.27444300 -0.73337600<br>H 4.19150100 -2.62333800 -0.57572200<br>H 5.91104400 -2.25346200 -0.51765900<br>H 4.92162800 -2.01863200 0.93647200<br>O 3.74099200 0.34398400 1.58761900<br>H 3.06622300 1.00128100 1.80927200<br>O -0.99680300 1.05709500 2.19212900<br>H -0.75384600 1.95383000 2.46584900	Sum of electronic and zero-point Energies= -1333.832244<br>Sum of electronic and thermal Energies= -1333.810501<br>Sum of electronic and thermal Enthalpies= -1333.809557<br>Sum of electronic and thermal Free Energies= -1333.882741
**Name**	**MB-C14-C10-OH-RAF**
Cartesian Coordinates	Frequency and Energy
C -3.26825000 -1.06724500 -0.67240800<br>C -3.47922700 0.27475900 -0.20957000<br>C -2.34756400 1.00618900 0.23174800<br>C -1.98583000 -1.60072500 -0.69893800<br>C -1.09352500 0.44227500 0.25376700<br>S 0.25641400 1.51577800 0.61441300<br>N 0.27271900 -1.60593400 -0.03447600<br>C -0.85795500 -0.92043300 -0.14247900<br>C 1.59669400 0.42173600 0.46164400<br>C 2.81543300 0.89629200 0.13385100<br>C 3.95854000 0.01546100 0.08537900<br>C 3.75752900 -1.41036500 0.31888000<br>C 2.54495600 -1.88047600 0.61055600<br>C 1.31982100 -1.02883100 0.78103900	Zero-point correction= 0.341961 (Hartree/Particle)<br>Thermal correction to Energy= 0.364328<br>Thermal correction to Enthalpy= 0.365272<br>Thermal correction to Gibbs Free Energy= 0.290023<br>Sum of electronic and zero-point Energies= -1333.861983<br>Sum of electronic and thermal Energies= -1333.839617<br>Sum of electronic and thermal Enthalpies= -1333.838673<br>Sum of electronic and thermal Free Energies= -1333.913922

N 5.15108100 0.48139000 -0.18830000<br>C 5.37130300 1.91742000 -0.42701600<br>C 6.31882600 -0.41263400 -0.27305900<br>N -4.71534900 0.80639600 -0.19026700<br>C -5.86238800 0.03485100 -0.66375900<br>C -4.91329900 2.19378400 0.21896700<br>H -4.05795200 -1.59074500 -1.19107000<br>H -1.82874000 -2.59782900 -1.09261000<br>H -2.46248400 2.04151500 0.52817900<br>H 2.93740100 1.94260700 -0.11098600<br>H 4.59678900 -2.08639300 0.24265200<br>H 2.38491100 -2.94202600 0.77224900<br>H 6.43129500 2.08105900 -0.58629400<br>H 4.82022600 2.23738800 -1.31183900<br>H 5.04598000 2.49406800 0.43872600<br>H 6.47525200 -0.91620100 0.68041400<br>H 6.16868400 -1.15116000 -1.06000700<br>H 7.19116900 0.18676500 -0.50856400<br>H -5.89865500 -0.93608300 -0.16815600<br>H -6.77033900 0.58062500 -0.42351800<br>H -5.81543500 -0.11662500 -1.74550300<br>H -4.35856900 2.87327100 -0.43277800<br>H -5.97093500 2.43038100 0.15194500<br>H -4.58321500 2.34251900 1.24936900<br>O -3.61832100 -2.10662400 1.14975400<br>H -3.62858800 -2.99970000 0.77008500<br>O 0.94606400 -1.04319800 2.15745500<br>H 0.72323200 -1.95662000 2.38934000	
**Name**	**MB-C14-C11-OH-RAF**
Cartesian Coordinates	Frequency and Energy
C 3.32361000 1.01261700 -0.75151000<br>C 3.52888300 -0.29127800 -0.19083400<br>C 2.39736400 -0.97995300 0.31154600<br>C 2.08314700 1.64235900 -0.64491500<br>C 1.14708700 -0.39592300 0.29379700<br>S -0.20494400 -1.45088900 0.68875100<br>N -0.21755100 1.63232400 -0.10058500<br>C 0.91608400 0.94911300 -0.14797700<br>C -1.55113500 -0.37698600 0.46205100<br>C -2.76093800 -0.88596700 0.15188700<br>C -3.90803700 -0.02052000 0.01107200<br>C -3.71663800 1.42063200 0.12044200<br>C -2.51538600 1.91936100 0.41495200<br>C -1.29123400 1.09340800 0.69297600<br>N -5.09435900 -0.51765400 -0.23586600<br>C -5.34135900 -1.96726300 -0.23120600<br>C -6.24781400 0.34475700 -0.54007100<br>N 4.75547000 -0.84157400 -0.18699300<br>C 5.90579300 -0.13143000 -0.74875800<br>C 4.95189600 -2.18201300 0.35771800<br>H 4.13651200 1.54435300 -1.22428600<br>H 1.91349600 2.56439800 -1.18419100<br>H 2.49897100 -1.99912300 0.66134000<br>H -2.86651800 -1.94462700 -0.04273800	Zero-point correction= 0.341937 (Hartree/Particle)<br>Thermal correction to Energy= 0.364473<br>Thermal correction to Enthalpy= 0.365417<br>Thermal correction to Gibbs Free Energy= 0.288724<br>Sum of electronic and zero-point Energies= -1333.858732<br>Sum of electronic and thermal Energies= -1333.836196<br>Sum of electronic and thermal Enthalpies= -1333.835252<br>Sum of electronic and thermal Free Energies= -1333.911944

H -4.55935200 2.08260000 -0.01746200<br>H -2.36771300 2.99073600 0.50855000<br>H -6.40961700 -2.12654100 -0.11737100<br>H -5.00617400 -2.40925200 -1.17122500<br>H -4.82649100 -2.43412800 0.60526100<br>H -6.60899100 0.82645400 0.36968500<br>H -5.97328800 1.09697600 -1.27636300<br>H -7.03345800 -0.27924100 -0.95449700<br>H 6.04972900 0.82407600 -0.24223700<br>H 6.78985300 -0.74406500 -0.60379600<br>H 5.76735800 0.04331600 -1.81736500<br>H 4.39681800 -2.91806900 -0.22897700<br>H 6.00978200 -2.42234300 0.32278600<br>H 4.61008500 -2.22329100 1.39368700<br>O 2.79123500 2.63100500 0.95471300<br>H 2.77672200 1.90181300 1.59462300<br>O -0.98915300 1.19561200 2.08897400<br>H -0.72983300 2.11299000 2.25952700	
**Name**	**MB-C14-C12-OH-RAF**
Cartesian Coordinates	Frequency and Energy
C -3.48277400 -1.29098600 0.18847100<br>C -3.71728400 0.08110100 -0.14733200<br>C -2.58813600 0.88095300 -0.46989200<br>C -2.22614500 -1.80445400 0.20129800<br>C -1.32025100 0.36227700 -0.44274400<br>S 0.01949200 1.48736300 -0.68352500<br>N 0.07432100 -1.73591200 -0.44544700<br>C -1.05006100 -1.00748500 -0.05987600<br>C 1.37006900 0.39330300 -0.59555500<br>C 2.56272700 0.84543500 -0.16503800<br>C 3.71467900 -0.02673200 -0.13263600<br>C 3.55369500 -1.41663800 -0.54976700<br>C 2.38102200 -1.85648500 -1.00088300<br>C 1.14566800 -1.01168000 -1.11513800<br>N 4.87569300 0.41621400 0.27279000<br>C 5.10726200 1.83943000 0.56379800<br>C 6.03608800 -0.46818300 0.46492500<br>N -4.96500200 0.59508100 -0.16045300<br>C -6.11194700 -0.24082400 0.18383200<br>C -5.18130500 1.99550300 -0.50916100<br>H -4.31627200 -1.94320700 0.41043200<br>H -2.06374900 -2.85254200 0.42453500<br>H -2.72151100 1.92394800 -0.72884400<br>H 2.64912300 1.85609600 0.20995900<br>H 4.40382500 -2.08263900 -0.52608300<br>H 2.26064900 -2.88116100 -1.33521200<br>H 6.17831600 2.01567300 0.53173400<br>H 4.62607700 2.46261700 -0.18562600<br>H 4.72741700 2.08318700 1.55751100<br>H 5.72691000 -1.41694500 0.89363600<br>H 6.54265500 -0.63049800 -0.48784900<br>H 6.71461700 0.02317700 1.15672000<br>H -6.01449000 -0.63662700 1.19721500<br>H -7.01243000 0.36398100 0.13117300	Zero-point correction= 0.342327 (Hartree/Particle)<br>Thermal correction to Energy= 0.364485<br>Thermal correction to Enthalpy= 0.365429<br>Thermal correction to Gibbs Free Energy= 0.290791<br>Sum of electronic and zero-point Energies= -1333.847633<br>Sum of electronic and thermal Energies= -1333.825475<br>Sum of electronic and thermal Enthalpies= -1333.824531<br>Sum of electronic and thermal Free Energies= -1333.899169

H -6.21001700 -1.07557900 -0.51402500<br>H -4.83248100 2.20266000 -1.52361900<br>H -6.24443400 2.20997300 -0.45531200<br>H -4.65559200 2.65442800 0.18628400<br>O -0.22356600 -0.74333600 1.60276300<br>H -0.23362800 -1.64406900 1.96353100<br>O 0.85810700 -0.97015600 -2.50660500<br>H 0.06562000 -0.43643500 -2.66494500	
**Name**	**MB-C14-N13-OH-RAF**
Cartesian Coordinates	Frequency and Energy
C 3.50675800 -1.18857100 0.40405700<br>C 3.61742500 0.18993200 -0.06245600<br>C 2.43600900 1.02212400 -0.07124300<br>C 2.33622600 -1.66446900 0.82583800<br>C 1.26324700 0.54480500 0.38713300<br>S -0.12971300 1.58142200 0.44485300<br>N 0.04101200 -1.58232500 0.21906600<br>C 1.06865500 -0.85937500 0.91060900<br>C -1.43433000 0.39853500 0.27792100<br>C -2.71468000 0.88844300 0.20628300<br>C -3.79430200 0.04441700 -0.17662200<br>C -3.48692100 -1.29577300 -0.57448300<br>C -2.21241200 -1.76515200 -0.49859900<br>C -1.13390200 -0.98110100 0.02377800<br>N -5.05825200 0.50461800 -0.20620200<br>C -5.37414400 1.85448400 0.25569900<br>C -6.13595800 -0.31853500 -0.75408000<br>N 4.76516800 0.65281500 -0.48566300<br>C 5.94689300 -0.21647200 -0.61986000<br>C 4.93397400 2.06074200 -0.87704200<br>H 4.38436100 -1.81843700 0.41456900<br>H 2.24538600 -2.68945600 1.17258500<br>H 2.48697200 2.01847800 -0.48828600<br>H -2.88972500 1.94453400 0.37159200<br>H -4.27405100 -1.95073400 -0.92103100<br>H -1.98838900 -2.78859700 -0.77528500<br>H -6.45281000 1.95036000 0.33899700<br>H -4.92889400 2.03427600 1.23443100<br>H -5.00270400 2.60225200 -0.45040300<br>H -5.88952400 -0.64885500 -1.76431800<br>H -6.30968400 -1.19394700 -0.12429100<br>H -7.04188800 0.27785900 -0.79052500<br>H 5.69116000 -1.11594400 -1.17631400<br>H 6.70609800 0.33172800 -1.16802600<br>H 6.33185600 -0.48303200 0.36508000<br>H 4.44302300 2.71232600 -0.15825200<br>H 5.99619100 2.28428300 -0.88458800<br>H 4.52041200 2.22430200 -1.87368200<br>O 0.67063300 -1.36759200 -1.78900000<br>H 1.07886000 -2.24534100 -1.74573300<br>O 0.73786000 -0.69341500 2.29258200<br>H 0.53009200 -1.57069600 2.64517900	Zero-point correction= 0.340821 (Hartree/Particle)<br>Thermal correction to Energy= 0.362048<br>Thermal correction to Enthalpy= 0.362992<br>Thermal correction to Gibbs Free Energy= 0.289983<br>Sum of electronic and zero-point Energies= -1333.854295<br>Sum of electronic and thermal Energies= -1333.833069<br>Sum of electronic and thermal Enthalpies= -1333.832125<br>Sum of electronic and thermal Free Energies= -1333.905133
**Name**	**MB-C14-C16-OH-FHT**
Cartesian Coordinates	Frequency and Energy

C -3.54536400 -1.35336500 0.08673400<br>C -3.69552200 0.06511200 -0.21562300<br>C -2.53491000 0.92545100 -0.06291200<br>C -2.37700400 -1.84232200 0.50139400<br>C -1.36385600 0.42825000 0.35847900<br>S 0.00947000 1.50139600 0.57922100<br>N -0.06459900 -1.61966300 -0.09024100<br>C -1.14202500 -1.01491800 0.71407800<br>C 1.31438200 0.40776200 0.19307900<br>C 2.58584800 0.85746200 0.25065100<br>C 3.67987800 0.05526800 -0.25499800<br>C 3.38029400 -1.21496200 -0.90976600<br>C 2.12876900 -1.68221600 -0.91850300<br>C 1.02356900 -0.97079900 -0.26450900<br>N 4.91173300 0.47677100 -0.15312300<br>C 5.26652500 1.66914000 0.63157700<br>C 6.03836800 -0.20894800 -0.80706000<br>N -4.83865600 0.54165500 -0.64144700<br>C -6.00043200 -0.27260800 -0.84181200<br>C -5.02891700 1.99575600 -0.80660900<br>H -4.40050100 -2.00847700 -0.00212100<br>H -2.26447600 -2.89557100 0.74012600<br>H -2.60511300 1.97215200 -0.32855300<br>H 2.78844100 1.86477700 0.59023200<br>H 4.18097600 -1.80053800 -1.33656300<br>H 1.89247400 -2.64862000 -1.34663100<br>H 6.31309400 1.58226800 0.90976800<br>H 5.12495500 2.56589400 0.02544600<br>H 4.66314600 1.72142500 1.53345100<br>H 6.36103700 -1.05320900 -0.19545000<br>H 5.75606400 -0.54659200 -1.80026200<br>H 6.85110200 0.50629200 -0.89586100<br>H -6.42556800 -0.59287200 0.35904000<br>H -6.73936800 0.28754100 -1.40631600<br>H -5.74962300 -1.20106400 -1.35065200<br>H -4.52568800 2.32688900 -1.71564600<br>H -6.09340000 2.18688000 -0.89081200<br>H -4.63665700 2.52927800 0.05572100<br>O -0.76580200 -1.05126800 2.07753500<br>H -0.68733800 -1.97685600 2.35033700<br>O -6.71873100 -0.92674800 1.59139300<br>H -5.97183100 -1.47306800 1.85984500	Zero-point correction= 0.338959 (Hartree/Particle)<br>Thermal correction to Energy= 0.361441<br>Thermal correction to Enthalpy= 0.362386<br>Thermal correction to Gibbs Free Energy= 0.286192<br>Sum of electronic and zero-point Energies= -1333.880311<br>Sum of electronic and thermal Energies= -1333.857829<br>Sum of electronic and thermal Enthalpies= -1333.856885<br>Sum of electronic and thermal Free Energies= -1333.933078
**Name**	**MB-C14-C17-OH-FHT**
Cartesian Coordinates	Frequency and Energy
C -3.25490400 -1.76525500 -0.35463500<br>C -3.48651000 -0.32857000 -0.31722200<br>C -2.36822500 0.56355500 -0.45024400<br>C -2.02723100 -2.24334900 -0.56439300<br>C -1.12546000 0.07756400 -0.67643200<br>S 0.19406600 1.17514500 -0.89188500<br>N 0.22665900 -1.85584800 0.06852700<br>C -0.81390800 -1.39472600 -0.81646500<br>C 1.56138100 0.16359300 -0.42967300<br>C 2.80485900 0.74480100 -0.47767400	Zero-point correction= 0.337882 (Hartree/Particle)<br>Thermal correction to Energy= 0.360011<br>Thermal correction to Enthalpy= 0.360955<br>Thermal correction to Gibbs Free Energy= 0.285696<br>Sum of electronic and zero-point Energies= -1333.855830<br>Sum of electronic and thermal Energies= -1333.833701<br>Sum of electronic and thermal Enthalpies= -1333.832757<br>Sum of electronic and thermal Free Energies= -1333.908016

C	3.93971800	0.08219300	0.06370600	
C	3.72836400	-1.17419600	0.71951100	
C	2.49410900	-1.74215800	0.74953000	
C	1.34727800	-1.15041400	0.11500700	
N	5.16672400	0.63155900	-0.01263500	
C	5.36526100	1.90837600	-0.69207200	
C	6.30579500	-0.00578300	0.64410000	
N	-4.70795300	0.13675300	-0.13369700	
C	-5.83096400	-0.76084700	0.19172700	
C	-4.99461200	1.55179800	-0.17767700	
H	-4.08528400	-2.44381300	-0.22475000	
H	-1.84634800	-3.31306700	-0.59891600	
H	-2.50877100	1.62882000	-0.32203000	
H	2.90905200	1.73971300	-0.89224600	
H	4.55847500	-1.68817100	1.18398700	
H	2.34996400	-2.70591600	1.22394100	
H	6.42982000	2.11770100	-0.73778700	
H	4.97453500	1.86512900	-1.71017900	
H	4.86752800	2.71998900	-0.15462300	
H	6.12666400	-0.10888400	1.71646600	
H	6.49584300	-0.99390700	0.21957100	
H	7.18489500	0.61386600	0.49403400	
H	-5.55963500	-1.41703400	1.01646800	
H	-6.67546700	-0.14855600	0.49144500	
H	-6.10425800	-1.35137700	-0.68314500	
H	-4.43539300	2.06265100	-0.95660300	
H	-6.06272000	1.71300300	-0.29045800	
H	-4.70492800	2.03814700	0.83551200	
O	-4.09868000	2.53772400	2.06587000	
H	-3.19819100	2.20176400	1.90520400	
O	-0.43293700	-1.55705800	-2.18559400	
H	-0.15759700	-2.47848700	-2.30046000	
**Name**				**MB-C14-C19-OH-FHT**
Cartesian Coordinates				Frequency and Energy
C	-3.43866000	-1.28842400	0.17263000	Zero-point correction= 0.339263 (Hartree/Particle)
C	-3.62293200	0.12546000	-0.14054700	Thermal correction to Energy= 0.361423
C	-2.46388700	0.99597000	-0.14827700	Thermal correction to Enthalpy= 0.362367
C	-2.23323600	-1.75431700	0.49358500	Thermal correction to Gibbs Free Energy= 0.287695
C	-1.25548800	0.52525300	0.20243200	Sum of electronic and zero-point Energies= -1333.883440
S	0.10558900	1.61052300	0.29549200	Sum of electronic and thermal Energies= -1333.861280
N	0.04394500	-1.54165700	-0.19600200	Sum of electronic and thermal Enthalpies= -1333.860336
C	-1.00020500	-0.90476400	0.60848500	Sum of electronic and thermal Free Energies= -1333.935008
C	1.42680700	0.48738300	0.00433500	
C	2.69038300	0.95649600	0.04564600	
C	3.80438100	0.11848500	-0.34875200	
C	3.51545500	-1.17685300	-0.94501300	
C	2.25854500	-1.63773800	-0.98743900	
C	1.14472300	-0.90686200	-0.38194900	
N	5.04116400	0.53563400	-0.19562200	
C	5.37501900	1.78288400	0.41420900	
C	6.16706000	-0.37363500	-0.48904400	
N	-4.80811800	0.59446600	-0.42205900	
C	-5.99291100	-0.28299400	-0.43400500	
C	-5.01433200	2.02081700	-0.73251300	

H -4.28701600 -1.95654800 0.14008200	
H -2.08414300 -2.80503600 0.72240500	
H -2.57578200 2.03032200 -0.44431800	
H 2.87468400 1.97483100 0.36195800	
H 4.31930000 -1.76927100 -1.35880900	
H 2.03027200 -2.60815300 -1.41055300	
H 6.42921500 1.99252000 0.26973800	
H 4.76122300 2.59738800 0.03500600	
H 5.06219900 1.64713100 1.63944000	
H 6.00558800 -1.33535900 -0.00665100	
H 6.25547900 -0.50522300 -1.56774300	
H 7.07367900 0.07853700 -0.10276300	
H -6.14062100 -0.72081100 0.55265800	
H -6.85798100 0.31606800 -0.69479700	
H -5.86494600 -1.07172100 -1.17436400	
H -4.46644600 2.28625700 -1.63675600	
H -6.07367700 2.18736600 -0.89195200	
H -4.67470100 2.63494400 0.10102200	
O -0.59653400 -0.85359100 1.96474300	
H -0.45503400 -1.76077100 2.27314500	
O 4.57578300 1.42394800 2.89771000	
H 3.66538400 1.14466100 2.75019700	
**Name**	**MB-C14-C20-OH-FHT**
Cartesian Coordinates	Frequency and Energy
C -3.16008000 -1.25153700 -0.05232900	Zero-point correction= 0.337575 (Hartree/Particle)
C -3.36022900 0.13380200 -0.33812600	Thermal correction to Energy= 0.359981
C -2.21682600 0.94165800 -0.54243300	Thermal correction to Enthalpy= 0.360925
C -1.90818600 -1.78705200 -0.06168000	Thermal correction to Gibbs Free Energy= 0.284727
C -0.95369900 0.39284700 -0.53940000	Sum of electronic and zero-point Energies= -1333.858162
S 0.40025300 1.51639900 -0.57179500	Sum of electronic and thermal Energies= -1333.835756
N 0.41159800 -1.66908400 -0.51182700	Sum of electronic and thermal Enthalpies= -1333.834812
C -0.73746900 -1.01899800 -0.37741300	Sum of electronic and thermal Free Energies= -1333.911010
C 1.74196400 0.41360600 -0.50436300	
C 2.91787900 0.82061200 0.01365400	
C 4.06261800 -0.06102400 0.01768500	
C 3.90760700 -1.41710700 -0.49563700	
C 2.73617300 -1.82149100 -0.98642400	
C 1.51738700 -0.95202400 -1.10756500	
N 5.21306200 0.34090100 0.49540600	
C 5.39610100 1.71725400 0.98391100	
C 6.36693300 -0.57135300 0.58138300	
N -4.61096000 0.67343200 -0.38610600	
C -5.75197300 -0.11181300 -0.00161600	
C -4.77664700 2.12177700 -0.48108400	
H -4.00179700 -1.89278300 0.16966900	
H -1.76688000 -2.84411000 0.13093300	
H -2.32483500 2.01152000 -0.66631700	
H 2.99939300 1.80426300 0.45625200	
H 4.74925000 -2.09410700 -0.47434800	
H 2.61089400 -2.83162400 -1.36392000	
H 6.44973700 1.87149300 1.19010400	
H 5.07426700 2.42794600 0.22453700	
H 4.82165900 1.86845400 1.89881800	
H 6.10313000 -1.45495300 1.16109400	

H	6.68665100	-0.86614600	-0.41825300	
H	7.17732900	-0.04983200	1.07849500	
H	-5.74236300	-0.31709200	1.12856200	
H	-6.66808200	0.43808900	-0.20056000	
H	-5.77794300	-1.08099100	-0.49679300	
H	-4.30428300	2.50149300	-1.38793100	
H	-5.83854600	2.34613600	-0.52729600	
H	-4.34169400	2.62576000	0.38661400	
O	-5.43137800	-0.61035400	2.60325400	
H	-4.54518300	-0.97713400	2.43366500	
O	1.27320100	-0.70912000	-2.49260700	
H	1.08695400	-1.56252400	-2.91022400	
**Name**				**MB-C16-C1-OH-RAF**
Cartesian Coordinates				Frequency and Energy
C	3.53139400	1.11916600	-0.34404700	Zero-point correction= 0.314629 (Hartree/Particle)
C	3.59934800	-0.26883800	-0.29023300	Thermal correction to Energy= 0.335139
C	2.45304700	-1.03363600	-0.15148400	Thermal correction to Enthalpy= 0.336083
C	2.28768100	1.74994200	-0.19793500	Thermal correction to Gibbs Free Energy= 0.263490
C	1.19793100	-0.40778500	-0.10045200	Sum of electronic and zero-point Energies= -1257.468733
S	-0.19118300	-1.47068500	0.02841400	Sum of electronic and thermal Energies= -1257.448223
N	-0.08735300	1.70011400	-0.14996900	Sum of electronic and thermal Enthalpies= -1257.447279
C	1.07127100	0.98626200	-0.16215900	Sum of electronic and thermal Free Energies= -1257.519872
C	-1.52241100	-0.32960400	-0.05281000	
C	-2.79724700	-0.85971500	-0.02819200	
C	-3.93451500	-0.01921100	-0.06503400	
C	-3.71269300	1.39080700	-0.12153100	
C	-2.44578300	1.89950500	-0.14602300	
C	-1.28276000	1.07922900	-0.11622900	
N	-5.18738700	-0.52641800	-0.04754900	
C	-5.39085400	-1.96766200	0.01821500	
C	-6.34519500	0.36083100	-0.07468900	
N	4.88332900	-0.92114000	-0.37885200	
C	5.75591000	-0.54719300	-1.23310400	
C	5.15641800	-2.00944200	0.58001400	
H	4.43004000	1.71731500	-0.43319500	
H	2.20097700	2.80412600	-0.42372800	
H	2.51463900	-2.11557700	-0.11583000	
H	-2.91754800	-1.93442900	0.02132600	
H	-4.55346700	2.07033000	-0.14700000	
H	-2.29203100	2.97131600	-0.19148900	
H	-6.45756900	-2.17291400	0.01022300	
H	-4.93300800	-2.46708700	-0.83972700	
H	-4.96140000	-2.38401900	0.93370400	
H	-6.35223600	1.02756200	0.79136800	
H	-6.35621200	0.96678100	-0.98407600	
H	-7.24656300	-0.24479200	-0.05202500	
H	6.72891400	-1.02263100	-1.23330500	
H	5.51144100	0.23144400	-1.94612400	
H	4.52391100	-2.86095500	0.33612500	
H	6.20336100	-2.28784100	0.49969400	
H	4.92386100	-1.64559600	1.57887000	
O	2.50111600	2.21492700	1.75326500	
H	2.59748200	1.31092000	2.09276200	
**Name**				**MB-C16-C2-OH-RAF**

Cartesian Coordinates	Frequency and Energy
C -3.44820300 -1.20679000 -0.47026700	Zero-point correction= 0.314236 (Hartree/Particle)
C -3.54131400 0.18949000 -0.25627900	Thermal correction to Energy= 0.334741
C -2.40506500 0.95083800 -0.08015800	Thermal correction to Enthalpy= 0.335685
C -2.18509200 -1.78497300 -0.56687600	Thermal correction to Gibbs Free Energy= 0.263192
C -1.14492200 0.34507200 -0.12485800	Sum of electronic and zero-point Energies= -1257.476510
S 0.23135900 1.38620900 0.14026400	Sum of electronic and thermal Energies= -1257.456005
N 0.17049200 -1.73177600 -0.45135700	Sum of electronic and thermal Enthalpies= -1257.455061
C -0.99861600 -1.04901000 -0.36900400	Sum of electronic and thermal Free Energies= -1257.527553
C 1.57904600 0.26940000 -0.04432800	
C 2.84398900 0.80409200 0.08517700	
C 3.99470300 -0.01328700 -0.03792300	
C 3.79320500 -1.40255600 -0.28898400	
C 2.53128900 -1.91295800 -0.41552700	
C 1.36044400 -1.11436300 -0.30438500	
N 5.23909300 0.50558700 0.07709100	
C 5.41420000 1.90598100 0.44120400	
C 6.40325800 -0.37322900 0.05963800	
N -4.83397800 0.80407200 -0.18059700	
C -5.87635100 0.14222600 0.16324700	
C -4.93463600 2.24528100 -0.49389800	
H -4.31106500 -1.77316200 -0.79348900	
H -2.09859800 -2.84160500 -0.78743900	
H -2.47204200 2.01507200 0.11033400	
H 2.94920800 1.86285800 0.28399000	
H 4.64057200 -2.06756000 -0.38002000	
H 2.39133800 -2.97060300 -0.60753300	
H 6.47714500 2.12736800 0.47969100	
H 4.95272700 2.56219000 -0.30025700	
H 4.97454500 2.12031900 1.42024700	
H 6.38210200 -1.08011500 0.89454600	
H 6.45341600 -0.93623500 -0.87427600	
H 7.29970800 0.23539300 0.13941300	
H -6.83415700 0.64624600 0.17224100	
H -5.79022000 -0.89709500 0.45109800	
H -4.39095400 2.43782600 -1.41582300	
H -5.98457000 2.49791500 -0.60635300	
H -4.50250100 2.81346100 0.32786900	
O -3.87212200 -1.84608500 1.53545300	
H -2.99926700 -1.61562000 1.89590700	
**Name**	**MB-C16-C3-OH-RAF**
Cartesian Coordinates	Frequency and Energy
C 3.39858200 1.42599400 -0.20307400	Zero-point correction= 0.314822 (Hartree/Particle)
C 3.54392500 0.03238100 0.04855700	Thermal correction to Energy= 0.334923
C 2.40781200 -0.80775300 -0.09702300	Thermal correction to Enthalpy= 0.335867
C 2.14877700 1.95699000 -0.35172900	Thermal correction to Gibbs Free Energy= 0.264872
C 1.15608200 -0.25327900 -0.24295100	Sum of electronic and zero-point Energies= -1257.464104
S -0.20510600 -1.37089600 -0.36359400	Sum of electronic and thermal Energies= -1257.444003
N -0.21225400 1.80688800 -0.49237200	Sum of electronic and thermal Enthalpies= -1257.443059
C 0.97801000 1.15266500 -0.35848500	Sum of electronic and thermal Free Energies= -1257.514053
C -1.56649100 -0.26492700 -0.22499900	
C -2.81175600 -0.82007500 -0.05289100	
C -3.97842500 -0.01045100 -0.04097300	
C -3.81282700 1.39790800 -0.22422900	

C -2.57006800 1.93613800 -0.38095800		
C -1.37915400 1.15035900 -0.37443400		
N -5.20089800 -0.54857400 0.13563500		
C -5.35303600 -1.98837400 0.32029900		
C -6.39084700 0.29904800 0.15022400		
N 4.85954200 -0.54617800 -0.16399500		
C 5.89051600 -0.04038500 0.39337400		
C 4.96887800 -1.69963400 -1.07834900		
H 4.28126100 2.05364300 -0.23787200		
H 2.02175900 3.02286200 -0.49518600		
H 2.52375900 -1.88147400 -0.00552800		
H -2.89765300 -1.89256200 0.06452900		
H -4.67516600 2.05001200 -0.23344500		
H -2.45268300 3.00586100 -0.50698900		
H -6.40784900 -2.21626700 0.43965100		
H -4.97040000 -2.53175400 -0.54678700		
H -4.81572100 -2.32235800 1.21123000		
H -6.32730200 1.04218500 0.94797500		
H -6.51308000 0.81379200 -0.80516000		
H -7.26041100 -0.32716200 0.32433900		
H 6.86408000 -0.45891900 0.16832300		
H 5.77903900 0.79106800 1.07754300		
H 4.43361200 -1.46386500 -1.99617300		
H 6.02212200 -1.87425100 -1.27834400		
H 4.52924400 -2.57312600 -0.60015900		
O 3.67093900 0.00210800 1.99987400		
H 2.78743000 0.33138500 2.22484500		
**Name**	**MB-C16-C4-OH-RAF**	
Cartesian Coordinates	Frequency and Energy	
C 3.46964400 1.48286000 -0.04096300	Zero-point correction=	0.315004 (Hartree/Particle)
C 3.60450600 0.12330300 -0.27364200	Thermal correction to Energy=	0.335256
C 2.48068900 -0.72513000 -0.30837800	Thermal correction to Enthalpy=	0.336200
C 2.20193400 2.02050500 0.09485600	Thermal correction to Gibbs Free Energy=	0.264709
C 1.19859300 -0.14658300 -0.26371100	Sum of electronic and zero-point Energies=	-1257.475228
S -0.14809000 -1.23266000 -0.47270800	Sum of electronic and thermal Energies=	-1257.454976
N -0.15847700 1.88742200 0.11315800	Sum of electronic and thermal Enthalpies=	-1257.454032
C 1.02622700 1.23931000 -0.01889800	Sum of electronic and thermal Free Energies=	-1257.525523
C -1.52055700 -0.16052900 -0.21900300		
C -2.77243300 -0.73776100 -0.28058800		
C -3.94127100 0.04045100 -0.09606300		
C -3.77266300 1.43411700 0.15483200		
C -2.52344600 1.98718200 0.21082400		
C -1.33407500 1.22962400 0.03048600		
N -5.17218700 -0.51834700 -0.15559400		
C -5.31163000 -1.95725900 -0.33895800		
C -6.35626000 0.28307700 0.13262200		
N 4.91582900 -0.43404900 -0.47608400		
C 5.76668600 0.16758900 -1.21747100		
C 5.26227500 -1.68539200 0.22827900		
H 4.34560300 2.11355200 0.05180800		
H 2.08196200 3.07793200 0.29648600		
H 2.58879900 -1.75419700 -0.62724100		
H -2.85287000 -1.80049700 -0.46930400		
H -4.63577400 2.06767500 0.30488800		

H -2.40921600 3.04778700 0.40362800	
H -6.36867900 -2.20607100 -0.37100100	
H -4.85400800 -2.27737300 -1.27804100	
H -4.84531100 -2.50952600 0.48262500	
H -6.32275600 0.69120900 1.14682300	
H -6.45240500 1.11005000 -0.57452600	
H -7.23522200 -0.34904800 0.04113800	
H 6.76489000 -0.24144300 -1.31383800	
H 5.47657800 1.06989600 -1.74263000	
H 4.56714900 -2.46729100 -0.06917600	
H 6.27583800 -1.96094200 -0.04771500	
H 5.17982600 -1.50377600 1.29702000	
O 2.57746200 -1.39739700 1.72612900	
H 2.14490900 -0.60694400 2.09071800	
**Name**	**MB-C16-C5-OH-RAF**
Cartesian Coordinates	Frequency and Energy
C 3.55175200 1.41337400 -0.10052900	Zero-point correction= 0.315074 (Hartree/Particle)
C 3.66999100 0.02260400 -0.20978900	Thermal correction to Energy= 0.335115
C 2.54977100 -0.77986800 -0.29198400	Thermal correction to Enthalpy= 0.336059
C 2.30025700 2.00216900 -0.09603100	Thermal correction to Gibbs Free Energy= 0.265601
C 1.26663200 -0.19304100 -0.22302900	Sum of electronic and zero-point Energies= -1257.465610
S -0.05747900 -1.16508700 -0.88378400	Sum of electronic and thermal Energies= -1257.445569
N -0.06679900 1.90457300 -0.15110200	Sum of electronic and thermal Enthalpies= -1257.444625
C 1.12245300 1.24425000 -0.18964100	Sum of electronic and thermal Free Energies= -1257.515083
C -1.42023700 -0.16862500 -0.37572900	
C -2.65874600 -0.75449800 -0.31838500	
C -3.82181900 0.01770100 -0.03732900	
C -3.65325400 1.41861100 0.18982200	
C -2.41380700 1.98389100 0.13989800	
C -1.22985200 1.23363200 -0.13397900	
N -5.04012000 -0.55312900 0.00773900	
C -5.19652300 -1.98600800 -0.22888000	
C -6.22633900 0.25353300 0.28840500	
N 4.97771800 -0.57972900 -0.23325200	
C 5.96139800 -0.02483400 -0.83242500	
C 5.14327700 -1.86744600 0.46947300	
H 4.43731900 2.02893100 0.00560700	
H 2.20857600 3.07735500 -0.00739900	
H 2.63000500 -1.85562800 -0.39424500	
H -2.74832300 -1.81672200 -0.50588500	
H -4.51083100 2.04018500 0.40677700	
H -2.29345000 3.04565800 0.31970500	
H -6.24961200 -2.23963400 -0.15791300	
H -4.83534500 -2.25379500 -1.22425200	
H -4.64382500 -2.56238300 0.51633200	
H -6.14743300 0.72736100 1.26896100	
H -6.35517700 1.02764100 -0.47093800	
H -7.09769200 -0.39404600 0.28143900	
H 6.93898600 -0.48774300 -0.77721900	
H 5.80621800 0.89031700 -1.39115100	
H 4.64680100 -2.64632100 -0.10639900	
H 6.20480100 -2.08274800 0.54628800	
H 4.68923900 -1.78052600 1.45417300	
O 0.88394500 -0.45333600 1.73884800	

H 1.59783900 0.07793100 2.12530800	
**Name**	**MB-C16-C7-OH-RAF**
Cartesian Coordinates	Frequency and Energy
C 3.52566000 1.45046100 -0.27930400<br>C 3.70264100 0.05096800 -0.11035900<br>C 2.53286200 -0.77223200 -0.08815800<br>C 2.26509000 1.98275300 -0.39171400<br>C 1.24102300 -0.20131100 -0.10440700<br>S -0.06814700 -1.28779700 -0.57611200<br>N -0.10389600 1.85227500 -0.39906900<br>C 1.07147400 1.21552900 -0.33360200<br>C -1.44342000 -0.22975200 -0.25957700<br>C -2.69013300 -0.82045800 -0.10697500<br>C -3.80866300 -0.01471100 0.06843300<br>C -3.69755100 1.37617300 0.07336200<br>C -2.45378000 1.95082200 -0.07691300<br>C -1.28268500 1.17812800 -0.24134100<br>N -5.10230700 -0.62664500 0.23339600<br>C -5.39975600 -1.82020000 -0.58380100<br>C -5.96746600 -0.15190900 1.04629200<br>N 4.91732500 -0.51380800 -0.00628800<br>C 6.11429500 0.31343800 -0.13554500<br>C 5.07232900 -1.95019500 0.22692200<br>H 4.38338800 2.10633000 -0.31996500<br>H 2.15110600 3.05274300 -0.52571000<br>H 2.61951800 -1.84965900 -0.04585300<br>H -2.78016400 -1.90012400 -0.11070500<br>H -4.57981500 1.99848300 0.16341800<br>H -2.35239000 3.02924400 -0.08852000<br>H -6.46408000 -2.02675100 -0.51972900<br>H -5.10441000 -1.61345000 -1.61009500<br>H -4.83438600 -2.66428800 -0.19291400<br>H -5.70921000 0.69246100 1.67373200<br>H -6.94797700 -0.60860500 1.09986000<br>H 6.16791500 1.03746500 0.68119600<br>H 6.98943500 -0.32806100 -0.10171900<br>H 6.10132300 0.85134200 -1.08528700<br>H 4.76229100 -2.52117700 -0.65118200<br>H 6.11894700 -2.15523300 0.42973500<br>H 4.48022200 -2.26153100 1.08800200<br>O 1.20655400 -0.39353800 1.84482300<br>H 1.79546900 0.32773800 2.11669500	Zero-point correction= 0.315707 (Hartree/Particle)<br>Thermal correction to Energy= 0.335669<br>Thermal correction to Enthalpy= 0.336613<br>Thermal correction to Gibbs Free Energy= 0.265861<br>Sum of electronic and zero-point Energies= -1257.467911<br>Sum of electronic and thermal Energies= -1257.447949<br>Sum of electronic and thermal Enthalpies= -1257.447005<br>Sum of electronic and thermal Free Energies= -1257.517757
**Name**	**MB-C16-C8-OH-RAF**
Cartesian Coordinates	Frequency and Energy
C 3.58813000 1.46287800 -0.04330600<br>C 3.76944900 0.05140600 -0.00182800<br>C 2.59944100 -0.76990100 0.11192000<br>C 2.33661600 2.01146800 -0.03205600<br>C 1.31969200 -0.17857300 0.00530200<br>S -0.01611400 -1.27488300 -0.02878600<br>N -0.03310300 1.88382600 -0.00986200<br>C 1.14562100 1.22636500 -0.00765600<br>C -1.38019200 -0.18382900 -0.03945200<br>C -2.64598300 -0.75802200 -0.06870700	Zero-point correction= 0.316208 (Hartree/Particle)<br>Thermal correction to Energy= 0.336098<br>Thermal correction to Enthalpy= 0.337042<br>Thermal correction to Gibbs Free Energy= 0.266930<br>Sum of electronic and zero-point Energies= -1257.484896<br>Sum of electronic and thermal Energies= -1257.465006<br>Sum of electronic and thermal Enthalpies= -1257.464062<br>Sum of electronic and thermal Free Energies= -1257.534174

C -3.77752200 0.04878400 -0.05475000<br>C -3.64016800 1.45101400 0.02001100<br>C -2.39312200 2.01048800 0.03360200<br>C -1.20520100 1.22649600 -0.01045200<br>N -5.06900400 -0.55782000 -0.12361200<br>C -5.15641800 -2.01793700 0.09629000<br>C -6.14423100 0.09466200 -0.39857000<br>N 4.98999400 -0.51077600 -0.01656900<br>C 6.18187300 0.31862100 -0.16664200<br>C 5.15042400 -1.95215300 0.16443400<br>H 4.44855500 2.11652500 -0.07720500<br>H 2.21074300 3.08732300 -0.05111100<br>H 2.68406300 -1.83275200 -0.06787600<br>H -2.72321700 -1.83636100 -0.11498000<br>H -4.50068200 2.10068300 0.10461700<br>H -2.27332300 3.08469700 0.09956300<br>H -6.20280500 -2.29583700 0.17002000<br>H -4.70303200 -2.52572500 -0.75311600<br>H -4.63312900 -2.27055100 1.01557400<br>H -6.10246200 1.14510800 -0.64498100<br>H -7.08941500 -0.42913000 -0.39060900<br>H 6.34382000 0.93919300 0.71893400<br>H 7.04260500 -0.33022600 -0.30262600<br>H 6.09438900 0.96267700 -1.04313400<br>H 4.83502300 -2.49788000 -0.72944600<br>H 6.20002000 -2.16412700 0.34858400<br>H 4.56777200 -2.28903900 1.02211500<br>O 2.64161700 -1.13467200 2.09671600<br>H 2.60008300 -0.21426900 2.39426900	
**Name**	**MB-C16-C9-OH-RAF**
Cartesian Coordinates	Frequency and Energy
C 3.37057200 1.47384000 -0.35214400<br>C 3.58886100 0.04821800 -0.13719500<br>C 2.39618000 -0.76105900 -0.10601100<br>C 2.12429400 2.00293200 -0.34588300<br>C 1.11636000 -0.16601400 -0.08202600<br>S -0.20459800 -1.27525300 0.06575600<br>N -0.26408800 1.87926000 -0.18372300<br>C 0.93679600 1.21593300 -0.18992400<br>C -1.57399600 -0.20849700 0.01664300<br>C -2.83626100 -0.80339900 0.09382300<br>C -3.96075300 -0.00583300 0.08710300<br>C -3.85502600 1.39666900 -0.02393500<br>C -2.62252900 1.97711300 -0.10571200<br>C -1.41652400 1.21076000 -0.08646600<br>N -5.26134600 -0.61502600 0.16948600<br>C -5.44916400 -1.92173300 -0.49520600<br>C -6.22803100 -0.05321300 0.79131600<br>N 4.78743700 -0.51331600 -0.54803300<br>C 6.00274300 0.21886400 -0.18192200<br>C 4.94168200 -1.95000100 -0.31678800<br>H 4.22037000 2.12455200 -0.49443200<br>H 1.98814100 3.06962300 -0.48324900<br>H 2.47842700 -1.83604200 -0.02738800	Zero-point correction= 0.315553 (Hartree/Particle)<br>Thermal correction to Energy= 0.335159<br>Thermal correction to Enthalpy= 0.336103<br>Thermal correction to Gibbs Free Energy= 0.266880<br>Sum of electronic and zero-point Energies= -1257.461636<br>Sum of electronic and thermal Energies= -1257.442030<br>Sum of electronic and thermal Enthalpies= -1257.441086<br>Sum of electronic and thermal Free Energies= -1257.510308

H	-2.91760700	-1.88062500	0.17662800	
H	-4.74669500	2.00944000	-0.07841000	
H	-2.52702500	3.05064800	-0.21004400	
H	-6.51427900	-2.12584600	-0.55091200	
H	-5.01100900	-1.86777800	-1.48893800	
H	-4.95538300	-2.69097500	0.09592200	
H	-6.06393600	0.87655600	1.32244100	
H	-7.20177500	-0.52700200	0.78614700	
H	6.16749600	0.19798400	0.90059300	
H	6.84896800	-0.25576400	-0.67563700	
H	5.96463300	1.25166400	-0.51699000	
H	4.20685600	-2.51750600	-0.88584800	
H	5.92856100	-2.24720800	-0.66614900	
H	4.85447600	-2.20276300	0.74589600	
O	3.65266100	-0.10490900	1.73839100	
H	3.03034400	0.57667600	2.02814000	
**Name**				**MB-C16-C10-OH-RAF**
Cartesian Coordinates				Frequency and Energy
C	-3.42519500	-1.22994800	-0.42827100	Zero-point correction= 0.314547 (Hartree/Particle)
C	-3.57377300	0.17517400	-0.19847000	Thermal correction to Energy= 0.334919
C	-2.39895900	0.93007700	0.02505500	Thermal correction to Enthalpy= 0.335864
C	-2.15103600	-1.78878700	-0.53131500	Thermal correction to Gibbs Free Energy= 0.263969
C	-1.15019500	0.34240700	-0.02005200	Sum of electronic and zero-point Energies= -1257.477721
S	0.22892100	1.38709000	0.29404800	Sum of electronic and thermal Energies= -1257.457349
N	0.19603100	-1.71952800	-0.39544100	Sum of electronic and thermal Enthalpies= -1257.456404
C	-0.97031000	-1.04575700	-0.30606900	Sum of electronic and thermal Free Energies= -1257.528300
C	1.57774900	0.28074500	0.08087700	
C	2.85202100	0.81620200	0.23073100	
C	3.95667800	-0.01331400	0.10829000	
C	3.81873800	-1.36967700	-0.19325200	
C	2.55355000	-1.88906900	-0.34954500	
C	1.39305000	-1.09290900	-0.21546900	
N	5.27608600	0.54202800	0.28159200	
C	5.53736800	1.87571800	-0.29670200	
C	6.18851700	-0.10254200	0.90204700	
N	-4.79989400	0.73932600	-0.15551500	
C	-5.99340600	-0.06460900	-0.39976100	
C	-4.93375700	2.17404300	0.06254400	
H	-4.27368900	-1.82471500	-0.73124800	
H	-2.04948000	-2.84127800	-0.76608800	
H	-2.47146600	1.99023500	0.23136600	
H	2.97256600	1.86803100	0.46219400	
H	4.69249800	-1.99539700	-0.32980900	
H	2.41948300	-2.93495200	-0.59686200	
H	6.60851400	2.05599900	-0.27483800	
H	5.16066500	1.88379200	-1.31696500	
H	5.02194400	2.62526200	0.30109000	
H	5.95732900	-1.05723600	1.35878100	
H	7.18163200	0.32403400	0.96935900	
H	-6.04487000	-0.90468400	0.29588600	
H	-6.86917900	0.56074900	-0.25063600	
H	-6.00907700	-0.45073700	-1.42269600	
H	-4.41972400	2.73953600	-0.71960100	
H	-5.98812000	2.43452300	0.04525400	

H -4.51903600 2.46212700 1.03204100<br>O -3.63095200 -2.00342700 1.56432100<br>H -2.88484800 -1.49958800 1.92710200	
**Name**	**MB-C16-C11-OH-RAF**
Cartesian Coordinates	Frequency and Energy
C 3.49387200 1.13283400 -0.43835200<br>C 3.62564800 -0.25998500 -0.13957400<br>C 2.44314600 -0.99027000 0.10134300<br>C 2.24054000 1.75919600 -0.39476200<br>C 1.19829700 -0.37901300 0.03086900<br>S -0.18567600 -1.41355200 0.31834700<br>N -0.12452700 1.67793100 -0.38956200<br>C 1.02905000 1.00214900 -0.26330400<br>C -1.53160800 -0.30714600 0.06940900<br>C -2.80834900 -0.83953200 0.20546400<br>C -3.91135000 -0.01511100 0.03622500<br>C -3.75696300 1.33187300 -0.30167800<br>C -2.48817300 1.84816100 -0.43441700<br>C -1.33002400 1.05889500 -0.24608300<br>N -5.23478400 -0.55843400 0.20165100<br>C -5.47313100 -1.94222200 -0.25647200<br>C -6.17717400 0.13083000 0.72298700<br>N 4.84403800 -0.83671800 -0.11102300<br>C 6.05295600 -0.05891500 -0.37711100<br>C 4.95573000 -2.26756000 0.14217500<br>H 4.36245600 1.73407000 -0.66466200<br>H 2.14947200 2.76765500 -0.77483400<br>H 2.49519000 -2.04736500 0.32569300<br>H -2.93128300 -1.88540100 0.46136300<br>H -4.62311600 1.95599300 -0.48605800<br>H -2.34946700 2.88782700 -0.70477200<br>H -6.54475700 -2.11891300 -0.27047300<br>H -5.04631600 -2.05252900 -1.25069100<br>H -4.99444100 -2.63035900 0.43822100<br>H -5.97171400 1.12243700 1.10713900<br>H -7.17148900 -0.29409200 0.77792300<br>H 6.14693500 0.76806300 0.32940000<br>H 6.91384400 -0.71104700 -0.26263200<br>H 6.04861900 0.33931600 -1.39432900<br>H 4.42079400 -2.83866000 -0.62164600<br>H 6.00421400 -2.54898600 0.12206000<br>H 4.54126300 -2.52021900 1.12160900<br>O 2.67634100 2.42308600 1.43818500<br>H 2.60633600 1.58104400 1.91441500	Zero-point correction= 0.315026 (Hartree/Particle)<br>Thermal correction to Energy= 0.335222<br>Thermal correction to Enthalpy= 0.336166<br>Thermal correction to Gibbs Free Energy= 0.264298<br>Sum of electronic and zero-point Energies= -1257.472287<br>Sum of electronic and thermal Energies= -1257.452092<br>Sum of electronic and thermal Enthalpies= -1257.451148<br>Sum of electronic and thermal Free Energies= -1257.523015
**Name**	**MB-C16-C12-OH-RAF**
Cartesian Coordinates	Frequency and Energy
C -3.54396900 -1.29629700 -0.33587100<br>C -3.71310700 0.10632500 -0.12901000<br>C -2.54152800 0.88704400 0.08076300<br>C -2.30763800 -1.86443800 -0.27409300<br>C -1.30397900 0.30219200 0.14074600<br>S 0.06827800 1.35060700 0.48747800<br>N 0.08063600 -1.77066400 -0.18398700<br>C -1.12921300 -1.12329000 0.07673000	Zero-point correction= 0.314636 (Hartree/Particle)<br>Thermal correction to Energy= 0.334925<br>Thermal correction to Enthalpy= 0.335869<br>Thermal correction to Gibbs Free Energy= 0.264693<br>Sum of electronic and zero-point Energies= -1257.468604<br>Sum of electronic and thermal Energies= -1257.448315<br>Sum of electronic and thermal Enthalpies= -1257.447371<br>Sum of electronic and thermal Free Energies= -1257.518546

C 1.41621300 0.28164400 0.16725800<br>C 2.68693200 0.84421100 0.21722700<br>C 3.79877900 0.04398600 0.00162500<br>C 3.67608500 -1.31611800 -0.30994600<br>C 2.42213700 -1.86924800 -0.36455700<br>C 1.24652500 -1.10640400 -0.11737900<br>N 5.11076500 0.63519000 0.07889200<br>C 5.27600000 2.00473800 -0.45078300<br>C 6.09506600 0.00003000 0.59104800<br>N -4.93604200 0.67802200 -0.15295800<br>C -6.12034200 -0.12935000 -0.42816000<br>C -5.07931400 2.12102400 0.00575600<br>H -4.39816900 -1.91733600 -0.56771400<br>H -2.18279000 -2.92616400 -0.45318200<br>H -2.62172500 1.96183400 0.18398100<br>H 2.79913100 1.89839000 0.44123400<br>H 4.55227100 -1.91381700 -0.52949200<br>H 2.29507300 -2.91659300 -0.60888300<br>H 6.33824900 2.20964400 -0.54693500<br>H 4.77997900 2.05931700 -1.41694200<br>H 4.82350700 2.70670300 0.24746500<br>H 5.93838600 -0.98016400 1.02495200<br>H 7.07549100 0.46071600 0.59362300<br>H -6.21957300 -0.93438000 0.30304400<br>H -6.99846400 0.50677900 -0.36381400<br>H -6.07599300 -0.56612000 -1.42927100<br>H -4.59021700 2.65848600 -0.81121600<br>H -6.13653200 2.36985600 0.00374100<br>H -4.64659200 2.45126900 0.95263100<br>O -0.97098900 -1.48382300 1.99772100<br>H -0.89596300 -2.44968200 1.96820900	
**Name**	**MB-C16-C14-OH-RAF**
Cartesian Coordinates	Frequency and Energy
C -3.54353100 -1.35805700 -0.20705800<br>C -3.66229100 0.04365100 -0.24262200<br>C -2.54288700 0.86643700 -0.18937600<br>C -2.30100100 -1.91918800 -0.12901300<br>C -1.27959600 0.30231000 -0.11242900<br>S 0.08164900 1.38993600 -0.03752500<br>N 0.07612200 -1.79448300 -0.22311300<br>C -1.10254100 -1.12849500 -0.02509000<br>C 1.44322400 0.26956900 -0.08621100<br>C 2.69650400 0.82989400 -0.04454600<br>C 3.86397200 0.02156300 -0.12978100<br>C 3.68582800 -1.38738100 -0.27931300<br>C 2.43365300 -1.92608400 -0.31606300<br>C 1.24435000 -1.14285200 -0.20656800<br>N 5.09286100 0.57034600 -0.07904700<br>C 5.25276600 2.01171000 0.08668400<br>C 6.28144500 -0.26965800 -0.19975300<br>N -4.96500900 0.64287100 -0.33342000<br>C -5.91147800 0.09895000 -1.00268400<br>C -5.18150200 1.92278000 0.37493200<br>H -4.42463800 -1.98708800 -0.21857200	Zero-point correction= 0.314920 (Hartree/Particle)<br>Thermal correction to Energy= 0.335105<br>Thermal correction to Enthalpy= 0.336049<br>Thermal correction to Gibbs Free Energy= 0.264897<br>Sum of electronic and zero-point Energies= -1257.469623<br>Sum of electronic and thermal Energies= -1257.449437<br>Sum of electronic and thermal Enthalpies= -1257.448493<br>Sum of electronic and thermal Free Energies= -1257.519646

H -2.18049900 -2.99488000 -0.09968800<br>H -2.64618700 1.94349800 -0.24164400<br>H 2.79143500 1.90492700 0.04096400<br>H 4.54524300 -2.03876400 -0.35542400<br>H 2.30642300 -2.99751300 -0.41946400<br>H 6.31271300 2.24196200 0.13900900<br>H 4.81658300 2.55289300 -0.75666600<br>H 4.77540500 2.35112700 1.00872100<br>H 6.33226100 -0.99363000 0.61698900<br>H 6.28227500 -0.80866200 -1.14974800<br>H 7.16166500 0.36504700 -0.15876400<br>H -6.88896100 0.56475400 -1.00338600<br>H -5.72640700 -0.80662900 -1.56720900<br>H -4.69016700 2.71963000 -0.18040900<br>H -6.25023000 2.10988000 0.42217300<br>H -4.75768700 1.83906700 1.37288300<br>O -1.03864900 -1.11922800 2.02477400<br>H -1.92867000 -0.78976400 2.23009100	
**Name**	**MB-C16-C16-OH-RAF**
Cartesian Coordinates	Frequency and Energy
C -3.62428900 1.54307300 -0.20859900<br>C -3.79935400 0.15319700 -0.05805300<br>C -2.68652500 -0.69288500 0.01573200<br>C -2.35905300 2.06040500 -0.25525400<br>C -1.40886100 -0.16496700 -0.01602000<br>S -0.06992300 -1.29013100 0.13166400<br>N -0.01348400 1.87055900 -0.20088300<br>C -1.19944200 1.24113400 -0.14810600<br>C 1.31539400 -0.21699100 0.02154300<br>C 2.56308700 -0.79636600 0.08663400<br>C 3.73807400 -0.00743600 0.01159800<br>C 3.58377200 1.40507300 -0.13431700<br>C 2.34218500 1.96583200 -0.19849800<br>C 1.14804800 1.19564300 -0.12537500<br>N 4.96567100 -0.57100700 0.07664100<br>C 5.10419200 -2.01390100 0.22246100<br>C 6.16489900 0.25693000 0.00481700<br>N -5.09883000 -0.39045500 0.02352600<br>C -6.08260600 0.25360300 0.58142900<br>C -5.30796100 -1.79700100 -0.36374500<br>H -4.47704000 2.19601600 -0.34839300<br>H -2.19135400 3.12023300 -0.39784900<br>H -2.81164300 -1.76127000 0.14400600<br>H 2.63480000 -1.86977700 0.19874900<br>H 4.45305800 2.04390700 -0.19586700<br>H 2.21919300 3.03604100 -0.30943800<br>H 6.15975700 -2.26806200 0.25041400<br>H 4.64098600 -2.35857400 1.15105100<br>H 4.64538600 -2.53669200 -0.62111700<br>H 6.21277700 0.79503100 -0.94510500<br>H 6.19260200 0.97698200 0.82630100<br>H 7.03907200 -0.38318400 0.08061900<br>H -7.08204900 -0.15078300 0.49285900	Zero-point correction= 0.313751 (Hartree/Particle)<br>Thermal correction to Energy= 0.333868<br>Thermal correction to Enthalpy= 0.334812<br>Thermal correction to Gibbs Free Energy= 0.263245<br>Sum of electronic and zero-point Energies= -1257.478588<br>Sum of electronic and thermal Energies= -1257.458471<br>Sum of electronic and thermal Enthalpies= -1257.457527<br>Sum of electronic and thermal Free Energies= -1257.529094

H -5.91862400 1.25376100 0.95425400<br>H -4.97066100 -2.43099500 0.45712200<br>H -6.37035500 -1.95897000 -0.52665600<br>H -4.74952300 -2.00348200 -1.27317100<br>O -5.79274200 -0.62521300 2.52680000<br>H -6.62434700 -0.55928300 3.02732000	
**Name**	**MB-C16-N13-OH-RAF**
Cartesian Coordinates	Frequency and Energy
C -3.23932600 -1.86429400 -0.14265400<br>C -3.45713600 -0.47168500 -0.11150300<br>C -2.40342300 0.41340300 -0.10383700<br>C -1.96218900 -2.35096100 -0.14982800<br>C -1.09570200 -0.08525500 -0.10462000<br>S 0.17500800 1.09076800 -0.07054200<br>N 0.38212100 -2.07493400 -0.14045500<br>C -0.82680700 -1.49041300 -0.12848700<br>C 1.59030800 0.07872000 -0.06173900<br>C 2.81602400 0.75142900 -0.00496200<br>C 4.02170100 0.02102100 -0.00026200<br>C 3.96782800 -1.40020700 -0.06436400<br>C 2.76048900 -2.03096000 -0.11010300<br>C 1.52344200 -1.32534600 -0.10685100<br>N 5.21113200 0.68336700 0.05877000<br>C 5.26629500 2.12995900 0.25107300<br>C 6.46524900 -0.05154000 -0.05742900<br>N -4.80776800 0.03386000 -0.10883500<br>C -5.69341800 -0.54528200 0.62353200<br>C -5.04384400 1.24415700 -0.85779900<br>H -4.08417000 -2.54131700 -0.18548000<br>H -1.77789400 -3.41722500 -0.18626900<br>H -2.58067000 1.48264500 -0.07404200<br>H 2.82730000 1.83450400 0.02120400<br>H 4.87636200 -1.98507100 -0.05682600<br>H 2.71199700 -3.11242300 -0.14453900<br>H 6.29949800 2.41781700 0.41298500<br>H 4.88243900 2.63526000 -0.63898000<br>H 4.66689200 2.41374700 1.11628500<br>H 6.60935900 -0.67223400 0.83168900<br>H 6.43793100 -0.69299400 -0.93876300<br>H 7.28019500 0.65934900 -0.14235400<br>H -6.71318300 -0.18557100 0.58153800<br>H -5.40897300 -1.35609100 1.28017600<br>H -4.54646300 1.16135700 -1.82342900<br>H -6.11903200 1.37748400 -0.96847900<br>H -4.55095600 2.29679100 -0.13207600<br>O -4.09337000 3.23034500 0.55246100<br>H -4.84869500 3.80070200 0.72984900	Zero-point correction= 0.307794 (Hartree/Particle)<br>Thermal correction to Energy= 0.328243<br>Thermal correction to Enthalpy= 0.329187<br>Thermal correction to Gibbs Free Energy= 0.256802<br>Sum of electronic and zero-point Energies= -1257.460793<br>Sum of electronic and thermal Energies= -1257.440344<br>Sum of electronic and thermal Enthalpies= -1257.439400<br>Sum of electronic and thermal Free Energies= -1257.511785
**Name**	**MB-C16-C17-OH-FHT**
Cartesian Coordinates	Frequency and Energy
C -3.23932600 -1.86429400 -0.14265400<br>C -3.45713600 -0.47168500 -0.11150300<br>C -2.40342300 0.41340300 -0.10383700<br>C -1.96218900 -2.35096100 -0.14982800<br>C -1.09570200 -0.08525500 -0.10462000	Zero-point correction= 0.307794 (Hartree/Particle)<br>Thermal correction to Energy= 0.328243<br>Thermal correction to Enthalpy= 0.329187<br>Thermal correction to Gibbs Free Energy= 0.256802<br>Sum of electronic and zero-point Energies= -1257.460793

S 0.17500800 1.09076800 -0.07054200<br>N 0.38212100 -2.07493400 -0.14045500<br>C -0.82680700 -1.49041300 -0.12848700<br>C 1.59030800 0.07872000 -0.06173900<br>C 2.81602400 0.75142900 -0.00496200<br>C 4.02170100 0.02102100 -0.00026200<br>C 3.96782800 -1.40020700 -0.06436400<br>C 2.76048900 -2.03096000 -0.11010300<br>C 1.52344200 -1.32534600 -0.10685100<br>N 5.21113200 0.68336700 0.05877000<br>C 5.26629500 2.12995900 0.25107300<br>C 6.46524900 -0.05154000 -0.05742900<br>N -4.80776800 0.03386000 -0.10883500<br>C -5.69341800 -0.54528200 0.62353200<br>C -5.04384400 1.24415700 -0.85779900<br>H -4.08417000 -2.54131700 -0.18548000<br>H -1.77789400 -3.41722500 -0.18626900<br>H -2.58067000 1.48264500 -0.07404200<br>H 2.82730000 1.83450400 0.02120400<br>H 4.87636200 -1.98507100 -0.05682600<br>H 2.71199700 -3.11242300 -0.14453900<br>H 6.29949800 2.41781700 0.41298500<br>H 4.88243900 2.63526000 -0.63898000<br>H 4.66689200 2.41374700 1.11628500<br>H 6.60935900 -0.67223400 0.83168900<br>H 6.43793100 -0.69299400 -0.93876300<br>H 7.28019500 0.65934900 -0.14235400<br>H -6.71318300 -0.18557100 0.58153800<br>H -5.40897300 -1.35609100 1.28017600<br>H -4.54646300 1.16135700 -1.82342900<br>H -6.11903200 1.37748400 -0.96847900<br>H -4.55095600 2.29679100 -0.13207600<br>O -4.09337000 3.23034500 0.55246100<br>H -4.84869500 3.80070200 0.72984900	Sum of electronic and thermal Energies= -1257.440344<br>Sum of electronic and thermal Enthalpies= -1257.439400<br>Sum of electronic and thermal Free Energies= -1257.511785
**Name**	**MB-C16-C19-OH-FHT**
Cartesian Coordinates	Frequency and Energy
C -3.29906600 -1.70780500 -0.12359000<br>C -3.52848000 -0.32597700 -0.36727200<br>C -2.41211900 0.52409600 -0.42806900<br>C -2.02731200 -2.18771700 0.03587600<br>C -1.12560600 0.02303300 -0.26301600<br>S 0.17930200 1.18487200 -0.35626000<br>N 0.32695800 -1.94285400 0.15546600<br>C -0.87999000 -1.35393400 -0.02377600<br>C 1.58375800 0.17519100 -0.09051500<br>C 2.82328600 0.81175100 -0.10029400<br>C 3.97085100 0.06776100 0.11264300<br>C 3.91026000 -1.31886400 0.30647200<br>C 2.68591900 -1.93890000 0.31477700<br>C 1.47542200 -1.22382300 0.12506700<br>N 5.24989100 0.73100800 0.11937200<br>C 5.43746200 1.87419200 -0.79739200<br>C 6.19605500 0.35028600 0.89053900<br>N -4.80801900 0.15764400 -0.53212800	Zero-point correction= 0.310255 (Hartree/Particle)<br>Thermal correction to Energy= 0.330467<br>Thermal correction to Enthalpy= 0.331411<br>Thermal correction to Gibbs Free Energy= 0.259837<br>Sum of electronic and zero-point Energies= -1257.477226<br>Sum of electronic and thermal Energies= -1257.457015<br>Sum of electronic and thermal Enthalpies= -1257.456070<br>Sum of electronic and thermal Free Energies= -1257.527645

C	-5.92652500	-0.66379300	-0.07218700	
C	-5.01114000	1.57743300	-0.52990300	
H	-4.12934800	-2.39704900	-0.06292200	
H	-1.86484800	-3.24310900	0.22119200	
H	-2.53309900	1.58556100	-0.60090100	
H	2.87602300	1.88287100	-0.25626300	
H	4.81710000	-1.90081500	0.41719100	
H	2.61772800	-3.01091600	0.45278000	
H	6.49618800	2.11240700	-0.83310000	
H	5.06767400	1.59055100	-1.78008800	
H	4.87961500	2.72657900	-0.41424100	
H	6.02844700	-0.45321300	1.59704400	
H	7.15547100	0.84816700	0.82873600	
H	-5.83523800	-0.89971700	0.99328400	
H	-6.84904800	-0.11327100	-0.23724900	
H	-5.98361500	-1.59320600	-0.63784100	
H	-4.41799900	2.08177700	-1.29233300	
H	-6.06408100	1.80445800	-0.67732500	
H	-4.70799600	2.02656200	0.47095900	
O	-3.98544400	2.36803900	1.90968700	
H	-3.28081900	1.70003900	1.83025000	
**Name**				**MB-C16-C20-OH-FHT**
Cartesian Coordinates				Frequency and Energy
C	-3.30251500	-1.15469500	-0.65010300	Zero-point correction= 0.310435 (Hartree/Particle)
C	-3.43898100	0.24010300	-0.41244500	Thermal correction to Energy= 0.330752
C	-2.26915200	0.98346600	-0.18393300	Thermal correction to Enthalpy= 0.331696
C	-2.06557700	-1.74338600	-0.66637600	Thermal correction to Gibbs Free Energy= 0.259331
C	-1.02149000	0.37544600	-0.20882400	Sum of electronic and zero-point Energies= -1257.476596
S	0.36087000	1.41396400	0.07008800	Sum of electronic and thermal Energies= -1257.456279
N	0.29509600	-1.71069100	-0.48172700	Sum of electronic and thermal Enthalpies= -1257.455335
C	-0.86739100	-1.01602400	-0.44963900	Sum of electronic and thermal Free Energies= -1257.527700
C	1.68946700	0.27664700	-0.00084000	
C	2.96579900	0.79203100	0.21354700	
C	4.05570400	-0.06046100	0.18294500	
C	3.90367900	-1.42529600	-0.09161200	
C	2.64309400	-1.92541600	-0.30602300	
C	1.48775100	-1.10400500	-0.26242100	
N	5.37383400	0.46778700	0.42831800	
C	5.69204100	1.80437200	-0.11470700	
C	6.24258100	-0.19877500	1.08805400	
N	-4.67583700	0.84670700	-0.40705400	
C	-5.85130300	0.02458600	-0.33301500	
C	-4.78600600	2.19257600	0.14808700	
H	-4.17317900	-1.77325900	-0.81626900	
H	-1.97517700	-2.80849000	-0.84514100	
H	-2.32573300	2.04566000	0.01385200	
H	3.09149300	1.84755000	0.42420600	
H	4.77026500	-2.07173800	-0.15959300	
H	2.50258800	-2.97562700	-0.53027300	
H	6.76579000	1.95317600	-0.04697400	
H	5.35722700	1.84238400	-1.14868800	
H	5.17518500	2.55743500	0.47789900	
H	5.97260000	-1.15329800	1.52314500	
H	7.23829300	0.20973300	1.20918700	

H -5.88874700 -0.54392500 0.65111000	
H -6.74201500 0.64707200 -0.37170200	
H -5.89098700 -0.71773500 -1.12884100	
H -4.19716000 2.90034100 -0.43605500	
H -5.82684300 2.50209800 0.10251300	
H -4.44827100 2.22367200 1.18979200	
O -5.53784600 -1.37338200 2.02234900	
H -4.63699200 -1.61374000 1.74018800	
**Name**	**MB-C17-C1-OH-RAF**
Cartesian Coordinates	Frequency and Energy
C 3.50220200 1.09011700 -0.54205700	Zero-point correction= 0.314013 (Hartree/Particle)
C 3.58045000 -0.28070500 -0.30871900	Thermal correction to Energy= 0.334773
C 2.44104200 -1.02364000 -0.06055600	Thermal correction to Enthalpy= 0.335717
C 2.25448900 1.72418100 -0.46183800	Thermal correction to Gibbs Free Energy= 0.261782
C 1.17805800 -0.40854200 -0.08663400	Sum of electronic and zero-point Energies= -1257.468959
S -0.19830600 -1.45650200 0.19462300	Sum of electronic and thermal Energies= -1257.448199
N -0.12001800 1.66763300 -0.37896900	Sum of electronic and thermal Enthalpies= -1257.447255
C 1.04062700 0.96368200 -0.32066200	Sum of electronic and thermal Free Energies= -1257.521190
C -1.53866500 -0.33989800 0.00418100	
C -2.80866100 -0.87065000 0.11811500	
C -3.95312100 -0.04838700 -0.00071600	
C -3.74360600 1.34398700 -0.24055800	
C -2.48093300 1.85293600 -0.35283500	
C -1.31151800 1.05007200 -0.24220700	
N -5.20105500 -0.55742800 0.10890700	
C -5.38844100 -1.98478000 0.33294600	
C -6.36776200 0.29885500 -0.07622400	
N 4.86291300 -0.93905700 -0.32314200	
C 5.97621800 -0.24882700 0.35761800	
C 5.02457600 -2.07709500 -0.88135100	
H 4.38387700 1.67858200 -0.76182200	
H 2.15684500 2.73633000 -0.83111900	
H 2.52299500 -2.08266200 0.16197500	
H -2.91842400 -1.93219500 0.29893900	
H -4.58902400 2.01144100 -0.33541900	
H -2.33729000 2.91135100 -0.53644200	
H -6.45182900 -2.19115400 0.41501100	
H -4.97966600 -2.57161600 -0.49486000	
H -4.90100300 -2.30147700 1.25854500	
H -6.39311600 1.09708600 0.66962900	
H -6.37319700 0.74883200 -1.07246100	
H -7.26355600 -0.30542200 0.03594300	
H 5.64532100 0.02176800 1.35843500	
H 6.82934300 -0.92005900 0.39468300	
H 6.22878400 0.64687400 -0.20703400	
H 4.20117900 -2.53991100 -1.41241800	
H 5.99275000 -2.55893800 -0.82045000	
O 2.42989600 2.33098400 1.44560100	
H 3.28328600 2.78587100 1.36882800	
**Name**	**MB-C17-C2-OH-RAF**
Cartesian Coordinates	Frequency and Energy
C -3.45575200 -1.24287500 -0.51634800	Zero-point correction= 0.314591 (Hartree/Particle)
C -3.54400200 0.15760700 -0.33252300	Thermal correction to Energy= 0.335009
C -2.41907600 0.91779700 -0.11163100	Thermal correction to Enthalpy= 0.335953

C -2.18299900 -1.80995600 -0.62036500<br>C -1.15354200 0.31882500 -0.14090100<br>S 0.21014100 1.35648400 0.18303100<br>N 0.17293400 -1.74623700 -0.49137500<br>C -1.00157500 -1.06890000 -0.41146700<br>C 1.56620600 0.25526100 -0.03148200<br>C 2.82673000 0.79729000 0.11391900<br>C 3.98405300 -0.00688000 -0.02584600<br>C 3.79367400 -1.39180600 -0.30601900<br>C 2.53585900 -1.90950600 -0.44794600<br>C 1.35811300 -1.12313300 -0.32463800<br>N 5.22462900 0.52101100 0.09958100<br>C 5.38591000 1.91300300 0.49993200<br>C 6.39506400 -0.34900200 0.07706100<br>N -4.83204100 0.80049300 -0.35807700<br>C -5.92793900 0.17350000 0.40803300<br>C -5.00750400 1.89680500 -0.99203600<br>H -4.31925500 -1.81123600 -0.83315200<br>H -2.08893100 -2.86704100 -0.83520600<br>H -2.51484100 1.97864500 0.09605800<br>H 2.92226700 1.85193400 0.33779500<br>H 4.64637600 -2.04839000 -0.40826500<br>H 2.40562600 -2.96394900 -0.66309200<br>H 6.44656700 2.14511400 0.53994900<br>H 4.91492900 2.58334200 -0.22261200<br>H 4.94787300 2.09776300 1.48587600<br>H 6.37681900 -1.06524700 0.90421400<br>H 6.45265500 -0.90132900 -0.86269800<br>H 7.28690700 0.26526400 0.16600600<br>H -5.61513600 0.10074600 1.44717000<br>H -6.81313300 0.79490700 0.30690700<br>H -6.11881300 -0.81979000 0.00712800<br>H -4.19526800 2.31939600 -1.57177900<br>H -5.97532100 2.38082900 -0.94564600<br>O -3.78413900 -1.96395900 1.44846900<br>H -2.94965500 -1.63018800 1.81783000	Thermal correction to Gibbs Free Energy= 0.263860<br>Sum of electronic and zero-point Energies= -1257.474672<br>Sum of electronic and thermal Energies= -1257.454254<br>Sum of electronic and thermal Enthalpies= -1257.453310<br>Sum of electronic and thermal Free Energies= -1257.525403
**Name**	**MB-C17-C3-OH-RAF**
Cartesian Coordinates	Frequency and Energy
C 3.36277900 1.46892000 -0.25821300<br>C 3.52759300 0.08816100 0.03401400<br>C 2.37698900 -0.72898400 0.16464600<br>C 2.10642400 2.00865800 -0.25468800<br>C 1.12196200 -0.16036300 0.19028000<br>S -0.23053500 -1.22904800 0.56218200<br>N -0.25433100 1.89304600 -0.05621700<br>C 0.94031300 1.23097000 -0.02529900<br>C -1.59906400 -0.18910400 0.18677300<br>C -2.84303700 -0.77362300 0.16834400<br>C -4.01455900 0.00518000 -0.02303000<br>C -3.85459900 1.41599300 -0.19131200<br>C -2.61351800 1.98059200 -0.18213000<br>C -1.41775000 1.22181300 -0.00719700<br>N -5.23671100 -0.56243800 -0.04244200<br>C -5.38182200 -2.00328900 0.13800900	Zero-point correction= 0.315093 (Hartree/Particle)<br>Thermal correction to Energy= 0.335173<br>Thermal correction to Enthalpy= 0.336117<br>Thermal correction to Gibbs Free Energy= 0.265340<br>Sum of electronic and zero-point Energies= -1257.462690<br>Sum of electronic and thermal Energies= -1257.442610<br>Sum of electronic and thermal Enthalpies= -1257.441666<br>Sum of electronic and thermal Free Energies= -1257.512443

C -6.43238000 0.25405900 -0.23912800<br>N 4.77267700 -0.54846300 -0.36596200<br>C 6.05078000 0.00974200 0.12595900<br>C 4.76667800 -1.56543600 -1.13848200<br>H 4.22566600 2.08893100 -0.46257700<br>H 1.96914100 3.06487800 -0.45057500<br>H 2.50585400 -1.79368900 0.33016300<br>H -2.92391400 -1.84322400 0.31344500<br>H -4.72154700 2.04672200 -0.33191300<br>H -2.50168500 3.04941100 -0.32043600<br>H -6.43678800 -2.25687200 0.09972700<br>H -4.85982200 -2.54728400 -0.65287600<br>H -4.97985600 -2.31499600 1.10490900<br>H -6.53815500 0.98822600 0.56267100<br>H -6.39178300 0.77785300 -1.19643100<br>H -7.30175000 -0.39652800 -0.23448500<br>H 6.20954900 -0.34373100 1.14074700<br>H 6.84349100 -0.32795800 -0.53578600<br>H 5.99296700 1.09448000 0.11930500<br>H 3.83179100 -1.95532100 -1.52217600<br>H 5.71197000 -2.02083500 -1.40858300<br>O 4.00049800 0.14380900 1.95928900<br>H 3.15370200 0.48128600 2.28944700		
**Name**	**MB-C17-C4-OH-RAF**	
Cartesian Coordinates	Frequency and Energy	
C 3.54116100 1.49908000 0.53339100<br>C 3.69278200 0.12290800 0.41238200<br>C 2.56498800 -0.72227300 0.37415300<br>C 2.26884100 2.03345900 0.60908800<br>C 1.28147400 -0.15668500 0.37955500<br>S -0.05662400 -1.26021900 0.20181000<br>N -0.09119100 1.88605900 0.61910000<br>C 1.09676800 1.24026700 0.54248400<br>C -1.43771700 -0.18553700 0.38383600<br>C -2.68569500 -0.77235200 0.33798500<br>C -3.86056000 0.00805800 0.46767600<br>C -3.70269400 1.41466400 0.64414700<br>C -2.45739400 1.97712200 0.68493800<br>C -1.26229300 1.21746900 0.56040500<br>N -5.08714400 -0.55970200 0.42613300<br>C -5.21976300 -2.00466400 0.29182900<br>C -6.28086200 0.25354700 0.62886200<br>N 5.00422400 -0.45260900 0.36521200<br>C 6.11138000 0.40648800 -0.10913100<br>C 5.23947400 -1.65696200 0.73676600<br>H 4.39683700 2.15787900 0.58806800<br>H 2.14055400 3.10282900 0.72385100<br>H 2.67684900 -1.76613800 0.10720400<br>H -2.75908300 -1.84390200 0.20455700<br>H -4.57158800 2.04966200 0.74743100<br>H -2.35052300 3.04732900 0.82017200<br>H -6.27561900 -2.25872600 0.26507700<br>H -4.75643400 -2.35592300 -0.63343600<br>H -4.75501100 -2.52525900 1.13437600	Zero-point correction= 0.314697 (Hartree/Particle)<br>Thermal correction to Energy= 0.334991<br>Thermal correction to Enthalpy= 0.335935<br>Thermal correction to Gibbs Free Energy= 0.263921<br>Sum of electronic and zero-point Energies= -1257.477110<br>Sum of electronic and thermal Energies= -1257.456816<br>Sum of electronic and thermal Enthalpies= -1257.455872<br>Sum of electronic and thermal Free Energies= -1257.527886	

H -6.26811600 0.73847000 1.60882200<br>H -6.36826500 1.02350000 -0.14148000<br>H -7.15424800 -0.39013900 0.57200800<br>H 6.35321400 1.12845700 0.66862200<br>H 6.96959200 -0.22775300 -0.30716800<br>H 5.79253200 0.91541200 -1.01539800<br>H 6.24857100 -2.03987700 0.65029300<br>H 4.44553000 -2.26979300 1.14097300<br>O 2.74086300 -1.28333700 2.46597300<br>H 2.36181900 -0.45511600 2.80659100	
**Name**	**MB-C17-C5-OH-RAF**
Cartesian Coordinates	Frequency and Energy
C 3.52267000 1.41773400 -0.00762000<br>C 3.63722800 0.02396500 -0.06602200<br>C 2.51590900 -0.77402100 -0.18496500<br>C 2.27001900 2.00586400 -0.02446300<br>C 1.23277500 -0.18895400 -0.13749500<br>S -0.08039700 -1.15896100 -0.82176100<br>N -0.09722800 1.91093000 -0.09108400<br>C 1.09113200 1.24894700 -0.11008600<br>C -1.44990000 -0.16019300 -0.33678700<br>C -2.68990100 -0.74438600 -0.30024300<br>C -3.85602200 0.02848300 -0.03374200<br>C -3.68885300 1.42843400 0.20038600<br>C -2.44808100 1.99225200 0.16943000<br>C -1.26133500 1.24124800 -0.08954400<br>N -5.07530300 -0.54130900 -0.00761500<br>C -5.23012700 -1.97277800 -0.25394000<br>C -6.26441200 0.26461200 0.26295200<br>N 4.93910300 -0.58940500 -0.03323200<br>C 6.03886400 0.10767000 -0.72988500<br>C 5.13908500 -1.70190300 0.56474300<br>H 4.40304200 2.04309200 0.06668200<br>H 2.17913500 3.08349500 0.02778800<br>H 2.60664300 -1.84753900 -0.31174300<br>H -2.77804700 -1.80607800 -0.49126100<br>H -4.54853400 2.05066800 0.40683400<br>H -2.32907700 3.05342800 0.35360000<br>H -6.28453500 -2.22513300 -0.20014100<br>H -4.85444900 -2.23584400 -1.24515400<br>H -4.68938100 -2.55385500 0.49636400<br>H -6.19558500 0.73543200 1.24566900<br>H -6.38539400 1.04098300 -0.49540100<br>H -7.13561800 -0.38296500 0.24504100<br>H 6.31007500 0.99399500 -0.15926300<br>H 6.88811800 -0.56649200 -0.79293500<br>H 5.69121100 0.39067400 -1.72105000<br>H 6.12163100 -2.15448500 0.51776300<br>H 4.33169500 -2.16967000 1.11493200<br>O 0.81932200 -0.44360800 1.82124300<br>H 1.52606900 0.09054400 2.21693200	Zero-point correction= 0.314974 (Hartree/Particle)<br>Thermal correction to Energy= 0.335072<br>Thermal correction to Enthalpy= 0.336016<br>Thermal correction to Gibbs Free Energy= 0.265759<br>Sum of electronic and zero-point Energies= -1257.465894<br>Sum of electronic and thermal Energies= -1257.445796<br>Sum of electronic and thermal Enthalpies= -1257.444852<br>Sum of electronic and thermal Free Energies= -1257.515109
**Name**	**MB-C17-C7-OH-RAF**
Cartesian Coordinates	Frequency and Energy
C 3.53086400 1.46469900 -0.09835300	Zero-point correction= 0.315391 (Hartree/Particle)

C 3.69531600 0.05437600 -0.14304100<br>C 2.51719400 -0.75318800 -0.22942700<br>C 2.27501900 2.01869900 -0.11789600<br>C 1.23083200 -0.17469500 -0.14247200<br>S -0.09618900 -1.16722100 -0.75304700<br>N -0.09399800 1.91449900 -0.12689300<br>C 1.07438100 1.26230800 -0.16152200<br>C -1.45376800 -0.15393400 -0.26137800<br>C -2.70587600 -0.74474200 -0.17645500<br>C -3.81702300 0.04061900 0.10705200<br>C -3.69369300 1.41867000 0.28761900<br>C -2.44217100 1.99273600 0.21306400<br>C -1.27921000 1.23769000 -0.05727800<br>N -5.11177500 -0.58336300 0.20557900<br>C -5.26485900 -1.73284900 0.74409400<br>C -6.26373600 0.15568300 -0.34945400<br>N 4.90582700 -0.52851000 -0.13636000<br>C 6.10746300 0.30122400 -0.11273800<br>C 5.05265900 -1.98444600 -0.15542400<br>H 4.39512100 2.11168900 -0.05573900<br>H 2.17079000 3.09771200 -0.09360100<br>H 2.59382400 -1.82643200 -0.34353400<br>H -2.82150100 -1.80733900 -0.36245500<br>H -4.55637100 2.03413400 0.50651500<br>H -2.32616600 3.05877100 0.36633900<br>H -6.24854000 -2.18579000 0.74780100<br>H -4.41700800 -2.23305200 1.19607100<br>H -6.48671400 0.99822400 0.30252800<br>H -5.99614100 0.51021200 -1.34232700<br>H -7.11656500 -0.51561300 -0.39216200<br>H 6.13691700 0.90281300 0.79861800<br>H 6.97908500 -0.34470700 -0.14221800<br>H 6.12619100 0.96760100 -0.97763300<br>H 4.67984500 -2.39847400 -1.09446400<br>H 6.10685600 -2.22449000 -0.05887200<br>H 4.51118000 -2.43451800 0.67776600<br>O 1.20824200 -0.64794900 1.75962300<br>H 1.80695500 0.01907500 2.13097200	Thermal correction to Energy= 0.335326<br>Thermal correction to Enthalpy= 0.336271<br>Thermal correction to Gibbs Free Energy= 0.266114<br>Sum of electronic and zero-point Energies= -1257.468030<br>Sum of electronic and thermal Energies= -1257.448094<br>Sum of electronic and thermal Enthalpies= -1257.447150<br>Sum of electronic and thermal Free Energies= -1257.517307
**Name**	**MB-C17-C8-OH-RAF**
Cartesian Coordinates	Frequency and Energy
C 3.57837300 1.45973600 -0.11088200<br>C 3.76942400 0.04736400 -0.01632600<br>C 2.60894800 -0.77686000 0.08490600<br>C 2.32480600 1.99683600 -0.14121400<br>C 1.33506200 -0.21099100 -0.00934600<br>S -0.00714000 -1.30803400 0.05004600<br>N -0.03374500 1.83870600 -0.13272800<br>C 1.14578300 1.19797000 -0.09214300<br>C -1.37354700 -0.22958000 -0.06702100<br>C -2.63720500 -0.79022800 -0.06337300<br>C -3.77184200 0.02799300 -0.10679700<br>C -3.63102600 1.43388800 -0.12392000<br>C -2.38296300 1.98727400 -0.13940300<br>C -1.19863500 1.19194200 -0.11841500	Zero-point correction= 0.314525 (Hartree/Particle)<br>Thermal correction to Energy= 0.334874<br>Thermal correction to Enthalpy= 0.335818<br>Thermal correction to Gibbs Free Energy= 0.264433<br>Sum of electronic and zero-point Energies= -1257.482109<br>Sum of electronic and thermal Energies= -1257.461760<br>Sum of electronic and thermal Enthalpies= -1257.460815<br>Sum of electronic and thermal Free Energies= -1257.532201

N -5.05781900 -0.55127700 -0.14760200<br>C -5.29101200 -1.74524100 -0.60421500<br>C -6.19473500 0.25286500 0.35335200<br>N 4.99759700 -0.49618800 0.01953600<br>C 6.18350700 0.33857400 -0.14068700<br>C 5.16757600 -1.92535800 0.27264000<br>H 4.43353800 2.11929100 -0.14889600<br>H 2.17971100 3.06841800 -0.20217700<br>H 2.70427100 -1.84968500 0.00972200<br>H -2.73607700 -1.86532100 0.03530600<br>H -4.49674200 2.08142300 -0.15669900<br>H -2.24638700 3.06061100 -0.17263900<br>H -6.28738100 -2.15463500 -0.52362800<br>H -4.49738600 -2.30755900 -1.07335300<br>H -5.91050000 0.72026600 1.29314500<br>H -6.44774200 1.01172900 -0.38590400<br>H -7.04595000 -0.40532500 0.50237400<br>H 6.32822500 0.99169200 0.72472600<br>H 7.05287600 -0.30554100 -0.23912800<br>H 6.10749100 0.94790000 -1.04300900<br>H 4.88716100 -2.51611800 -0.60480900<br>H 6.21312500 -2.11763800 0.49813100<br>H 4.56235400 -2.22118800 1.13087400<br>O 2.64643600 -1.16822100 2.20745900<br>H 2.58790000 -0.25576500 2.53252100		
**Name**	**MB-C17-C9-OH-RAF**	
Cartesian Coordinates	Frequency and Energy	
C 3.37412300 1.50361400 -0.17647900<br>C 3.58487600 0.06102000 -0.16017500<br>C 2.38755600 -0.73859100 -0.22877800<br>C 2.13150200 2.03364300 -0.08766100<br>C 1.11117500 -0.14529600 -0.11426900<br>S -0.21520800 -1.25748400 -0.10477700<br>N -0.25669000 1.90323700 0.06443400<br>C 0.93981200 1.23929800 -0.03186700<br>C -1.57739900 -0.18639400 0.01067300<br>C -2.84358000 -0.77654700 0.02232400<br>C -3.96306800 0.02226900 0.11757700<br>C -3.85237000 1.42688800 0.17164700<br>C -2.61447900 2.00366300 0.16019400<br>C -1.41320400 1.23412000 0.08055300<br>N -5.26568400 -0.58763100 0.14854700<br>C -5.47959000 -1.68466400 0.77052100<br>C -6.35486600 0.10214700 -0.57329400<br>N 4.77715100 -0.44409400 -0.65417700<br>C 6.00344300 0.19489500 -0.16996700<br>C 4.91371300 -1.90064000 -0.66107700<br>H 4.22678700 2.16309700 -0.23986900<br>H 2.00095100 3.10976400 -0.07858900<br>H 2.46287900 -1.81488300 -0.29730900<br>H -2.94342200 -1.85258600 -0.07174300<br>H -4.73590100 2.04807200 0.24039500<br>H -2.51041000 3.08010800 0.21360500	Zero-point correction= 0.314871 (Hartree/Particle)<br>Thermal correction to Energy= 0.334697<br>Thermal correction to Enthalpy= 0.335641<br>Thermal correction to Gibbs Free Energy= 0.265956<br>Sum of electronic and zero-point Energies= -1257.462157<br>Sum of electronic and thermal Energies= -1257.442330<br>Sum of electronic and thermal Enthalpies= -1257.441386<br>Sum of electronic and thermal Free Energies= -1257.511072	

H -6.46437600 -2.13237200 0.72131700<br>H -4.68282000 -2.14454700 1.34235900<br>H -6.63787400 0.99155500 -0.01325700<br>H -5.99040200 0.37882200 -1.56013300<br>H -7.20036600 -0.57546400 -0.64646200<br>H 6.19579900 -0.06503900 0.87639500<br>H 6.83539100 -0.15706900 -0.77825100<br>H 5.95849500 1.27608800 -0.26390400<br>H 4.17759800 -2.35901900 -1.31995300<br>H 5.90112300 -2.14573200 -1.04779900<br>H 4.81687000 -2.32356400 0.34543300<br>O 3.66201300 -0.34686400 1.67817500<br>H 3.05058300 0.29552900 2.06480100	
**Name**	**MB-C17-C10-OH-RAF**
Cartesian Coordinates	Frequency and Energy
C -3.42842400 -1.21129400 -0.19161800<br>C -3.55101500 0.21503500 -0.22964900<br>C -2.36188400 0.97570700 -0.17953700<br>C -2.16408800 -1.80238400 -0.22348900<br>C -1.12240100 0.36709700 -0.13513300<br>S 0.27788300 1.42794300 -0.05540500<br>N 0.18539600 -1.75267700 -0.14893800<br>C -0.96857500 -1.05215600 -0.16220200<br>C 1.60193200 0.27360000 0.00061000<br>C 2.88552100 0.79875500 0.09468200<br>C 3.97488200 -0.05951900 0.12521900<br>C 3.81486900 -1.44308600 0.03031700<br>C 2.53929600 -1.95440700 -0.05727700<br>C 1.39349400 -1.12627100 -0.06919300<br>N 5.29877600 0.49786000 0.25060900<br>C 5.54039400 1.46972600 1.04443300<br>C 6.36446100 -0.09556300 -0.58134100<br>N -4.76822600 0.79847200 -0.26376000<br>C -5.97838600 -0.01506600 -0.31946800<br>C -4.87633000 2.24729000 -0.37981600<br>H -4.29314600 -1.83418400 -0.36704200<br>H -2.08193200 -2.88214700 -0.26400200<br>H -2.41478600 2.05701300 -0.17335600<br>H 3.03647600 1.87298900 0.11899200<br>H 4.66763000 -2.10871700 0.04920000<br>H 2.38407300 -3.02478700 -0.11352600<br>H 6.53904500 1.88803700 1.07361700<br>H 4.75096900 1.85384400 1.67951000<br>H 6.59542100 -1.08826800 -0.19972900<br>H 6.00112800 -0.16130100 -1.60458400<br>H 7.24184700 0.54117800 -0.51844300<br>H -5.99834000 -0.73317100 0.50196700<br>H -6.84036000 0.63942300 -0.22319600<br>H -6.05246000 -0.55703400 -1.26684400<br>H -4.36588700 2.60708500 -1.27767900<br>H -5.92684600 2.51620500 -0.44439200<br>H -4.44168300 2.74312600 0.49178000<br>O -3.53974200 -1.46967200 1.91745500<br>H -3.51969400 -2.43946400 1.91300700	Zero-point correction= 0.313818 (Hartree/Particle)<br>Thermal correction to Energy= 0.334444<br>Thermal correction to Enthalpy= 0.335389<br>Thermal correction to Gibbs Free Energy= 0.262105<br>Sum of electronic and zero-point Energies= -1257.477550<br>Sum of electronic and thermal Energies= -1257.456924<br>Sum of electronic and thermal Enthalpies= -1257.455980<br>Sum of electronic and thermal Free Energies= -1257.529263

Name	MB-C17-C11-OH-RAF
Cartesian Coordinates	Frequency and Energy
C 3.50457200 1.16885600 -0.28788200<br>C 3.59652100 -0.22795500 -0.18092600<br>C 2.43782700 -1.01111200 0.06117400<br>C 2.28136500 1.80470500 -0.02604100<br>C 1.19779900 -0.37467000 0.09464100<br>S -0.19298900 -1.42410900 0.28441400<br>N -0.09999600 1.71558400 -0.15564000<br>C 1.07135100 1.01716500 -0.04070900<br>C -1.50848800 -0.29575800 0.14566400<br>C -2.80047800 -0.81912300 0.22166700<br>C -3.88455100 0.02504600 0.08700200<br>C -3.71514900 1.40080500 -0.16050100<br>C -2.44997100 1.91353200 -0.23180600<br>C -1.28764400 1.10057200 -0.07754800<br>N -5.21494600 -0.51582200 0.18386300<br>C -5.51073800 -1.42133800 1.03706300<br>C -6.23182300 0.01344500 -0.74859800<br>N 4.80318100 -0.86779600 -0.31543500<br>C 6.04597800 -0.11189700 -0.31599800<br>C 4.90242600 -2.31153000 -0.49010400<br>H 4.37064600 1.76550800 -0.53390600<br>H 2.16723400 2.83794900 -0.32301800<br>H 2.50791000 -2.07968400 0.21359500<br>H -2.94851100 -1.88549200 0.35776700<br>H -4.57092000 2.05379700 -0.27064400<br>H -2.29560700 2.97135900 -0.40411400<br>H -6.51180100 -1.83521200 1.03813900<br>H -4.76601600 -1.75388200 1.74990300<br>H -6.51002900 1.01715900 -0.43292800<br>H -5.80107200 0.03761600 -1.74705200<br>H -7.09812400 -0.63988900 -0.71486500<br>H 6.00290100 0.67801900 0.43116600<br>H 6.86507400 -0.79170700 -0.09668800<br>H 6.20413000 0.33337200 -1.30432600<br>H 4.10188000 -2.67520400 -1.12953100<br>H 5.86342700 -2.53303200 -0.94713300<br>H 4.85094500 -2.80568000 0.48537100<br>O 2.32252100 2.38274400 1.86510300<br>H 2.45122800 1.51676000 2.26875000	Zero-point correction= 0.314047 (Hartree/Particle)<br>Thermal correction to Energy= 0.334514<br>Thermal correction to Enthalpy= 0.335458<br>Thermal correction to Gibbs Free Energy= 0.262925<br>Sum of electronic and zero-point Energies= -1257.444391<br>Sum of electronic and thermal Energies= -1257.423924<br>Sum of electronic and thermal Enthalpies= -1257.422980<br>Sum of electronic and thermal Free Energies= -1257.495513
**Name**	**MB-C17-C12-OH-RAF**
Cartesian Coordinates	Frequency and Energy
C -3.54405400 -1.33707300 -0.18371200<br>C -3.70292000 0.08165100 -0.16104000<br>C -2.52916400 0.87363400 -0.01669800<br>C -2.31586900 -1.90249600 -0.02020200<br>C -1.29968700 0.29182200 0.14782800<br>S 0.07457600 1.36426400 0.40200300<br>N 0.07056800 -1.81316200 0.09440300<br>C -1.13719200 -1.13206600 0.25950000<br>C 1.41765500 0.25807400 0.21616400<br>C 2.69227300 0.81273400 0.19434400<br>C 3.79821400 -0.01188200 0.04727200	Zero-point correction= 0.314563 (Hartree/Particle)<br>Thermal correction to Energy= 0.334855<br>Thermal correction to Enthalpy= 0.335799<br>Thermal correction to Gibbs Free Energy= 0.264297<br>Sum of electronic and zero-point Energies= -1257.468690<br>Sum of electronic and thermal Energies= -1257.448398<br>Sum of electronic and thermal Enthalpies= -1257.447454<br>Sum of electronic and thermal Free Energies= -1257.518956

C	3.66741200	-1.39699000	-0.11270200	
C	2.40978600	-1.94440300	-0.08400100	
C	1.23975600	-1.15203200	0.08448500	
N	5.11318500	0.57644500	0.02898200	
C	5.42561400	1.54444000	0.80339200	
C	6.08968600	0.01390200	-0.92606700	
N	-4.91813600	0.65463200	-0.29542200	
C	-6.09995900	-0.17206700	-0.51826200	
C	-5.04795100	2.10650700	-0.35018800	
H	-4.39840600	-1.97585800	-0.35947900	
H	-2.19759800	-2.97908400	-0.06348100	
H	-2.60016400	1.95341900	-0.04895500	
H	2.81888000	1.88839400	0.25795900	
H	4.53625700	-2.03001500	-0.23320500	
H	2.27535700	-3.01411900	-0.18572000	
H	6.41342700	1.98218900	0.72561800	
H	4.70850800	1.90534500	1.53066100	
H	6.39822700	-0.96821300	-0.57322300	
H	5.60740000	-0.06915800	-1.89759100	
H	6.94603900	0.67964600	-0.97157900	
H	-6.22802400	-0.89128600	0.29310500	
H	-6.97389100	0.47212300	-0.55004700	
H	-6.02863600	-0.71650700	-1.46380600	
H	-4.53060700	2.51657800	-1.22208400	
H	-6.10197100	2.36026600	-0.41674200	
H	-4.63665800	2.56622000	0.55097800	
O	-1.00960300	-1.26609900	2.21444600	
H	-0.93934200	-2.22919700	2.29774700	
**Name**				**MB-C17-C14-OH-RAF**
Cartesian Coordinates				Frequency and Energy
C	-3.56091800	-1.31345600	-0.38311500	Zero-point correction= 0.314618 (Hartree/Particle)
C	-3.67514300	0.08223400	-0.26958800	Thermal correction to Energy= 0.334956
C	-2.55466300	0.89166200	-0.09916300	Thermal correction to Enthalpy= 0.335900
C	-2.31878600	-1.88220200	-0.33782200	Thermal correction to Gibbs Free Energy= 0.264125
C	-1.29663500	0.32296400	-0.04640300	Sum of electronic and zero-point Energies= -1257.469649
S	0.05892500	1.37639200	0.25691500	Sum of electronic and thermal Energies= -1257.449311
N	0.05839000	-1.76002000	-0.32572900	Sum of electronic and thermal Enthalpies= -1257.448367
C	-1.12529200	-1.11250400	-0.10163700	Sum of electronic and thermal Free Energies= -1257.520142
C	1.42248100	0.27953700	0.03215900	
C	2.67468600	0.83758800	0.11747200	
C	3.84327300	0.04910500	-0.07211300	
C	3.66719900	-1.33764500	-0.36386600	
C	2.41598200	-1.87451800	-0.44217500	
C	1.22621400	-1.11087600	-0.24234100	
N	5.07074400	0.59676300	0.01379400	
C	5.22649800	2.01743700	0.31077100	
C	6.25971000	-0.21869700	-0.21964100	
N	-4.97470300	0.69022600	-0.34163200	
C	-6.10684000	-0.05234000	0.25246900	
C	-5.15975100	1.82821600	-0.89854400	
H	-4.43295400	-1.93228600	-0.54646300	
H	-2.19579300	-2.95128400	-0.46148100	
H	-2.66909900	1.96418000	0.01510900	

H 2.76699500 1.89636700 0.32285300	
H 4.52654100 -1.97430800 -0.52102900	
H 2.29102500 -2.92925100 -0.65850700	
H 6.28594900 2.25123100 0.35720800	
H 4.76457600 2.63180700 -0.46611700	
H 4.77153000 2.26456200 1.27270300	
H 6.32325400 -1.03233400 0.50661500	
H 6.24863600 -0.64386000 -1.22591700	
H 7.13943500 0.40965900 -0.11607600	
H -5.80011600 -0.42368900 1.22743700	
H -6.95047100 0.62570800 0.34331800	
H -6.36574400 -0.88118100 -0.40368100	
H -4.33468200 2.33372000 -1.38464000	
H -6.15081300 2.26394700 -0.88431000	
O -1.20094600 -1.25249400 1.94062400	
H -1.20713400 -2.22244200 1.97550400	
**Name**	**MB-C17-C17-OH-RAF**
Cartesian Coordinates	Frequency and Energy
C -3.62761000 1.29873300 0.22867700<br>C -3.73083600 -0.09475800 0.03715100<br>C -2.60558100 -0.87941500 -0.07638700<br>C -2.39231300 1.87795000 0.31745900<br>C -1.34075700 -0.28766900 0.01254700<br>S 0.02282800 -1.34399200 -0.14915700<br>N -0.03346800 1.79965900 0.29412800<br>C -1.18976800 1.12120600 0.20941100<br>C 1.34941200 -0.22442900 -0.01492200<br>C 2.62692000 -0.78581200 -0.11309500<br>C 3.76995900 0.03512800 -0.02314200<br>C 3.59609200 1.43671200 0.16127400<br>C 2.34031000 1.95705600 0.26016800<br>C 1.16588400 1.15567800 0.17991900<br>N 5.01228400 -0.51831200 -0.11705900<br>C 5.19598000 -1.96004800 -0.26427600<br>C 6.19075200 0.34129000 -0.10199200<br>N -5.02665700 -0.70480300 -0.05737100<br>C -6.04685500 -0.02139000 -0.87089700<br>C -5.31233800 -1.76496000 0.61659200<br>H -4.51741800 1.90748800 0.32602200<br>H -2.29463900 2.94445300 0.47658200<br>H -2.69912100 -1.94479500 -0.25783800<br>H 2.72239500 -1.85290600 -0.26938500<br>H 4.45022400 2.09480400 0.22836000<br>H 2.20236300 3.02153300 0.40534800<br>H 6.24828100 -2.19000600 -0.14040300<br>H 4.61554200 -2.49177300 0.48904000<br>H 4.86779900 -2.27050200 -1.26038000<br>H 6.09936900 1.10604700 -0.87619500<br>H 6.26933100 0.83295200 0.87136500<br>H 7.07163200 -0.26348000 -0.28080400<br>H -5.62007400 0.20660500 -1.84602300<br>H -6.90250500 -0.68350600 -0.97276100<br>H -6.35519900 0.89735900 -0.37368300<br>H -4.54938900 -2.23488600 1.22023100	Zero-point correction= 0.313051 (Hartree/Particle)<br>Thermal correction to Energy= 0.333820<br>Thermal correction to Enthalpy= 0.334764<br>Thermal correction to Gibbs Free Energy= 0.259998<br>Sum of electronic and zero-point Energies= -1257.477639<br>Sum of electronic and thermal Energies= -1257.456870<br>Sum of electronic and thermal Enthalpies= -1257.455926<br>Sum of electronic and thermal Free Energies= -1257.530693

H -6.26889400 -2.24268500 0.46788900<br>O -6.46902700 -1.06819500 2.51370200<br>H -6.15283400 -1.35313600 3.37710000	
**Name**	**MB-C17-N13-OH-RAF**
Cartesian Coordinates	Frequency and Energy
C 3.60455800 1.19927300 -0.35737000<br>C 3.77367000 -0.19190700 -0.12625100<br>C 2.62540800 -0.99344500 0.05644400<br>C 2.35590600 1.75416500 -0.34546700<br>C 1.37124100 -0.41960400 0.08469200<br>S -0.00288900 -1.49537100 0.27299300<br>N -0.00421900 1.60805600 -0.03874400<br>C 1.20078700 0.97343100 -0.09867800<br>C -1.36634500 -0.42744000 0.02228600<br>C -2.61494000 -0.99577100 -0.03618400<br>C -3.78163700 -0.18450200 -0.09493100<br>C -3.61835700 1.24283600 -0.08108900<br>C -2.37953800 1.79668200 -0.03144700<br>C -1.20574100 0.99358300 -0.00244000<br>N -4.99964900 -0.73069600 -0.15203500<br>C -5.15980800 -2.18410900 -0.18272600<br>C -6.19848000 0.10992600 -0.17012400<br>N 5.03587000 -0.74699600 -0.09275900<br>C 6.17472200 0.06591400 -0.55163800<br>C 5.29491900 -1.98295800 0.35618800<br>H 4.45971800 1.83806900 -0.52661500<br>H 2.23005400 2.81865600 -0.50431500<br>H 2.71192600 -2.07037700 0.12503400<br>H -2.70655700 -2.07311900 -0.00079900<br>H -4.48467900 1.88719600 -0.12558600<br>H -2.25580900 2.87145200 -0.03895600<br>H -6.21719600 -2.41578200 -0.26155600<br>H -4.63916700 -2.60788900 -1.04358400<br>H -4.76442000 -2.63344300 0.73083200<br>H -6.23894400 0.74072100 0.71932800<br>H -6.21317200 0.74183200 -1.06014700<br>H -7.07070000 -0.53579300 -0.18263900<br>H 6.36672000 0.87089100 0.15775200<br>H 7.04753100 -0.57680100 -0.61145500<br>H 5.96021000 0.48173400 -1.53457700<br>H 4.52198900 -2.56510000 0.82945800<br>H 6.30431900 -2.35169300 0.27411300<br>O -0.00459000 2.85862700 1.13119600<br>H 0.21327300 3.61031200 0.56059900	Zero-point correction= 0.313766 (Hartree/Particle)<br>Thermal correction to Energy= 0.333926<br>Thermal correction to Enthalpy= 0.334870<br>Thermal correction to Gibbs Free Energy= 0.264234<br>Sum of electronic and zero-point Energies= -1257.441979<br>Sum of electronic and thermal Energies= -1257.421819<br>Sum of electronic and thermal Enthalpies= -1257.420875<br>Sum of electronic and thermal Free Energies= -1257.491510
**Name**	**MB-C17-C16-OH-FHT**
Cartesian Coordinates	Frequency and Energy
C -3.31919100 -1.29680200 -1.11014200<br>C -3.46759700 0.00936100 -0.60476700<br>C -2.38548500 0.74906500 -0.19418300<br>C -2.06809900 -1.83753500 -1.21301400<br>C -1.10273300 0.19282000 -0.29238600<br>S 0.21227300 1.18046900 0.24850900<br>N 0.27520900 -1.76252400 -0.94032900<br>C -0.90095300 -1.12656000 -0.80650600	Zero-point correction= 0.307861 (Hartree/Particle)<br>Thermal correction to Energy= 0.328442<br>Thermal correction to Enthalpy= 0.329386<br>Thermal correction to Gibbs Free Energy= 0.256069<br>Sum of electronic and zero-point Energies= -1257.459092<br>Sum of electronic and thermal Energies= -1257.438511<br>Sum of electronic and thermal Enthalpies= -1257.437567<br>Sum of electronic and thermal Free Energies= -1257.510884

C 1.57311300 0.13190900 -0.02989800<br>C 2.82203500 0.65732400 0.31780900<br>C 3.98956900 -0.11149000 0.13293300<br>C 3.87045600 -1.42379000 -0.40733400<br>C 2.64150300 -1.91112800 -0.73885400<br>C 1.44296600 -1.16071500 -0.56752900<br>N 5.20208700 0.40974400 0.47282900<br>C 5.32379700 1.76668600 0.99963600<br>C 6.41185000 -0.38617400 0.30014400<br>N -4.79172800 0.57442900 -0.48565800<br>C -5.67500900 -0.11423800 0.42377900<br>C -5.03870400 1.71056900 -1.03112900<br>H -4.19084600 -1.85468700 -1.42970600<br>H -1.92818400 -2.83441400 -1.61163600<br>H -2.52838800 1.74409800 0.21446400<br>H 2.87890200 1.65697200 0.73042400<br>H 4.74674000 -2.03643300 -0.56369700<br>H 2.54507000 -2.90752400 -1.15236800<br>H 6.37160100 1.97488100 1.18427600<br>H 4.93049800 2.48210500 0.27494900<br>H 4.76411700 1.85378200 1.93297200<br>H 6.32326000 -1.31604100 0.86545700<br>H 6.54417700 -0.62607600 -0.75778400<br>H 7.26321100 0.18212800 0.65790500<br>H -5.17361100 0.21160600 1.65294200<br>H -6.69202300 0.24465900 0.27157900<br>H -5.59995800 -1.18746600 0.25642600<br>H -4.27235100 2.20472400 -1.61353800<br>H -6.02098600 2.14586000 -0.89625500<br>O -4.65456100 0.57522500 2.73283800<br>H -3.71128300 0.58003400 2.53448500	
**Name**	**MB-C17-C19-OH-FHT**
Cartesian Coordinates	Frequency and Energy
C -3.29983400 -1.71546000 0.03378600<br>C -3.51830900 -0.37234300 -0.37754900<br>C -2.39651300 0.46058000 -0.52041600<br>C -2.03371200 -2.17421500 0.27909500<br>C -1.11611100 -0.01823100 -0.26553800<br>S 0.19411400 1.12316200 -0.46811600<br>N 0.31813800 -1.91926700 0.42789100<br>C -0.88167000 -1.35545300 0.14719600<br>C 1.58693100 0.15183500 -0.04685100<br>C 2.83146900 0.77423700 -0.11819600<br>C 3.97097900 0.05589500 0.19597200<br>C 3.90444700 -1.29271900 0.56618400<br>C 2.67374700 -1.89875300 0.63670500<br>C 1.47064400 -1.21063700 0.33367500<br>N 5.25182100 0.71499000 0.12263000<br>C 5.41710300 1.89808600 0.57582900<br>C 6.35517300 -0.02640900 -0.52099900<br>N -4.79122300 0.09731000 -0.61511000<br>C -5.92610000 -0.68672900 -0.13188800<br>C -4.98646300 1.51277100 -0.73989200<br>H -4.13347200 -2.39071700 0.16300800	Zero-point correction= 0.310313 (Hartree/Particle)<br>Thermal correction to Energy= 0.330578<br>Thermal correction to Enthalpy= 0.331523<br>Thermal correction to Gibbs Free Energy= 0.259559<br>Sum of electronic and zero-point Energies= -1257.477202<br>Sum of electronic and thermal Energies= -1257.456936<br>Sum of electronic and thermal Enthalpies= -1257.455992<br>Sum of electronic and thermal Free Energies= -1257.527956

H -1.88046900 -3.19952600 0.59506400<br>H -2.50761200 1.49112800 -0.83227700<br>H 2.91155400 1.80689800 -0.44187200<br>H 4.79875600 -1.84810700 0.81654000<br>H 2.59347200 -2.93720100 0.93356400<br>H 6.38016400 2.37938100 0.45510400<br>H 4.59959500 2.40002600 1.07937000<br>H 6.64945800 -0.84998200 0.12679400<br>H 5.99485800 -0.40977700 -1.47330300<br>H 7.19155900 0.65152000 -0.66491000<br>H -5.87759200 -0.83399200 0.95208600<br>H -6.84194800 -0.15603000 -0.37878600<br>H -5.95870200 -1.66006600 -0.62130800<br>H -4.39262400 1.94458800 -1.54552400<br>H -6.03836200 1.73371100 -0.90179700<br>H -4.67219100 2.04639700 0.21674400<br>O -3.90353600 2.52949900 1.58747400<br>H -3.14927900 1.91616000 1.52336800		
**Name**	**MB-C17-C20-OH-FHT**	
Cartesian Coordinates	Frequency and Energy	
C 3.69239200 1.37871400 -0.29140900<br>C 3.85297600 -0.03004600 -0.18643400<br>C 2.69274300 -0.81794900 -0.10444200<br>C 2.44201500 1.93792600 -0.32780400<br>C 1.43225800 -0.23867700 -0.14744800<br>S 0.06598100 -1.33044900 -0.04919500<br>N 0.07644400 1.83451600 -0.30239500<br>C 1.25386300 1.16608200 -0.26152900<br>C -1.28743000 -0.22343500 -0.12884000<br>C -2.56184500 -0.78324300 -0.06254300<br>C -3.67052000 0.04298600 -0.10433600<br>C -3.54274000 1.43371600 -0.19643900<br>C -2.28324900 1.97738900 -0.26911400<br>C -1.11049500 1.18040700 -0.23614300<br>N -4.98383500 -0.55021300 -0.03382300<br>C -5.28709300 -1.57493700 -0.73334400<br>C -5.94746500 0.06015200 0.90360200<br>N 5.10235900 -0.60940800 -0.16637500<br>C 6.25366300 0.22544800 0.03012100<br>C 5.21756900 -1.99769900 0.27086600<br>H 4.55363500 2.03040100 -0.33888100<br>H 2.33180400 3.01330900 -0.40529200<br>H 2.76757000 -1.89318600 -0.01018100<br>H -2.68519200 -1.85593300 0.04664100<br>H -4.41549700 2.07257000 -0.23517400<br>H -2.15565900 3.04932200 -0.35671100<br>H -6.26602800 -2.02387000 -0.61581800<br>H -4.56705800 -1.97445400 -1.43770400<br>H -5.45454900 0.17420600 1.86691700<br>H -6.24521500 1.03367700 0.51841300<br>H -6.81279800 -0.59146600 0.98239400<br>H 6.23734100 0.70629900 1.06076900<br>H 7.15972100 -0.37328700 -0.02348400	Zero-point correction= 0.310671 (Hartree/Particle)<br>Thermal correction to Energy= 0.330963<br>Thermal correction to Enthalpy= 0.331907<br>Thermal correction to Gibbs Free Energy= 0.259997<br>Sum of electronic and zero-point Energies= -1257.476295<br>Sum of electronic and thermal Energies= -1257.456003<br>Sum of electronic and thermal Enthalpies= -1257.455059<br>Sum of electronic and thermal Free Energies= -1257.526968	

H	6.30854400	1.03533100	-0.69587200	
H	4.69504600	-2.66411200	-0.41626400	
H	6.26871000	-2.27364600	0.27436100	
H	4.80866700	-2.13355100	1.27798400	
O	5.78216300	1.40576300	2.47477400	
H	4.87851000	1.61037300	2.17370600	
